# Locus-specific proteomics identifies novel regulators of Epstein-Barr virus lytic reactivation

**DOI:** 10.1128/jvi.01408-25

**Published:** 2026-03-17

**Authors:** Daniel E. Greaves, Ildar Gabaev, James C. Williamson, Stuart Bloor, Sergio Martinez Cuesta, Nicholas J. Matheson, Lori Frappier, Jérôme Déjardin, Paul J. Lehner

**Affiliations:** 1 Department of Medicine, University of Cambridge 151904, Cambridge, United Kingdom; 2 Cambridge Institute for Therapeutic Immunology and Infectious Disease (CITIID), University of Cambridge, Cambridge, United Kingdom; 3 Data Sciences and Quantitative Biology, Discovery Sciences, R&D BioPharmaceuticals, Cambridge, United Kingdom; 4 NHS Blood and Transplant 9936, Cambridge, United Kingdom; 5 Department of Molecular Genetics, University of Toronto 204248, Toronto, Canada; 6 Institute of Human Genetics, CNRS-UMR9002-Université de Montpellier 27037, Montpellier, France; Dartmouth College Geisel School of Medicine, Hanover, New Hampshire, USA

**Keywords:** Epstein-Barr virus, locus specific proteomics, epigenetics, virus latency, virus activation, Polycomb complex, NuRD complex, de-ubiquitinase

## Abstract

The Epstein-Barr virus (EBV) is a human gamma-herpesvirus which infects over 90% of the global population and is associated with lymphoid and epithelioid cancers. After infection, EBV enters a latent state in B cells, whereby the viral genome persists as a nuclear episome maintained by expression of a small number of latency-associated viral proteins. The lytic viral proteins, required for DNA replication and virion production, are silenced by cellular epigenetic mechanisms. The immediate-early lytic gene BZLF1 is the most important target for transcriptional repression, as its expression triggers the lytic cascade. To gain insight into the factors restricting BZLF1 expression, we used the PICh method of locus-specific proteomics (proteomics of isolated chromatin segments) to identify proteins which occupy BZLF1 promoter DNA. We identified more than 30 proteins associated with the BZLF1 promoter, including the nucleosome remodeler CHD4 and components of the Polycomb PRC1 complex. We show that CHD4 and PRC1 components are novel repressors of BZLF1 gene expression and that both are required to prevent spontaneous lytic reactivation in Burkitt lymphoma cells. We also reveal a marked, cell-wide loss of the PRC1 histone mark (H2AK119Ub) during the lytic cycle, which is dependent on immediate-early and early lytic gene expression. A proteomic analysis of Burkitt lymphoma cells containing lytic EBV identified upregulation of USP17, a de-ubiquitinase capable of H2AK119Ub removal. Taken together, our study demonstrates the power of proteomic approaches to identify repressors of EBV reactivation and provides new insight into how EBV manipulates epigenetic mechanisms during the lytic cycle.

**IMPORTANCE:**

Following infection, Epstein-Barr virus persists in a latent state where the viral genome resides in the cell nucleus as an episome. Cellular epigenetic proteins occupy the episomes to restrict viral gene expression and prevent lytic reactivation. In this study, we use mass spectrometry to characterize cellular proteins occupying a key viral lytic gene promoter (pBZLF1). We identify the nucleosome remodeler CHD4 and histone modifier complex PRC1 as novel repressors of pBZLF1 and show that both are required to prevent spontaneous EBV lytic reactivation in B cells. We also report that PRC1-mediated histone modification is erased during EBV lytic reactivation from both cellular and viral genomes. The human de-ubiquitinase enzyme USP17 is likely to be responsible for this effect, as upregulation of USP17 is induced by EBV lytic proteins. This study provides new insight into how EBV manipulates epigenetic mechanisms to regulate latency and lytic reactivation and reveals novel potential therapeutic targets.

## INTRODUCTION

The Epstein-Barr virus (EBV) is a human herpesvirus that infects over 90% of the human population and is strongly associated with lymphoid and epithelioid cancers (1), including Hodgkin lymphoma, post-transplant lymphoproliferative disorder, nasopharyngeal carcinomas, and a subset of gastric cancers. EBV-associated malignancies are thought to account for approximately 1–1.5% of cancers worldwide (2). EBV, like other herpesviruses, enters a latent state after infection which persists for the lifetime of the host. Although low-level reactivation of the virus periodically occurs, this does not generally result in clinical symptoms. The oncogenic potential of EBV comes from viral proteins expressed during latent infection which provoke uncontrolled proliferation of the host cells and can result in malignant transformation.

EBV initially infects oropharyngeal epithelial cells and is then transmitted to B cells circulating through the oral cavity. In infected B cells, the EBV genome enters the nucleus where it is circularized and chromatinized (3). A coordinated sequence of viral protein expression then occurs as the infected cells progress through different stages of viral latency. In early latency (stages IIb and III), expression of multiple viral genes, including EBNA2, EBNA 3A, B & C, EBNALP, and LMP1/2, transforms the B cells into proliferating lymphoblasts. These cells are highly immunogenic and targeted by cytotoxic T-lymphocytes (CTL), favoring selection of a more restrictive latency pattern. The combination of immune selection and passage of infected cells through lymph node germinal centers eventually leads to the establishment of latent EBV in a small pool of memory B cells. These cells have a latency type I pattern, in which expression from the viral genome is minimal and consists only of non-coding RNAs and the EBNA1 protein, which tethers the EBV genome to host chromatin.

A key feature of the EBV life cycle is the ability to switch between latent and lytic infection. Expression of lytic genes occurs in a temporal sequence and begins with expression of the immediate-early gene BZLF1. Maintaining the virus in a latent state is therefore critically dependent on repression of this gene, so the chromatin state of the BZLF1 promoter (pBZLF1) is of central importance for maintaining latent infection. Multiple silencing mechanisms coexist, with some redundancy as disruption of a single component results in only a fraction of cells entering the lytic cycle. The histone marks occupying pBZLF1 are characteristic of repressive chromatin and include the H3K27me3 mark deposited by the PRC2 Polycomb complex (4–6), as well as H3K9me3, maintained by the TRIM28-SETDB1 axis (5, 7–9). H3K9me3 appears to be less important for repression than H3K27me3, as it is not removed upon lytic reactivation (5, 10). Histone deacetylation of lytic-stage promoters, including pBZLF1, is seen during latent infection and is reversed when the virus enters the lytic cycle (5, 11). pBZLF1 is also targeted by sequence-specific DNA-binding proteins, including ZEB1/2, MEF2D, and JDP2, which recruit histone deacetylase (HDAC) enzymes (12–15). The linker histone H1, which is associated with nucleosome compaction, occupies pBZLF1 and also acts as a repressor (16). The higher-order chromatin structure of the latent EBV genome also maintains silencing of pBZLF1: the transcription factor MYC prevents looping between pBZLF1 and the origin of lytic replication, OriLyt, to restrict OriLyt-mediated enhancer activity (17).

How the latent-lytic switch occurs *in vivo* is not fully understood, but the most likely physiological triggers are B-cell receptor (BCR) signaling and plasma cell differentiation (18). BCR activation triggers a signaling pathway that culminates in binding of a series of transcription factors to pBZLF1, including CREB, C/EBP-beta, ATF-1, XBP1, and EGR1 (19–23). Plasma cell differentiation increases the levels of host factors BLIMP1 and XBP1s, both of which also induce BZLF1 expression (17, 24, 25), triggering a cascade of approximately 30 early lytic genes, whose function includes formation of replication compartments within the nucleus and viral DNA replication. Following DNA replication, around 30 late lytic genes are then expressed which coordinate virion formation and secretion.

The sequence of pBZLF1 has been defined and extensively investigated using candidate gene approaches, but an unbiased assessment of the chromatin proteins associated with pBZLF1 has not been undertaken and could identify new repressors. Mass spectrometry-based methods of “locus-specific proteomics” allow purification of proteins bound to specific DNA sequences of interest and include PICh (proteomics of isolated chromatin segments), in which custom-made nucleic acid probes are used to perform a pulldown of both the target DNA sequence and associated proteins (26). PICh has been successfully used to profile multiple genomic loci and identified both known and novel binding proteins (26–29). To gain an unbiased view of chromatin proteins associated with pBZLF1, we performed PICh on pBZLF1 and identified binding of the nucleosome remodeler CHD4 and the Polycomb complex PRC1. Knockdown experiments confirmed that both repressors are required to maintain EBV latency in Akata B-cells. Furthermore, we found that a complete cellular loss of the PRC1 histone mark H2AK119Ub occurs during viral lytic reactivation, and this loss is dependent on expression of EBV immediate-early and early lytic genes. Proteomic analysis of cells undergoing lytic EBV infection identified upregulation of the human de-ubiquitinase USP17 as the likely mechanism by which H2AK119Ub is removed.

## RESULTS

### Generation of a pBZLF1-GFP reporter system compatible with proteomics of isolated chromatin segments (PICh)

The minimal promoter of BZLF1 is well defined and has binding sites for both transcriptional repressors and activators mapped using *in vitro* methods, e.g., EMSA (12, 18, 30, 31). Advances in proteomics now allow chromatin composition at individual genomic loci to be characterized *in vivo*, with the ability to systematically identify key regulators at specific loci without making *a priori* assumptions. However, despite an enrichment factor of ~10,000-fold (32), PICh still requires a highly abundant target locus, and a single 1 kb locus requires ~200 copies per cell. EBV is present at ~10–60 copies per cell in most B-cell lines, and it was not therefore possible to perform PICh over pBZLF1 in cells containing wild-type latent EBV, requiring an alternative approach.

**Fig 1:**
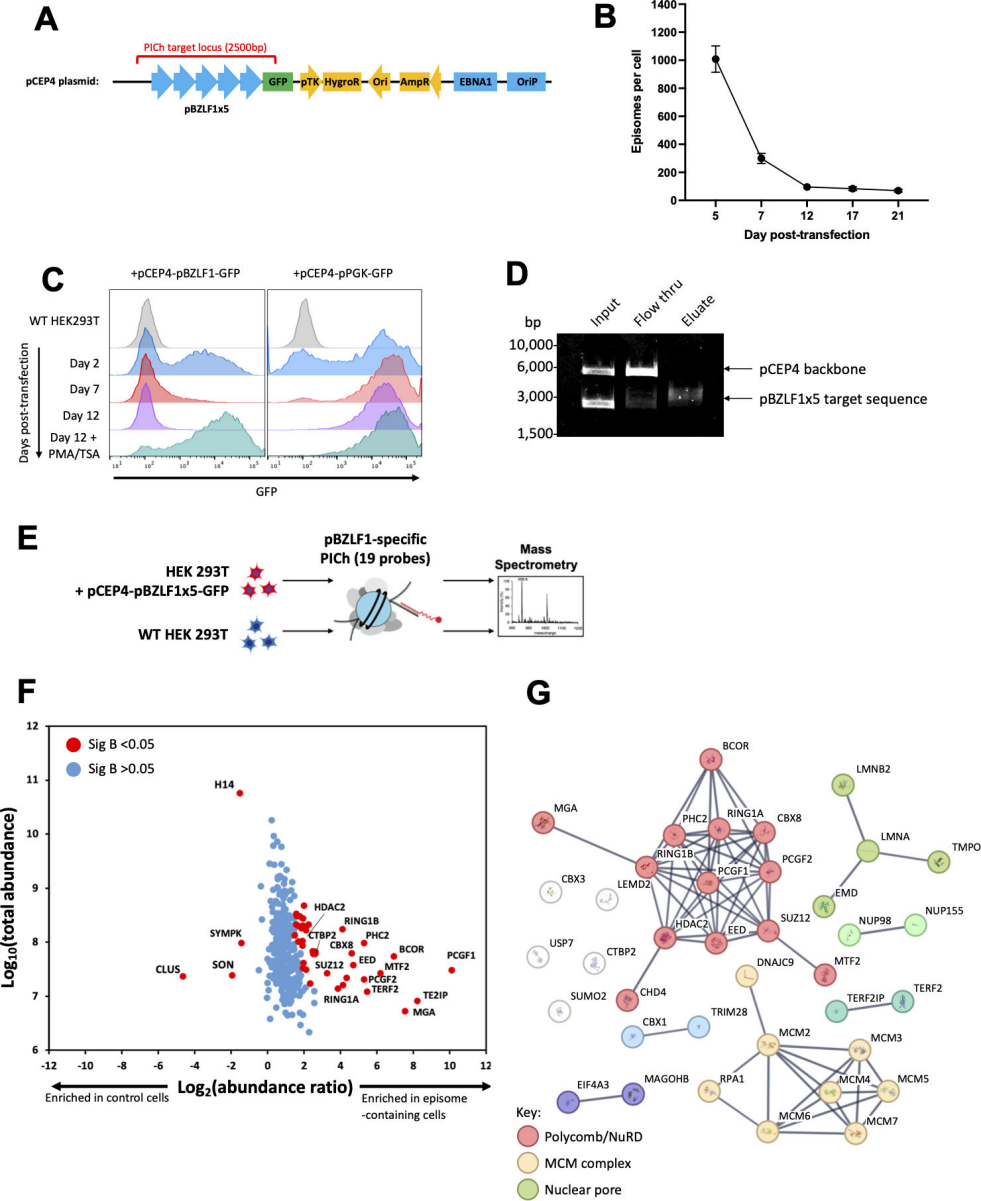
Proteomics of isolated chromatin segments (PICh) identifies binding of CHD4 and PRC1 to EBV BZLF1 promoter. (**A**) Schematic diagram of the pCEP4-pBZLF1(x5)-GFP reporter system compatible with PICh analysis. Five copies of pBZLF1 were cloned upstream of the GFP coding sequence into the pCEP4 episomal vector, which contains EBNA1 and OriP elements, as well as antibiotic resistance cassettes. The PICh target locus is indicated by a red bracket. (**B**) RT-qPCR analysis of the episome copy number. HEK293T cells were transfected with pCEP4-pBZLF1(x5)-GFP plasmid, harvested at indicated time points, and subjected to DNA extraction and RT-qPCR analysis with HygroR-specific primers. Episome copy number per cell was calculated using absolute quantitation, with dilutions of prepared plasmid DNA as standards. Cellular DNA was normalized to GAPDH. Error bars denote variance between three technical replicates. (**C**) pBZLF1(x5)-GFP reporter is progressively silenced following transfection into HEK293T cells. Cells were transfected with pCEP4-pBZLF1(x5)-GFP reporter or pCEP4-pPGK-GFP control plasmid, selected with hygromycin starting at day 2, harvested at indicated time points, and analyzed by flow cytometry. At day 12, following transfection, cells were stimulated with a combination of PMA, A23187, and trichostatin A (TSA) and analyzed by flow cytometry 24 h later. (**D**) PICh probes retrieve the target locus DNA in plasmid capture assay. The pCEP4-pBZLF1(x5)-GFP plasmid was treated with *BssHII* and *NotI* endonucleases to separate the target locus from the backbone (input). The digested DNA was then incubated with an equimolar mix of the 19 PICh probes, and probe-DNA hybrids were retrieved using streptavidin beads (eluate). The supernatant in the probe formed upon magnetic bead separation was collected as flow-through. 1/10 of each sample was resolved on agarose gel. (**E**) Schematic diagram of pBZLF1 PICh experiment. HEK293T cells harboring the repressed pCEP4-pBZLF1(x5)-GFP episomes and control untransfected HEK293T cells were subjected to chromatin isolation, followed by tiling with an equimolar mix of 19 PICh probes. Protein eluates from both samples were analyzed by mass spectrometry. (**F**) Distribution of protein abundance ratios (episome-containing/control cells) vs total protein abundance for 357 nuclear proteins identified in the pBZLF1 PICh experiment. Outlier protein abundance ratios (Significance B < 0.05) are highlighted (red dots). (**G**) STRINGDB analysis of the 39 nuclear proteins significantly enriched in the cells harboring the pCEP4-pBZLF1(x5)-GFP episome. K-means clustering was used to separate proteins into categories, with three clear groups identified (see key). Network edges between nodes represent high confidence interactions (>0.9).

We therefore developed a plasmid-based model system of pBZLF1 silencing in HEK 293T cells. These cells were chosen because they are highly permissive to transfection and were capable of maintaining plasmids at high copy number. To maximize the amount of target material for PICh, pBZLF1 was multimerized 5-fold and cloned into a pCEP4 vector encoding EBV EBNA1 and OriP elements, which ensure maintenance as a nuclear episome, similar to EBV (Fig. 1A). The pBZLF1x5 –GFP reporter was lipofected into HEK293T cells and 12 days post-transfection the copy number was ~100 copies per cell, as measured by RT-qPCR (Fig. 1B). A GFP coding sequence inserted downstream of pBZLF1x5 enabled measurement of promoter activity by flow cytometry. As expected, the GFP signal was progressively silenced following transfection into HEK 293T cells (Fig. 1C) but was restored following treatment with a combination of the protein kinase C activator PMA and histone deacetylase inhibitor TSA (Fig. 1C) or by overexpression of BZLF1 protein (Fig. S1A). By contrast, the same vector containing GFP under the control of the phosphoglycerate kinase (pPGK) promoter showed no loss of GFP signal after transfection, implying that the silencing process was specific to pBZLF1 (Fig. 1C). To ensure biological relevance, the plasmid was also electroporated into EBV-negative Akata cells, where a similar process of reversible pBZLF1-GFP silencing was observed (Fig. S1B). The episome copy number per cell for EBV-negative Akata was approximately 40 episomes per cell, which was lower than HEK 293T and considered to be insufficient for PICh (Fig. S1C)

The combination of high episome copy number in HEK293T cells and multimerized pBZLF1 provided sufficient PICh target material, so we designed a series of 19 desthiobiotinylated 2′ F-RNA probes to tile over the pBZLF1x5 sequence with a 300 bp overlap into the vector on each side. To confirm specific capture of the target sequence, the target was excised from the backbone vector using restriction enzymes, and both target and empty backbone were incubated with the desthiobiotinylated probes *in vitro*. Streptavidin bead pulldown of the probe-DNA hybrids selectively retrieved only the pBZLF1x5 target sequence (Fig. 1D).

### Proteomics of isolated chromatin segments (PICh) identifies binding of CHD4 and Polycomb PRC1 to pBZLF1

Having confirmed the probe specificity, the PICh experiment was performed in HEK 293T cells harboring the silenced pBZLF1-GFP episome using wild-type HEK 293T cells as the control (Fig. 1E). Chromatin was then extracted from 4 × 10^9^ cells and probed with an equimolar mix of all 19 probes. Following streptavidin bead pulldown and extensive washing, samples were subjected to mass spectrometry. A total of 1,363 proteins were identified by ≥2 peptides, of which 357 were localized to the nucleus based on GOCC annotation. To determine which of these proteins were specifically bound to pBZLF1, we compared their relative abundances from cells with and without the silenced pBZLF1-GFP episome. This revealed 39 nuclear proteins to be significantly enriched in the episome-containing cells (Significance B < 0.05) (Fig. 1F; Tables S1 and S2) (33). Cluster analysis of the enriched proteins using STRING-DB identified three main groups: (i) transcriptional repressors associated with the Polycomb and Nucleosome Remodeling and Deacetylation (NuRD) complexes, (ii) proteins associated with the Mini Chromosome Maintenance (MCM) complex, and (iii) structural proteins related to the nuclear pore (Fig. 1G). The identification of components of the Polycomb complex increased our confidence that the PICh experiment had successfully retrieved the pBZLF1 DNA, as PRC2 is a major repressor of latent EBV (4–6). Further evidence suggesting that the PICh experiment was pBZLF1-specific included: (i) enriched proteins contained the two reported pBZLF1 repressors, HDAC2 (34) and TRIM28 (9, 35); (ii) the MCM complex interacts with the EBV OriP sequence present on the episome upstream of pBZLF1 (36, 37); and (iii) TRF2, a telomere-interacting protein, is also reported to bind OriP (38, 39). Of particular interest among the transcriptional repressors was the nucleosome remodeler CHD4 and the multiple highly enriched components of the Polycomb PRC1 complex: RING1A, RING1B, PCGF1, PCGF2, PHC2, BCOR, and MGA. Both CHD4 and PRC1 have well-described roles in silencing of mammalian genes, through nucleosome compaction and histone 2A ubiquitination, respectively, but neither is a known repressor of pBZLF1, so they were investigated further.

### CHD4 and PRC1 repress the pBZLF1-GFP reporter in EBV-negative Akata Burkitt lymphoma cells

**Fig 2:**
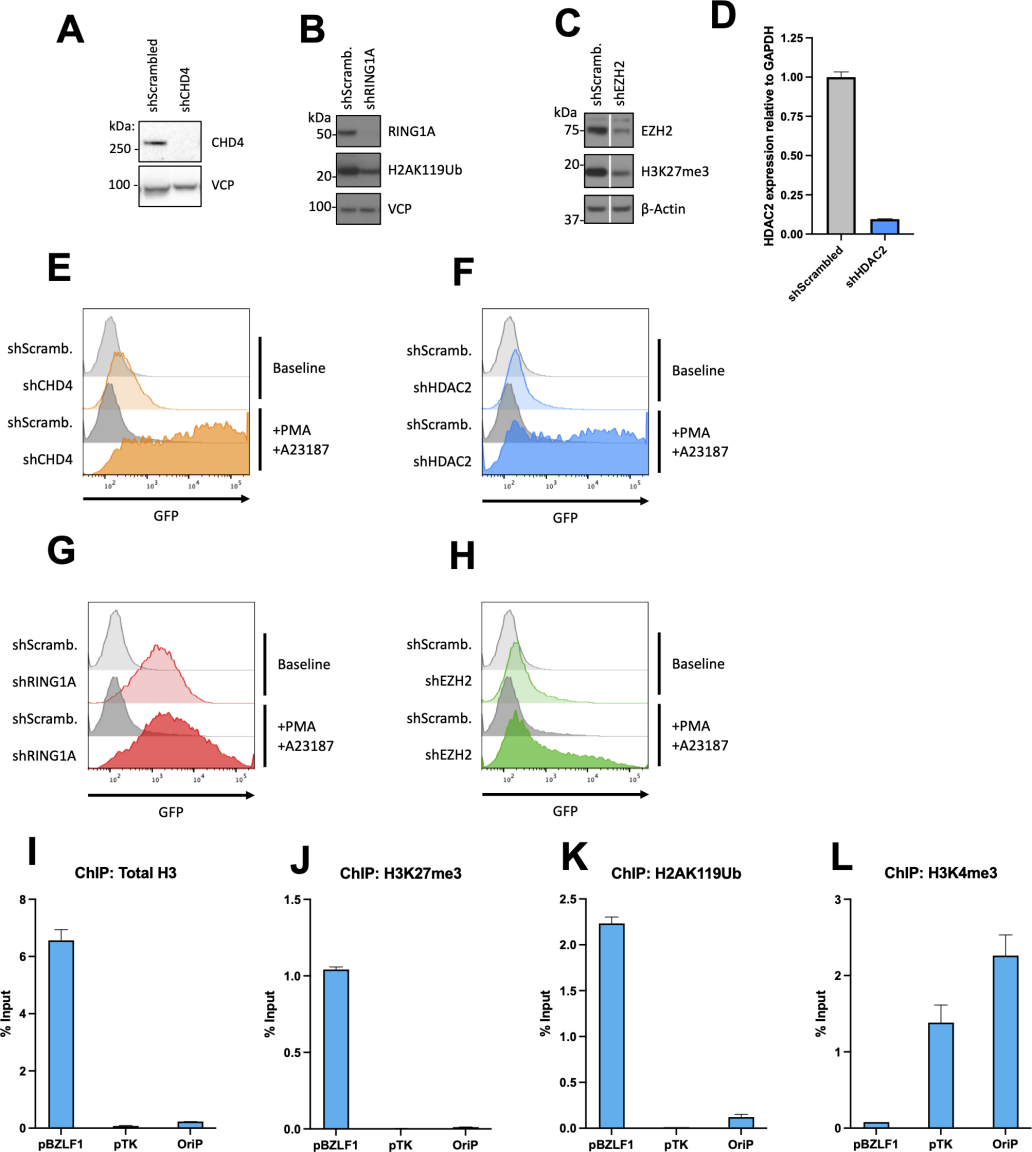
CHD4 and PRC1 repress the pBZLF1(x5)-GFP reporter in EBV-negative Akata Burkitt lymphoma cells. (**A–C**) shRNA-mediated knockdowns of CHD4 (**A**), RING1A (**B**), and EZH2 (**C**). Akata cells were transduced with lentiviruses expressing the indicated shRNAs and control scrambled shRNA, lysed at day 5, and analyzed by immunoblot with the specific antibodies. (**D**) shRNA-mediated knockdown HDAC2. Akata cells were processed as described in panels **A–C** and subjected to RT-qPCR analysis with primers specific for *HDAC2* and *GAPDH*. Data are presented as mean of *n* = 3 technical replicates ± SD. (**E–H**) Flow cytometry analysis of GFP expression in EBV-negative Akata cells harboring the pBZLF1(x5)-GFP reporter and expressing the indicated shRNAs from panels **A–D**. PMA and A23187 were added 24 h prior to flow cytometry analysis. (**I–L**) ChIP-qPCR analysis of EBV-negative Akata cells harboring pCEP4-pBZLF1(x5)-GFP reporter construct using the indicated antibodies.

We selected the following hits for validation: CHD4, HDAC2 (components of the NuRD complex), and RING1A (the dominant enzymatic component of PRC1 in Akata and Raji Burkitt lymphoma cell lines, Fig. S2). As a comparator, the PRC2 enzymatic component EZH2, which is a known repressor of pBZLF1 (4, 5), was also validated. shRNAs were designed against the target proteins, and the efficacy of knockdowns was confirmed by immunoblot analysis for CHD4, RING1A, and EZH2 (Fig. 2A through C) and by RT-qPCR for HDAC2 (Fig. 2D). Notably, knockdowns of RING1A and EZH2 also resulted in reduction of the PRC1 histone mark (H2AK119Ub) and the PRC2 histone mark (H3K27me3), respectively (Fig. 2B and C). To ensure biological relevance for EBV, shRNA knockdown experiments were then performed in EBV-negative Akata Burkitt lymphoma cells electroporated with the pBZLF1-GFP reporter plasmid. We then set out to analyze GFP expression in the cells with knockdowns and additionally treated the portions of transduced cells with PMA. Flow cytometry analysis showed that CHD4 depletion increased GFP expression compared with the scrambled shRNA control, and a further increase was seen upon stimulation of the cells with PMA (Fig. 2E). Depletion of HDAC2 had minimal effect on GFP expression at baseline but also showed a marked increase in GFP expression following PMA stimulation (Fig. 2F). These results suggested that CHD4 and HDAC2 had overlapping roles in antagonizing PMA-mediated stimulation of pBZLF1 expression, while CHD4, but not HDAC2, was required for silencing spontaneous pBZLF1 expression. Depletion of RING1A was sufficient to significantly increase pBZLF1-GFP expression (Fig. 2G), whereas, by comparison, depletion of the PRC2 enzyme EZH2 led to only a very small increase in GFP expression (Fig. 2H). The addition of PMA substantially increased GFP expression in both the RING1A-depleted and EZH2-depleted cells (Fig. 2G and H). Like CHD4, RING1A may therefore play a role in repressing spontaneous pBZLF1-GFP expression, whereas EZH2 is primarily involved in silencing PMA-induced pBZLF1-GFP expression.

To determine pBZLF1 occupancy of the Polycomb repressors, we performed ChIP-PCR. This confirmed occupation of pBZLF1 by both H2AK119Ub (PRC1) and H3K27me3 (PRC2), compared with transcriptionally active regions of the episome, where the Polycomb marks were not identified (Fig. 2I through L). Likewise, PRC1 and PRC2 did not occupy the active pGK promoter driving GFP (Fig. S3A through D). In summary, these experiments suggest that the pBZLF1 promoter is silenced by the NuRD complex proteins CHD4 and HDAC2, as well as PRC1 core protein RING1A, and provide further support that the PICh experiment was pBZLF1-specific.

### CHD4 is a repressor of spontaneous lytic reactivation in Akata cells

**Fig 3:**
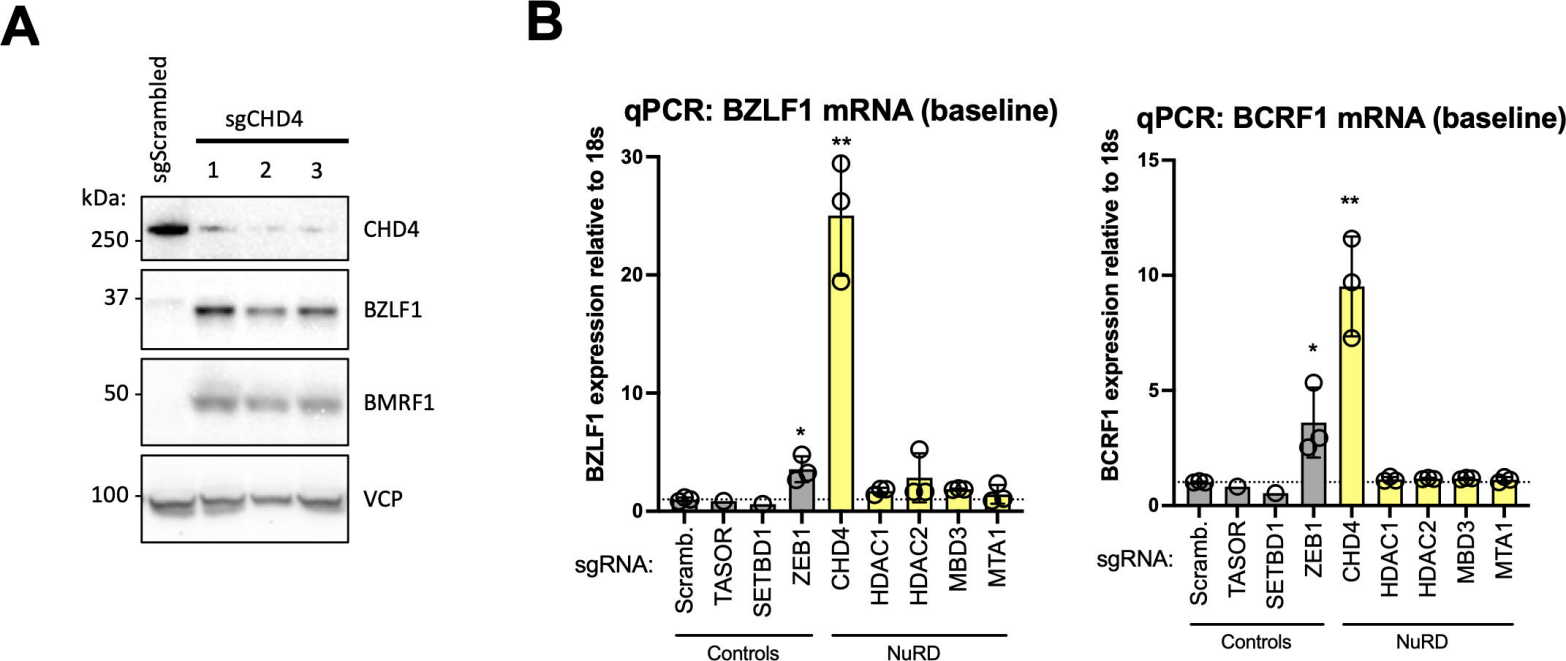
CHD4 is a repressor of spontaneous EBV lytic reactivation in Akata cells. (**A**) CRISPR-Cas9-mediated depletion of CHD4 results in expression of lytic EBV gene products. EBV-positive Akata Cas9 cells were transduced with lentiviruses expressing CHD4-specific sgRNAs, lysed, and subjected to immunoblot analysis with antibodies specific for CHD4, BZLF1, BMRF1, and VCP. (**B**) Lytic EBV gene expression is specifically triggered by depletion of CHD4, but not of other components of the NuRD complex. RT-qPCR analysis of BZLF1 (left) and BCRF1 (right) mRNA expression in EBV-positive Akata Cas9 cells harboring sgRNAs specific for the indicated genes. The cells were transduced with lentiviruses expressing indicated sgRNAs and subjected to RNA extraction 7 days following transduction. Three independent sgRNAs were used per gene, except for TASOR and SETDB1 (negative controls), where a single sgRNA was used. ZEB1 was used as a positive control, being a well-described repressor of EBV lytic reactivation. For each targeted protein, a paired two-tailed Student’s *t*-test was used to assess statistical significance versus the scrambled control (*P value < 0.05, **P value < 0.01). Data are presented as mean of *n*  = 3 biological replicates ± SD.

Having confirmed that CHD4 is required for silencing the pBZLF1-GFP reporter in EBV-negative Akata cells, we wanted to determine its role in repressing EBV lytic reactivation. We therefore used CRISPR-Cas9 to delete CHD4 from EBV-positive Akata cells and examined expression of EBV lytic genes. Three independent CRISPR sgRNAs targeting CHD4 all provoked expression of both the immediate-early lytic gene BZLF1 and early gene BMRF1 in Cas9^+^ Akata cells (Fig. 3A). CHD4 depletion also increased late lytic gene BCRF1 expression, suggesting that full lytic reactivation was triggered (Fig. 3B, right panel). To investigate whether CHD4 acted alone to repress EBV, or as part of the NuRD complex, four additional NuRD components were also targeted by CRISPR: HDAC1, HDAC2, MBD3, and MTA1. All four proteins were identified in the PICh experiment, although only HDAC2 was significantly enriched. Three sgRNAs targeting ZEB1 were used as a positive control, as this protein is a well-described repressor of BZLF1 (30, 40). As negative controls, three scrambled sgRNAs were used, along with a single sgRNA each for TASOR and SETDB1, chromatin modifiers not involved in EBV repression. Compared with the negative controls, only CHD4 deletion increased spontaneous BZLF1 mRNA expression (Fig. 3B, left panel), suggesting that under the conditions used, CHD4 was the only NuRD complex component involved in EBV repression. How CHD4 is recruited to the EBV genome and whether it suppresses spontaneous lytic reactivation alone or as part of a multi-protein complex requires further examination.

### Polycomb PRC1 represses BZLF1 expression and occupies the EBV lytic gene promoters in a PRC2-independent manner

**Fig 4:**
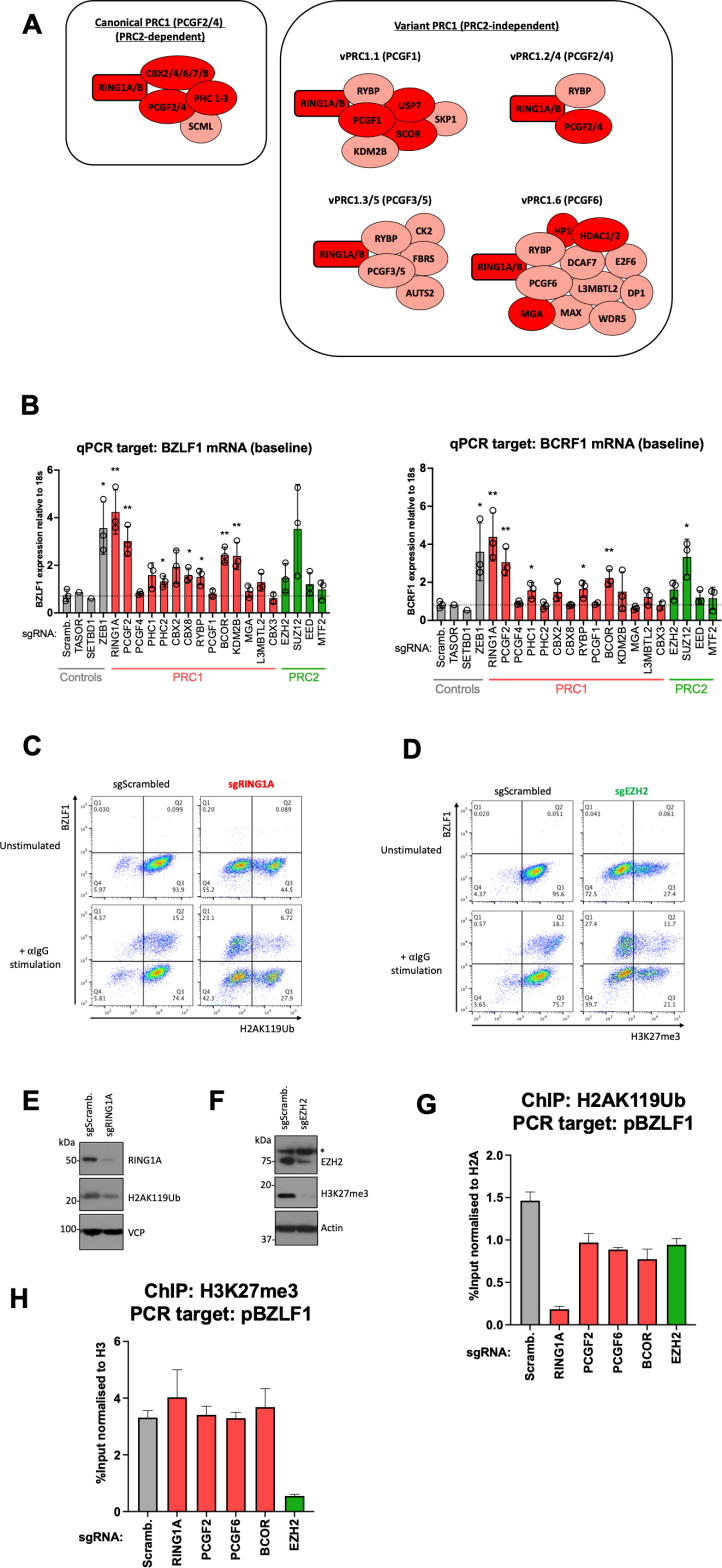
Polycomb PRC1 represses BZLF1 expression and occupies EBV lytic gene promoters in a PRC2-independent manner. (**A**) Schematic diagram of Polycomb PRC1 subcomplexes. A ubiquitin E3 ligase (either RING1A or B – rectangular box) is constitutively present along with one of the six PCGF proteins. Canonical PRC1 complexes contain CBX and PHC proteins, whereas variant PRC1 complexes contain RYBP plus additional proteins required for targeting to specific genomic elements. Proteins recovered from the PICh experiment are highlighted in red. (**B**) Expression of EBV lytic *BZLF1* (left) and *BCRF1* (right) genes in cells with CRISPR-mediated depletion of cellular repressors. EBV-positive Akata Cas9^+^ cells were transduced with lentiviruses encoding sgRNAs specific for the indicated cellular targets, cultured for 7 days, followed by RNA extraction and RT-qPCR analysis. Three independent sgRNAs were used per gene, except for TASOR and SETDB1 (negative controls) where a single sgRNA was used. For each target protein, a paired two-tailed Student’s *t*-test was used to assess statistical significance versus the scrambled control (*P value < 0.05, **P value < 0.01). Data are presented as mean of *n* = 3 biological replicates ± SD. (**C, D**) Flow cytometry analysis of BZLF1, H2AK119Ub (the PRC1 histone mark), and H3K27me3 expression in EBV-positive Akata cells harboring an sgRNA against either RING1A or EZH2. The cells were transduced with lentiviruses expressing indicated sgRNAs or control scrambled sgRNA, cultured for 7 days, and stimulated with anti-IgG antibody for 24 h, followed by intranuclear staining with antibodies specific for BZLF1, H2AK119Ub, and H3K27me3. (**E and F**) Immunoblots for the indicated proteins in EBV-positive Akata cells expressing an sgRNA against either RING1A (**E**) or EZH2 (**F**). Asterisk denotes non-specific bands. (**G**) ChIP-qPCR using an antibody to H2AK119Ub in EBV-positive Akata cells expressing sgRNA against the indicated proteins. qPCR analysis was performed with pBZLF1 (BZLF1 promoter)-specific primers. Data are presented as mean of *n* = 3 technical replicates ± SD. (**H**) ChIP-qPCR analysis was performed as seen in panel **G** using an antibody against H3K27me3.

Polycomb PRC1 is structurally diverse, consisting of a core assembly of RING1A or B plus one of the six PCGF proteins. The PCGF proteins determine subsequent interactions with other PRC1-associated proteins, which in turn determine how the complex is recruited to the genome. Classically, the so-called “canonical” PRC1 complexes containing PCGF2/4 are recruited to areas of the genome occupied by PRC2, whereas the “variant” PRC1 complexes containing PCGF1/3/5/6 are PRC2-independent and recruited by transcription factors or to genomic features such as CpG islands (Fig. 4A). As our PICh experiment identified multiple PRC1 components from both canonical and variant subcomplexes, we used CRISPR-Cas9 in EBV-positive Akata cells to determine how depletion of different PRC1-associated proteins affected spontaneous BZLF1 expression. Three independent sgRNAs were designed for each of the following: the PRC1 E3 ubiquitin ligase RING1A; canonical PRC1 components PCGF2, PCGF4, PHC1, PHC2, CBX2, and CBX8; and variant PRC1 components RYBP, BCOR, KDM2B, MGA, L3MBTL2, and CBX3. As a comparator, sgRNAs were also designed to target PRC2 components EZH2, SUZ12, EED, and MTF2. SgRNAs targeting ZEB1 were used as a positive control, whereas scrambled sgRNAs, TASOR, and SETDB1 were used as negative controls. The sgRNAs targeting RING1A resulted in a ~4-fold increase in BZLF1 expression, while a smaller, but still significant, increase was seen in cells depleted of canonical PRC1 components PCGF2 and PHC2 and the variant PRC1 components CBX8, RYBP, BCOR, and KDM2B (Fig. 4B, left panel). An increase in spontaneous expression of the late EBV-viral gene BCRF1 was also observed, indicating that the sgRNA-expressing cells had entered the full lytic cycle (Fig. 4B, right panel). Following treatment of Akata cells with anti-IgG to stimulate lytic reactivation, intracellular EBV genomic DNA significantly increased in cells depleted of RING1A, the canonical PRC1 components PCGF2 and PHC1, and the variant components RYBP, PCGF, BCOR, and MGA (Fig. S4A). RING1A-depleted Akata cells were also analyzed by flow cytometry with co-staining for BZLF1 and H2AK119Ub (Fig. 4C). This also showed an increase in lytic reactivation following anti-IgG treatment in cells lacking H2AK119Ub, indicating a synergistic effect between RING1A depletion and anti-IgG. The RING1A knockdown used for flow cytometry was confirmed by immunoblot (Fig. 4E). Taken together, these results suggest that PRC1 is required for EBV latency in Akata cells and that both canonical and variant PRC1 subcomplexes are likely to be involved. By comparison, depletion of PRC2-associated proteins (EZH2, SUZ12, EED, and MTF2) had a small effect on spontaneous BZLF1 expression (Fig. 4B, left) but did show a synergistic effect when combined with anti-IgG (Fig. S4A, D and F). Occupancy of EBV lytic gene promoters by H2AK119Ub and H3K27me3 was confirmed using ChIP-PCR (Fig. S4B through E). To investigate how PRC1 was recruited to the EBV genome, we performed ChIP-PCR with H2AK119Ub- and H3K27me3-specific antibodies in EBV-positive Akata Cas9 cells following depletion of the core PRC1 subunit RING1A, members of both canonical and variant PRC1 pathways (PCGF2, PCGF6, BCOR), and the PRC2 methyltransferase EZH2. Disruption of RING1A or EZH2 markedly reduced H2AK119Ub or H3K27me3 occupancy, respectively (Fig. 4G and H). In contrast, depletion of canonical or variant PRC1 components or PRC2 (EZH2) only partially reduced H2AK119Ub occupancy of pBZLF1 promoter, consistent with a multifactorial recruitment mechanism at least partially independent of PRC2 (Fig. 4G). At the same time, no reduction in H3K27me3 occupancy of pBZLF1 promoter was observed upon depletion of RING1A, PCGF2, PCGF6, or BCOR in these cells (Fig. 4H), suggesting that PRC2 recruitment to pBZLF1 was, in turn, independent of PRC1. Altogether, our data show that BZLF1 expression from EBV episomes is repressed not only by PRC2, but also by PRC1, and that this repression is mutually independent. Furthermore, PRC1 occupancy of pBZLF1 is independent of PRC2.

### The PRC1 histone mark H2AK119Ub is depleted from both EBV and human loci during EBV lytic reactivation

**Fig 5:**
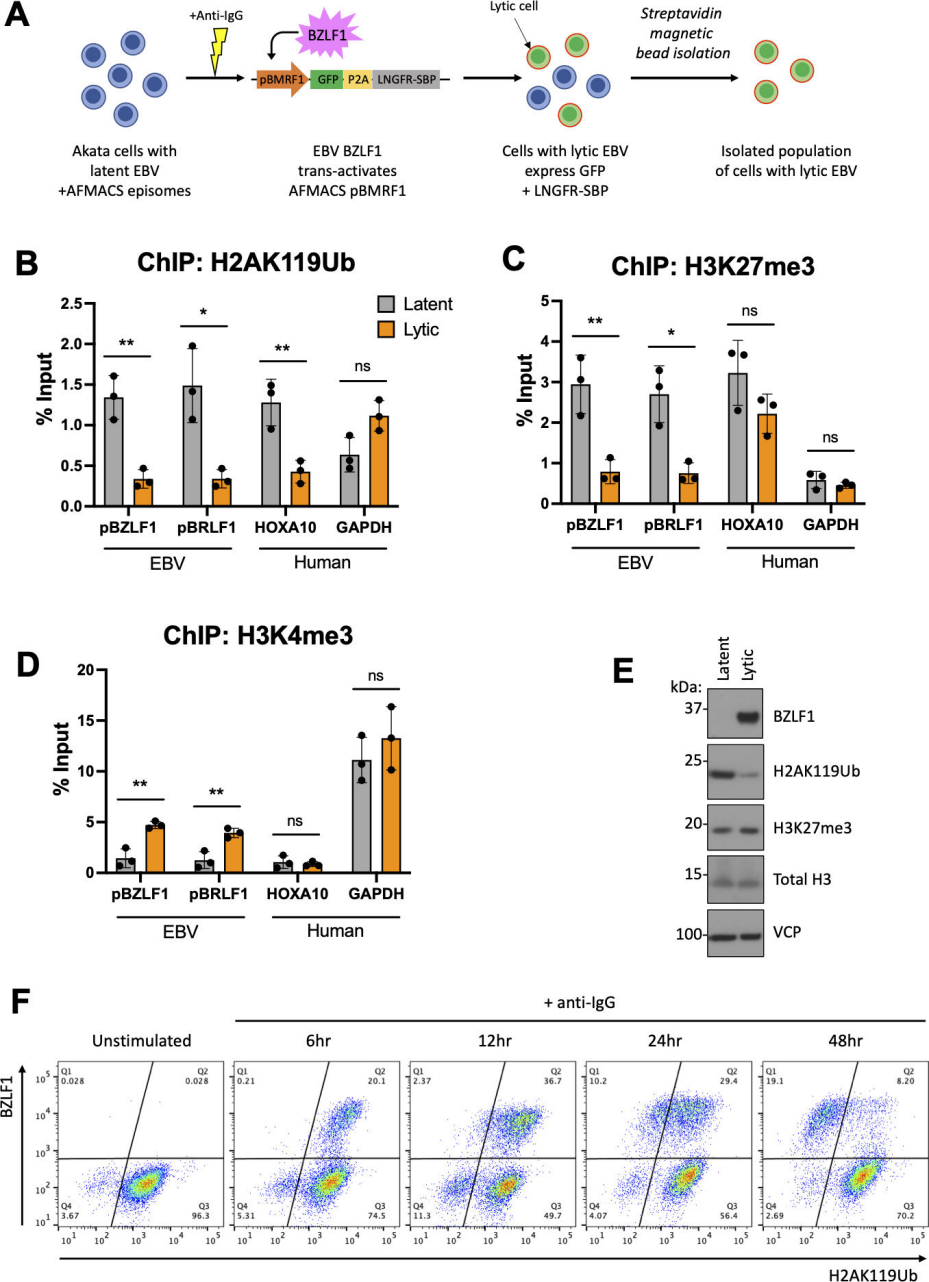
H2AK119Ub is depleted from both EBV and human loci during lytic reactivation. (**A**) Workflow schematic for isolation of Akata cells with lytic EBV using AFMACS enrichment system. Akata cells harboring latent EBV and pCEP4 episomal vector with pBMRF1-GFP-P2A-LNGFR-SBP cassette were stimulated with anti-IgG antibody for 12 h. The cells with lytic EBV (positive for both LNGFR-SBP and GFP) were then isolated using streptavidin magnetic beads. (**B–D**) ChIP qPCR analysis with antibodies specific for H2AK119Ub, H3K27me3, and H3K4me3 histone marks in EBV-positive Akata cells. The lytic cycle of EBV was induced as described for panel A. 200 µM acyclovir was added to cells prior to stimulation to prevent viral genome replication. The comparison is made between AFMACS-enriched Akata cell population harboring lytic EBV (orange bars) and unstimulated cells harboring latent EBV (gray bars) for both viral (BZLF1 and BRLF1 promoter regions) and human (HOXA10, GAPDH) loci (*P value < 0.05, **P value < 0.01, ns: not significant [*P* >0.05]). (**E**) Immunoblot analysis of Akata cells with latent and lytic EBV. The cells were treated and enriched by AFMACS as outlined for panel A or left untreated, lysed, and subjected to immunoblot analysis with antibodies specific for BZLF1, H2AK119Ub, H3K4me3, H3, and VCP. (**F**) Flow cytometry analysis of EBV-positive Akata cells upon anti-IgG stimulation. The cells were treated with anti-IgG antibody or left untreated, fixed at indicated time points, permeabilized, and stained with antibodies specific for BZLF1 and H2AK119Ub.

The loss of repressive histone marks, such as H3K27me3, from the EBV genome during lytic reactivation is well described (5), making it important to determine whether this was also true for H2AK119Ub. This analysis was challenging as it requires isolation of a pure population of lytic EBV-positive Akata cells for ChIP-PCR. We therefore took advantage of antibody-free magnetic cell sorting (AFMACS), in which a low-affinity nerve growth factor receptor (LNGFR)-streptavidin binding protein (SBP) fusion protein expressed at the cell surface enables selection with streptavidin-coated magnetic beads (41, 42). Using the pCEP4 episomal vector as a backbone, we generated an AFMACS system to enrich cells containing lytic EBV, in which the EBV BMRF1 promoter drives expression of GFP and LNGFR-SBP as a single polypeptide, separated by a P2A sequence. The BMRF1 promoter remains silent until transactivated by BZLF1 during the early EBV lytic cycle (Fig. 5A; Fig. S5A). At baseline, Akata cells harboring the pCEP4 episomal vector with the pBMRF1-GFP-P2A-LNGFR-SBP cassette expressed neither GFP nor LNGFR-SBP. However, following anti-IgG stimulation and magnetic bead selection, we obtained an 87% enriched population of GFP/LNGFR^+^ cells (Fig. S5B and C). ChIP PCR for H2AK119Ub, H3K27me3, H3K4me3 (a mark of active transcription), and total histones H2A and H3 was performed in the presence of aciclovir to block lytic viral DNA replication. We found reduced H2AK119Ub occupancy at pBZLF1 and pBRLF1 in the EBV lytic cell population, with similar changes seen with H3K27me3 (Fig. 5B and C). Total H2A and H3 occupancy of lytic genes was also reduced (Fig. S5E and F), as reported for Raji cells (5). Consistent with this de-repression, we observed increased H3K4me3 occupancy of pBZLF1 and pBRLF1 (Fig. 5D), indicating active transcription of lytic EBV genes.

To our surprise, H2AK119Ub was not only lost from EBV viral genes but was also depleted from the endogenous human HOXA10 locus in EBV lytic cells (Fig. 5B). This H2AK119Ub loss from a host gene was unanticipated and suggested that the loss of H2AK119Ub upon viral reactivation was not necessarily restricted to viral genes but might be more widespread. Indeed, we confirmed the loss of total cellular H2AK119Ub by immunoblot (Fig. 5E), which showed a global reduction of H2AK119Ub in the lytic cell population, compared with minimal changes to H3K27me3 and total H3 (Fig. 5E). These findings were further confirmed using flow cytometry to detect H2AK119Ub in permeabilized cells (Fig. 5F). This showed that loss of H2AK119Ub in the BZLF1^+^ cells was visible at 12 h post-BCR crosslinking, and by 48 h, the EBV reactivating cells (BZLF1^+^ population) had completely lost all cellular H2AK119 ubiquitin expression (Fig. 5F). Interestingly, this meant that loss of H2AK119Ub appeared to occur more rapidly in the lytic cells isolated using AFMACS, where loss of H2AK119Ub was significant by 12 h (Fig. 5E). We suggest that AFMACS selection of cells that entered the lytic cycle earliest is the most likely explanation for this discrepancy. No change in H2AK119Ub expression in the non-activated (BZLF1^-^) cell population was observed by either immunoblot or flow cytometry (Fig. 5E and F), nor was there a widespread loss of H3K27me3 in the lytic cell population (Fig. S5H). Selective loss of H2AK119Ub from lytic cells could not be attributed to B-cell activation alone, as the entire cell population showed evidence of B-cell activation (phosphorylation of Akt) after addition of anti-IgG (Fig. S5D). Together, these findings suggested an active cellular H2AK119 de-ubiquitination driven by EBV lytic cycle reactivation. Whether this process was caused by viral or cellular effects was unclear.

### The USP17 de-ubiquitinating enzyme is upregulated upon EBV reactivation in a virus-dependent manner

**Fig 6:**
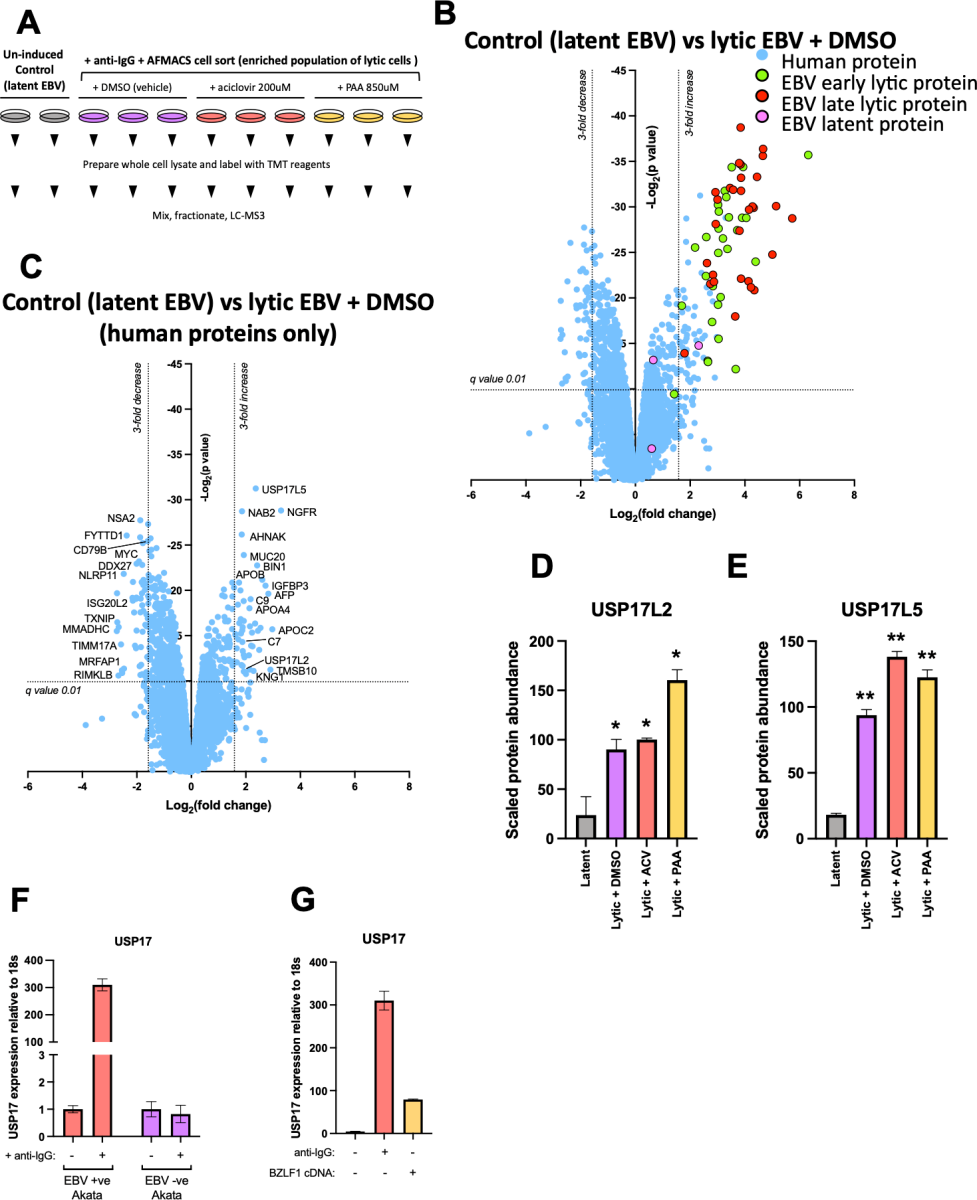
USP17 de-ubiquitinating enzyme is upregulated upon EBV reactivation in a virus-dependent manner. (**A**) Schematic overview of the quantitative proteomics analysis of the cells with latent versus lytic EBV infection. Akata cells harboring latent EBV and pCEP4 episomal vector with the pBMRF1-GFP-P2A-LNGFR-SBP cassette were stimulated with anti-IgG antibody for 24 hrs in the presence of either DMSO (vehicle), aciclovir, or PAA. Unstimulated cells were used as a control. The cells with lytic EBV (positive for both LNGFR-SBP and GFP) were then isolated using streptavidin magnetic beads, lysed, and treated with trypsin. The resulting peptides were labeled with TMT reagents, mixed, fractionated, and analyzed by mass spectrometry. (**B, C**) Scatterplots display pairwise comparisons between unstimulated (control) Akata cells with latent and lytic (anti-IgG-stimulated, DMSO-treated, AFMACS-enriched cells) EBV infection. Each point represents a single protein, plotted by its log2 (fold change in abundance) versus the statistical significance (*q* value) of that change. The *q* value was corrected for multiple hypothesis testing using the method of Benjamini-Hochberg. Dotted line: *q* = 0.01. Human and viral proteins are color-coded as indicated. Scatterplot (**C**) shows only human proteins. (**D, E**) Scaled protein abundance of USP17L2 and USP17L5 in unstimulated (control) cells (gray) and cells with lytic EBV treated with either DMSO only (purple), aciclovir (red), or PAA (yellow) (*q < 0.01, **q < 0.0001). (**F**) *USP17* expression in EBV-positive and EBV-negative Akata cells following stimulation with anti-IgG antibody. The cells were harvested 24 h after stimulation, lysed, and subjected to RT-qPCR analysis with primers specific for *USP17* and *18S*. (**G**) *USP17* expression in EBV-positive Akata cells following stimulation with anti-IgG antibody or transduction of cells with BZLF1-expressing lentivirus. The analysis was performed as described for panel F.

The cellular loss of H2AK119Ub during lytic EBV infection raised the question of whether this process is driven by viral or cellular factors. We reasoned that (i) the protein(s) responsible for depletion of total H2AK119Ub is likely to be affected by EBV reactivation and (ii) its change in abundance should be detectable by quantitative proteomics. We therefore applied multiplex Tandem Mass Tag (TMT)-based proteomics to resolve changes in the proteome of Akata cells during lytic EBV infection, 24 h after BCR crosslinking (Fig. 6A). The same Akata cell line harboring the pCEP4 vector with the pBMRF1-GFP-P2A-LNGFR-SBP cassette used for the ChIP-PCR experiments (Fig. 5) was again used to isolate a population of cells with lytic EBV. In addition, cells with lytic EBV reactivated in the presence of either aciclovir or PAA were included, as these inhibitors expedited H2AK119Ub removal, which could now be seen at 24 h post-stimulation (Fig. S5I). Unselected and unstimulated EBV-positive Akata cells were included as a negative control (Fig. 6A). We quantified a total of 7,776 cellular and viral proteins. The comparison of unstimulated (latent) and stimulated (lytic) EBV-infected cells showed a significant (*q* < 0.01) greater than 3-fold decrease in the abundance of 55 cellular proteins (Fig. 6B), including the B-cell receptor CD79 and transcriptional regulator MYC, both previously shown to be downregulated by lytic EBV infection (43). We also observed significant (*q* < 0.01) upregulation of 50 cellular proteins, including previously reported complement factor C7, alpha-fetoprotein (AFP), and kininogen (KNG1) (43), as well as 58 EBV lytic proteins. The addition of aciclovir or PAA resulted in a selective reduction of late viral gene products (Fig. S6A through E), as would be predicted. A complete list of the human and viral proteins significantly (*q* < 0.01) upregulated in the cells with lytic EBV infection is provided in Table S3, and downregulated proteins are listed in Table S4. Importantly, none of the 18 Polycomb complex-associated protein components detected showed a change in abundance in the EBV lytic cell population (Fig. S6F). Loss of Polycomb activity through depletion of the complex is therefore unlikely to be responsible for the profound decrease in cellular H2AK119Ub. Of particular interest among the significantly upregulated cellular proteins in the population of cells with lytic EBV were two members of the large USP17-like family of de-ubiquitinase (DUB) enzymes, USP17L2 and USP17L5 (Fig. 6C). Expression of both these proteins was further increased in the lytic cells treated with either aciclovir or PAA (Fig. 6D and E), and no other DUBs showed increased expression upon EBV lytic reactivation (Fig. S6G). H2AK119Ub is not known to be de-ubiquitinated by the USP17-like protein family, but because USP17-like proteins are chromatin-associated and can de-ubiquitinate histone H2AX as part of the DNA damage response (44), we considered these proteins as strong candidates for further investigation.

The USP17-like family consists of at least 32 genes with >92% DNA sequence homology (45) and is distributed across two RS447 megasatellite regions on two chromosomes (4 and 8) (46). All USP17-like proteins share 98% amino acid sequence similarity, making reliable differentiation by mass spectrometry impossible. The observed upregulation of USP17L2 and USP17L5 was likely to represent a general upregulation of the entire family. Hereafter, the USP17-like family will be referred to collectively as USP17. To investigate whether USP17 upregulation during EBV lytic cycle reactivation was transcriptional, we performed RT-qPCR with primers targeting a common region of the entire USP17-like family. This showed a marked increase in USP17 RNA expression (300-fold) in lytic EBV-positive Akata cells following anti-IgG stimulation (Fig. 6F). Importantly, there was no change in USP17 expression in the EBV-negative Akata control cells stimulated under the same conditions of anti-IgG stimulation (Fig. 6F; Fig. S5D and E). USP17 mRNA expression was also increased (>70-fold) in EBV-positive Akata cells when the lytic cycle was triggered by overexpressed BZLF1 protein alone, rather than anti-IgG (Fig. 6G). To determine whether lytic cycle-induced USP17 upregulation is a common feature of human gamma-herpesviruses, we investigated whether USP17 is upregulated upon KSHV lytic reactivation. The iSLK.219 cell line harbors latent KSHV genomes (47), and addition of doxycycline induces transcription of the RTA transgene, which stimulates KSHV lytic reactivation. USP17 mRNA was also triggered under these conditions, suggesting that, like EBV, KSHV lytic reactivation upregulates USP17 expression (Fig. S6K). Together, these data show that USP17 expression is induced in the lytic phase of both EBV and KSHV. In the case of EBV, this induction is dependent on virus reactivation and not simply a consequence of B-cell activation. Consistent with the proteomic data, aciclovir did not block the USP17 upregulation observed in Akata cells with lytic EBV (Fig. S6H through J), implying that an early lytic viral gene product was likely responsible for this phenomenon.

**Fig 7:**
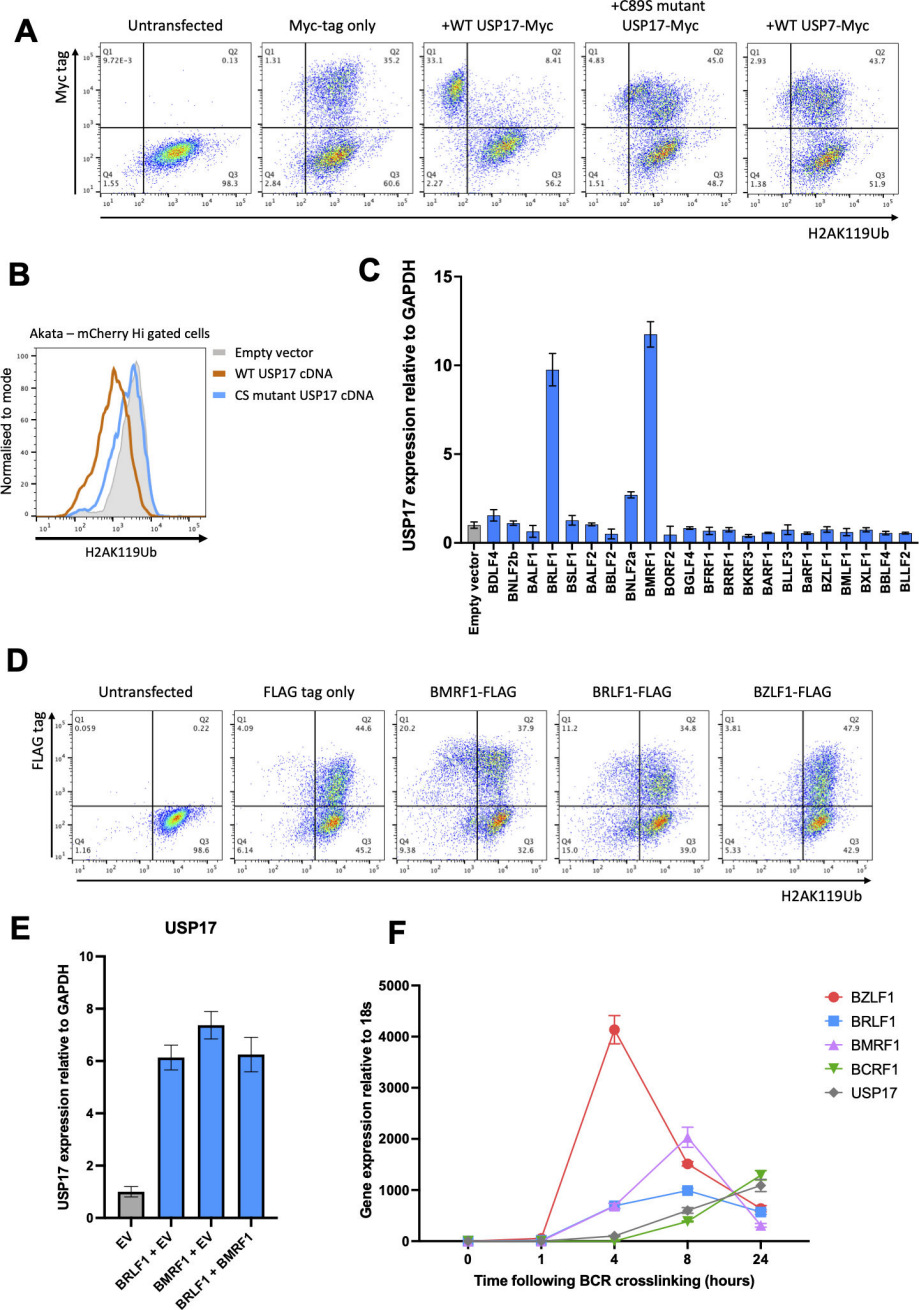
USP17 overexpression is associated with removal of H2AK119Ub. (**A**) Flow cytometry analysis of H2AK119Ub expression in cells transfected with USP17 wt and mutant constructs. HEK293T cells were transfected with vectors expressing Myc-tagged wild-type USP17, USP17 C89S mutant, or control USP7 proteins, harvested 48 hours later, permeabilized, and stained with the antibodies specific for H2AK119Ub and Myc-tag. Cells transfected with a vector expressing Myc-tag only and untransfected cells were used as controls. (**B**) Flow cytometry analysis of EBV-positive Akata cells transduced with lentiviral vector containing either wild-type USP17-P2A-mCherry or an inactive mutant USP17-P2A-mCherry. Shown are cells stained for H2AK119Ub with mCherry-Hi gating. (**C**) *USP17* expression following transfection of immediate-early and early lytic EBV ORFs. HEK293T cells were transfected with the vectors expressing indicated viral ORFs using lipofection, harvested 48 h later, lysed, and subjected to RT-qPCR analysis with primers specific for *USP17* and *GAPDH*. (**D**) Flow cytometry analysis of H2AK119Ub expression in cells transfected with EBV ORFs. HEK293T cells were transfected with vectors expressing FLAG-tagged EBV BMRF1, BRLF1, and BZLF1 ORFs, harvested 48 h later, permeabilized, and stained with the antibodies specific for H2AK119Ub and FLAG-tag. Cells transfected with a vector expressing FLAG-tag only and untransfected cells were used as controls. (**E**) *USP17* expression in cells co-transfected with the indicated viral ORFs. HEK293T cells were transfected using lipofection, harvested 48 h later, lysed, and subjected to RT-qPCR analysis with primers specific for *USP17* and *GAPDH*. Cells transfected with empty vector (EV) were used as a control. (**F**) Temporal kinetics of *USP17* and viral genes expression upon EBV reactivation. EBV-positive Akata cells were stimulated with anti-IgG antibody, harvested at indicated timepoints, lysed, and subjected to RT-qPCR analysis with primers specific for *EBV BZLF1*, *BRLF1*, *BMRF1*, *BCRF1*, *cellular USP17*, and *18S*.

To identify the potential viral protein(s) responsible, we used a set of 22 FLAG-tagged EBV ORFs from a previously published EBV gene library (48–50). Of the 22 selected ORFs, 19 had immediate-early or early lytic expression kinetics, and the expression kinetics were unclear for the remaining 3 ORFs. We overexpressed the selected viral ORFs in HEK 293T cells and analyzed USP17 expression. RT-qPCR analysis showed that two EBV gene products, the transactivator protein BRLF1 and DNA-processivity factor BMRF1, induced USP17 mRNA expression (Fig. 7C). A complementary flow cytometry analysis of HEK 293T cells overexpressing both proteins confirmed a modest reduction in H2AK119Ub (Fig. 7D). Co-expression of BRLF1 and BMRF1 did not have a synergistic effect on USP17 expression or de-ubiquitination of H2AK119Ub (Fig. 7E). Analysis of USP17 and viral gene expression in Akata cells following EBV reactivation confirmed that mRNAs of both BMRF1 and BRLF1 were expressed prior to the upregulation of USP17 (Fig. 7F). Taken together, these results suggest that USP17 is likely required for the removal of H2AK119Ub during EBV lytic reactivation, and that transcriptional upregulation of USP17 is controlled by the viral proteins BRLF1 and BMRF1 in a non-redundant manner.

### USP17 overexpression is associated with removal of H2AK119Ub

We next set out to determine whether the increased USP17 expression was responsible for the de-ubiquitination of H2AK119Ub. Unfortunately, the USP17 antibody used in previous USP17 publications (51–53) was no longer available, and despite extensive testing of commercially available USP17-specific antibodies, we were unable to identify any suitable alternative. We therefore overexpressed Myc-tagged wild-type USP17, its catalytically inactive C89S mutant (54), and the USP7 DUB as a control in HEK 293T cells, and examined intracellular H2AK119Ub expression by flow cytometry. Overexpression of wild-type USP17, but not the USP17 C89S mutant or USP7, resulted in a large reduction in H2AK119Ub expression (Fig. 7A). Furthermore, reduced H2AK119Ub expression was also seen in EBV-positive Akata cells overexpressing wild-type USP17, but not the USP17 C89S mutant (Fig. 7B). Taken together, these experiments suggest that USP17 is able to de-ubiquitinate H2AK119Ub, and the increased USP17 expression seen following lytic EBV reactivation may therefore be responsible for the profound loss of cellular H2AK119Ub.

Finally, we wanted to formally prove that the marked increase in USP17 expression was responsible for the EBV-induced loss of H2AK119Ub, but this experiment required an effective deletion/depletion of USP17. This was technically challenging because: (i) although resting cellular levels of USP17 were effectively depleted by USP17-specific shRNAs, the very large (>100×) EBV-induced induction of USP17 overcame all attempts to effectively deplete USP17 despite the use of multiple shRNAs, and (ii) the complexity of the USP17 locus with multiple gene copies made it impossible to use CRISPR-Cas9 technology to either delete or deplete the USP17-expressing genes. Therefore, despite many attempts at decreasing USP17 expression with USP17-specific shRNAs and sgRNAs both individually and in combination, we were unable to prevent a significant induction of USP17 following EBV reactivation (data not shown). It therefore remains unproven whether USP17 is indeed responsible for the loss of viral and cellular H2AK119Ub, or whether USP17 depletion can limit or prevent EBV lytic reactivation.

## DISCUSSION

In this study, we developed a plasmid-based model of EBV BZLF1 promoter silencing and used it for proteomics of isolated chromatin segments (PICh) to identify cellular regulators of EBV latency. We found that the BZLF1 promoter is repressed by the nucleosome remodeling factor CHD4 and components of the PRC1 complex. We show that CHD4 represses spontaneous lytic EBV reactivation and that PRC1 occupies viral lytic gene promoters in a PRC2-independent manner. Furthermore, we found that the PRC1 histone mark H2AK119Ub is depleted from both viral and cellular loci during EBV lytic reactivation. Finally, we show that lytic EBV induces expression of the USP17 de-ubiquitinating enzyme and that overexpression of USP17 is associated with H2AK119Ub mark removal.

The role of cellular chromatin proteins in maintaining EBV latency has been intensely studied for several decades, and many important repressors have been identified through mutation of promoter sequences, candidate gene approaches, and, most recently, forward genetic screens (12, 15–17, 55–58). To our knowledge, this is the first study to take a locus-specific proteomics approach to interrogate the chromatin occupying a silenced EBV promoter and indeed the first use of locus-specific proteomics to target episomal DNA.

The success of a PICh experiment can be determined by whether factors known to associate with the target locus are identified, as well as by the identification of novel proteins (26). The results of our pBZLF1 PICh experiment fulfilled these criteria because, in addition to identification of novel pBZLF1-associated proteins, PRC1 components, and CHD4, we also identified several proteins previously reported to bind pBZLF1. These include PRC2 components, TRIM28, and HDAC2 (4–6, 9, 34, 59). It is also important to note that several proteins known to bind pBZLF1, e.g., ZEB1/2, MEF2D, and JDP2 (12, 15, 30), were not identified in our PICh experiment. A potential explanation for this is differences in protein abundance, as upstream chromatin modifiers, such as PRC2, may be more abundant at a cellular level than transcription factors. In addition, histone modifiers may occupy multiple histones associated with pBZLF1 and therefore be present at a higher density than sequence-specific binding proteins which bind pBZLF1 at a single motif. As the PICh experiment was performed in HEK 293T cells, it is also possible that known EBV repressors were not detected due to lower abundance in a non-B-cell line.

We found that CHD4 represses both pBZLF1 expression and spontaneous EBV lytic reactivation. CHD4 is a chromatin-remodeling enzyme and a component of two distinct gene repression complexes, NuRD (60–62) and ChAHP (63). To our knowledge, the information on the role of CHD4 in regulation of herpesvirus gene expression has been so far limited to studies on KSHV in which CHD4 was identified as (i) a repressor of viral gene expression in *de novo* infection (64) and (ii) a key regulator of viral latency-lytic switch (65). Our results, therefore, extend these observations to the regulation of lytic reactivation of another gamma-herpesvirus, EBV.

How CHD4 represses spontaneous EBV lytic reactivation, and whether it acts alone or as part of the NuRD or ChAHP complexes, remains unclear. NuRD consists of the active enzymes CHD3/4/5 and HDAC1/2, which compact nucleosomes and deacetylate histones, alongside scaffold proteins GATAD2A/B, histone chaperones RBBP4/7, histone-binding proteins MTA1-3, and CpG island-specific binders MDB2/3 (66). NuRD recruitment to genomic loci is often guided by the binding of MTA proteins to transcription factors (67) or MBD proteins to CpG methylated DNA (68). We found that knockdown of MTA and MBD proteins in Akata cells did not lead to spontaneous BZLF1 expression, suggesting that CHD4 recruitment may not be dependent on these proteins. We also found that knockdowns of HDAC1 and 2 did not provoke spontaneous EBV reactivation, which was a surprise as HDAC2 is a recognized repressor of pBZLF1 (34). Three independent sgRNAs were designed to target each gene in our validation experiments; thus, failure to achieve at least one effective knockdown is unlikely. A possible explanation for the lack of phenotype is redundancy between HDAC1/2 (69), meaning that both enzymes would need to be depleted for the effect to be observed. Previous studies that established a role for HDAC enzymes in EBV latency used HDAC inhibitors, such as Trichostatin A, which inhibit both HDAC1 and 2 simultaneously (4). In KSHV *de novo* infection, MBD3 and GATAD2A were identified as repressors of viral gene expression along with CHD4, and it was suggested that MBD3 may target NuRD to CpG islands in the KSHV genome (64). However, as pBZLF1 does not contain a CpG island (5), this is unlikely to account for the recruitment to EBV. CHD4-mediated repression of lytic KSHV lytic reactivation has also been shown to occur through the interaction of CHD4 with latent viral protein LANA and a component of cellular ChAHP complex, ADNP (65, 70). Finally, CHD4 can also be recruited to its loci independently by other means, including direct binding to H3K9me3 (71).

The role of Polycomb PRC2 in the maintenance of EBV latency is well described (4–6), but our study suggests that PRC1 is an independent repressor of lytic reactivation. PRC1 occupies the LMP1 and 2 promoters in Burkitt lymphoma cell lines with a latency I phenotype (72), and therefore could also target lytic gene promoters. Whether PRC1 is recruited to multiple regions of the EBV genome by the same mechanism is uncertain, as we found that occupancy of pBZLF1 by H2AK119Ub was dependent on both canonical (PRC2-dependent) and non-canonical (PRC2-independent) pathways. The PRC1 co-factor KDM2B has been shown to bind pBZLF1 (73), which could explain PRC1 recruitment via the non-canonical pathway.

Loss of H2AK119Ub from pBZLF1 and pBRLF1 during lytic reactivation provided further evidence that PRC1 acts as a repressor of lytic reactivation. Most surprising was the complete loss of cellular H2AK119Ub following lytic reactivation; unlike PRC2, the loss was not restricted to viral genes. This global cellular loss of H2AK119Ub may represent “spill-over” of the mechanism used to deubiquitinate the viral genome. The loss of H2AK119Ub in Akata cells was visible by flow cytometry from around 12 h after the onset of lytic reactivation. It would, therefore, seem unlikely that loss of H2AK119Ub from the viral genome is necessary for the lytic switch to occur, but it could be required for a later event, such as viral genome replication.

To investigate changes in histone modifications during EBV reactivation, we required a pure population of cells containing lytic virus. We used the AFMACS system for this purpose as it enabled cell sorting at an early time point during the lytic cycle. An alternative method would be to isolate cells using an antibody to the EBV lytic protein GP350, which is expressed at the cell surface during reactivation (17, 43). While the latter method does not require generation of a cell line expressing the AFMACS plasmid, GP350 expression is blocked by aciclovir and is therefore not compatible with ChIP PCR, where aciclovir is used to prevent viral genome amplification.

De-ubiquitination of multiple cellular proteins, including H2A, has been described in Akata cells containing lytic EBV and was attributed to B-cell activation, rather than to the virus itself, as similar changes were reported in EBV-negative Akata cells following BCR crosslinking (74). In contrast, our experiments clearly demonstrated that loss of H2AK119Ub only occurred in Akata cells expressing BZLF1 after BCR crosslinking even though the entire cell population showed evidence of B-cell activation. A focus for future work will be to determine whether H2AK119Ub loss occurs in other cell types containing lytic EBV if reactivation is triggered by a BCR-independent mechanism, such as BZLF1 overexpression.

The loss of cellular H2AK119Ub upon lytic reactivation led to a search for the de-ubiquitinase(s) (DUBs) responsible for this phenotype. We found that expression of the USP17 DUB was markedly increased on both mRNA (up to 300-fold) and protein (more than 3-fold) level during the lytic cycle, and we provide three lines of evidence that this protein is likely to be responsible for H2AK119Ub de-ubiquitination: (i) our unbiased proteomic analysis showed that USP17 was the only human DUB to be significantly upregulated in lytic Akata cells, (ii) upregulation of USP17 could be induced by overexpression of two lytic viral proteins, and (iii) overexpression of USP17 resulted in de-ubiquitination of H2AK119Ub. Upregulation of cellular genes during EBV lytic cycle reactivation is restricted due to virus-induced shutoff of host gene expression, mediated by canonical (non-specific destruction of mRNA by the viral exonuclease BGLF5) or non-canonical (BGLF5-independent) mechanisms (75–77). The marked increase in USP17 expression might therefore represent an attractive phenomenon for investigating how host cell genes evade these lytic EBV-induced shutoff mechanisms.

A previous proteomic analysis of expression changes in lytic Akata cells did not detect any USP17 peptides (43). As the peptides identified in our data set are common to multiple USP17-like family members, they may have been inadvertently excluded as being unattributable to a single protein due to the unusual nature of the USP17 gene family as discussed below.

An outstanding question in this study is whether the increased expression of USP17 is indeed required for EBV lytic reactivation and replication. To address this issue definitively, effective deletion or depletion of USP17 family members is necessary. This proved to be very challenging due to the marked extent of USP17 upregulation during the lytic cycle. Multiple attempts, including CRISPR-Cas9-mediated deletion with sgRNAs, CRISPR-dCas9-mediated depletions, and use of multiple shRNAs covering the majority of USP17 gene family members, have so far been unable to reduce the upregulation of USP17 during lytic EBV reactivation sufficiently to observe a phenotype. This is due to the complex and unusual genetic profile of the USP17 gene family. This gene family resides within the RS447 DNA megasatellites on chromosomes 4 and 8 (46, 78–80) and consists of tandem repeat sequences present at a high and somewhat variable copy number, ranging from 23 to 103 gene family members, with substantial inter-individual variation (46). At least 32 USP17-like gene ORFs are spread over blocks of RS447 repeats, and these ORFs are present on two chromosomes with at least 23 ORFs on chromosome 4 and at least 9 ORFs on chromosome 8 (81). Furthermore, each of the 32 USP17-like genes may themselves have multiple copies with significant variation between individuals (82). While our manuscript was in preparation, a cellular protein DUX4 was shown to play a critical role for lytic replication of several herpesviruses, including EBV-related gamma-herpesvirus KSHV (83). Curiously, DUX4 shares common features with USP17: (i) both DUX4 and USP17 are present in multiple copies in RS447 repeats (84, 85); (ii) like USP17, DUX4 is markedly upregulated in lytic KSHV infection (83); and (iii) the expression of both genes is regulated by cellular ZEB1 repressor protein (86). Further studies are necessary to elucidate whether increased USP17 expression is required for lytic gamma-herpesvirus replication and, if this is the case, also to understand why viral latency-to-lytic switch relies on the cellular genes present in high copy numbers within DNA megasatellites.

We identified inducible expression of USP17 triggered by EBV lytic proteins BRLF1 and BMRF1. Cytokine-induced expression of USP17 in response to IL-4 and IL-6 is well described (80), which suggests that EBV may be hijacking an existing cellular pathway. Overexpression of BRLF1 and BMRF1 each independently induced expression of USP17 mRNA in HEK 293T cells, although the degree of USP17 upregulation was approximately 30-fold lower than that following EBV reactivation in Akata cells, possibly reflecting differences in cell type or the state of cellular activation. USP17 has a variety of known de-ubiquitination targets including CDC25A (54) and ELK-1 (87). While H2AK119Ub is not a recognized target, USP17 does deubiquitinate the closely related H2AX as a negative regulator of the DNA damage response (44). Of note, overexpression of USP17 has been observed to result in non-specific de-ubiquitination of cellular proteins, due to the highly active nature of the enzyme (88). Further experiments are therefore required to demonstrate specific interaction of USP17 with H2AK119Ub as well as to clarify why de-ubiquitination of H2A is beneficial for the lytic virus.

In summary, we identified the chromatin-remodeling enzyme CHD4 and components of the PRC1 complex as critical regulators of the latent-lytic EBV switch and showed that, during lytic reactivation, EBV depletes the PRC1 histone mark (H2AK119Ub) and induces profound upregulation of de-ubiquitinating enzyme USP17. Overexpressed USP17 de-ubiquitinates H2AK119Ub in co-transfection experiments, suggesting that it is likely responsible for H2AK119Ub depletion during lytic EBV infection. Our findings provide important insights into the mechanisms of latency maintenance and lytic reactivation of oncogenic herpesviruses and reveal novel potential targets for therapeutic interventions.

### Limitations of the study

As the PICh method requires a highly abundant target locus, we needed to use an episomal model system of BZLF1 promoter silencing in which the episomes could be maintained at higher copy number than wild-type EBV episomes. The use of HEK 293T cells was therefore a compromise, but as these cells are able to support latent EBV infection and reactivation (89), the silencing mechanisms involved are likely to be similar. The use of EBNA1-OriP to maintain the model system episomes was important to ensure that nuclear location and association with the human genome were representative of latent EBV infection. A previous study using Hi-C sequencing found that plasmids maintained as episomes by the EBNA1-OriP interaction have the same characteristic preference for association with gene-poor, A-T-rich areas of the human genome as wild-type EBV (90). Nonetheless, we cannot be certain that the PICh experiment identified cellular proteins, which do not normally bind pBZLF1 in the native EBV genome. For example, we identified the MCM complex and TERF2, OriP-interacting factors which may have been present due to the close proximity of pBZLF1 and OriP in the model episomes. Likewise, the contribution of EBNA1 to pBZLF1 silencing in the model episomes is uncertain, as we did not determine whether PRC1 and CHD4 occupied pBZLF1 in the absence of EBNA1. Depletion of EBNA1 has previously been shown to provoke spontaneous reactivation of EBV (91), implying a role in maintenance of latency, presumably through an indirect mechanism, as EBNA1 does not bind directly to pBZLF1 (92).

In this study, we identify CHD4 as an essential factor for the maintenance of EBV latency, based on the observation of spontaneous lytic reactivation following CHD4 depletion in Akata cells. Although direct binding of CHD4 to pBZLF1 was strongly suggested by PICh experiments in HEK293T cells, due to technical limitations, CHD4 genome occupancy could not be independently confirmed by CHIP-seq or CUT&RUN in B-cells. We therefore cannot exclude the possibility that CHD4 maintains EBV latency in B-cells through an indirect mechanism.

## MATERIALS AND METHODS

### Cell lines and tissue culture

HEK 293T cells (Lehner Laboratory stock) were maintained in Dulbecco’s Modified Eagle Medium (DMEM, Sigma-Aldrich) plus 10% fetal calf serum (FCS, Gibco) and 100 U/mL penicillin/streptomycin (Gibco). Akata and EBV-negative Akata Burkitt lymphoma cell lines were a kind gift from Dr. Andrew Bell, University of Birmingham. Both were maintained in RPMI 1640 medium (Sigma-Aldrich) plus 10% FCS and penicillin/streptomycin. Akata cells were stimulated to enter the lytic cycle by crosslinking the B-cell receptor using a goat-anti-human IgG antibody (Cappel, MPBio) at a concentration of between 10 and 100 µg/mL. The antibody was added to the media for 6 h, then removed by washing twice in phosphate-buffered saline (PBS) and resuspending in fresh media. Subsequent analysis was performed at the time points indicated in individual experiments, most commonly at 24 h. The iSLK.219 endothelial cell line, which harbors latent KHSV and a doxycycline-inducible RTA transgene, was a kind gift from Dr. Frank Neipel, University Hospital Erlangen. The cells were maintained in DMEM plus 10% FCS, 1% glutamate (Sigma-Aldrich), and penicillin/streptomycin. To induce KSHV lytic reactivation, doxycycline was added at 1 µg/mL for 24 h. All cell lines were incubated at 37°C with 5% CO_2_.

### Antibodies

The following primary antibodies were used: goat α–PCGF2 (Abcam ab5267, used for immunoblot), rabbit α–PCGF6 (ProteinTech 24103-1-AP, used for immunoblot), rabbit α-RING1A (Abcam ab32644, used for immunoblot), rabbit α-RING1B (Cell Signaling #5694, used for immunoblot), rabbit α-BCOR (ProteinTech 12107-1-AP, used for immunoblot), rabbit α-EZH2 (Thermo Fisher Scientific 36-6300, used for immunoblot), rabbit α-CHD4 (Abcam ab72418, used for immunoblot), mouse α-BZLF1 (Santa Cruz Biotechnology sc-53904, used for immunoblot and FACS), mouse α-BMRF1 (Santa Cruz Biotechnology sc-58121, used for immunoblot), mouse α-calnexin (AF8, kind gift from M. Brenner, Harvard Medical School, used for immunoblot), mouse α-β-actin (Sigma-Aldrich A2228, used for immunoblot), rabbit α-VCP (ProteinTech 10736-1-AP, used for immunoblot), rabbit α-H2AK119Ub (Cell Signaling #8240, used for immunoblot, ChIP-PCR, and FACS), rabbit α-H3K27me3 (Cell Signaling #9733, used for immunoblot, ChIP-PCR, and FACS), rabbit α-H3K4me3 (Cell Signaling #9751, used for immunoblot, ChIP, and FACS), rabbit α-H3K9me3 (Abcam ab8898, used for immunoblot and FACS), rabbit α-Histone H2A (Cell Signaling #12349, used for ChIP), rabbit α-Histone H3 (Abcam ab1791, used for immunoblot and ChIP), rabbit α-phospho-Akt (pAkt) (Cell Signaling #4060, used for FACS), normal rabbit IgG (Cell Signaling #2729, used for ChIP), mouse α-Myc-tag (Cell Signaling #2276, used for FACS), and mouse α-FLAG tag (Invitrogen, MA1-91878, used for FACS). HRP-conjugated secondary antibodies for immunoblot were obtained from Jackson ImmunoResearch. Alexa Fluor-conjugated secondary antibodies for FACS were obtained from Thermo Fisher Scientific.

### Plasmid construction

The pBZLF1-GFPx5 reporter was generated by cloning the pBZLF1 promoter and GFP coding sequence into the pCEP4 vector (Thermo Fisher Scientific). The pBZLF1 promoter sequence was amplified from an Akata cell DNA extract using PCR with primers TTTGGACGAACTGACCACAA-3′ (forward) and 5′-CTTCAGCAAAGATAGCAAAGGTG-3′ (reverse). pBZLF1 was multimerized to five copies using the PCR primers above, with the addition of restriction sites for *BsrGI* (TGTACA) on the forward primer and *BsiWI* (CGTACG) on the reverse primer. The pCEP4-pBZLF1x1 plasmid was digested with *BsrGI* (New England Biosciences), and the PCR product was ligated in, resulting in a new plasmid with two sequential BZLF1 promoters and a destroyed *BsrGI/BsiWI* hybrid restriction site between them. The process was repeated until five sequential copies of pBZLF1 were present, which was confirmed by digestion of pBZLF1x5 GFP en bloc, followed by gel electrophoresis. The pCEP4-Cas9 vector was generated by cloning the Cas9 sequence from lentiCas9-Blast (Addgene #52962, kindly deposited by Dr. Feng Zhang) into the pCEP4 vector using Gibson assembly. The pCEP4-pBMRF1-GFP-P2A-LNGFR-SBP plasmid was generated by cloning the BMRF1 promoter and GFP-P2A-LNGFR construct (41) into the pCEP4 vector using Gibson assembly. The 549 bp BMRF1 promoter was amplified by PCR from Akata DNA using the primers 5′-CTGCTGATTGAAGGCATCTT-3′ (forward) and 5′-GATCACAAGCAGCAGCAGAAG-3′ (reverse).

### Transfection, electroporation, and lentivirus production

HEK 293T cells were transfected using lipofection. For a standard experiment, cells were seeded at 60–80% confluency into a 6-well plate and transfected with 3 µg of plasmid DNA using TransIT-293 (Mirus), according to the manufacturer’s instructions. Selection with 50 µg/mL hygromycin was performed for 48 h. Lentivirus was produced by triple-transfection of HEK 293T cells with a lentiviral vector alongside packaging vectors pCMV∆R8.91 and pMD.G. For a standard transfection, cells were seeded into a 6-well plate as described above and transfected with a total of 3 µg plasmid DNA (comprising 1.5 µg of lentiviral vector, 1 µg of pCMV∆R8.91, and 0.5 µg pMD.G) using TransIT-293. Supernatant containing lentivirus was collected at 48 h and filtered through a 0.45 µm filter (Sartorius Stedim Biotech). The lentivirus was applied to target cells and centrifuged at 1,800 rpm for 45 min. Expression of transgenes was assessed for 48 h post-transduction.

Akata cells were transfected using a Neon electroporator (Thermo Fisher Scientific) according to the manufacturer’s instructions. For a typical transfection, 2 × 10^6^ cells were washed in PBS and resuspended in 150 µL of buffer R with 10 µg of plasmid DNA. Electroporation was performed with 1 pulse of 1,300 V for 30 ms, after which cells were resuspended in warmed RPMI with 10% fetal calf serum. Selection with 50 µg/mL hygromycin was performed for 48 h.

### shRNA-mediated knockdown of human genes

The pHR-SIREN vector (a generous gift from Greg Towers) was used for lentiviral expression of shRNAs, with co-expression of a puromycin-resistant gene. Hairpin oligonucleotides were selected from the Broad Institute shRNA library, and oligonucleotides were purchased from Sigma-Aldrich, then annealed and ligated into the pHR-SIREN vector digested with BamHI and EcoRI (NEB). Successful cloning was confirmed by Sanger sequencing. The forward shRNA oligonucleotides were as follows: shScrambled 5′-GAT CCG TTA TAG GCT CGC AAA AGG TTC AAG AGA CCT TTT GCG AGC CTA TAA CTT TTT TG-3′, shRING1A 5′-GAT CCG CCC TGA TCT CTA AGA TCT ATT TCA AGA GAA TAG ATC TTA GAG ATC AGG GCT TTT TTG-3′, shEZH2 5′-GAT CCG CGG CTC CTC TAA CCA TGT TTA TTC AAG AGA TAA ACA TGG TTA GAG GAG CCG TTT TTTG-3′, shCHD4 5′-GAT CCG CCT GCG GAA TGA TAA AGA TAA TTC AAG AGA TTA TCT TTA TCA TTC CGC AGG TTT TTT G-3′, shHDAC2 5′-GAT CCG ACG GTA TCA TTC CAT AAA TAT TCA AGA GAT ATT TAT GGA ATG ATA CCG TCT TTT TTG-3′*.*

### CRISPR-Cas9-mediated knockdown of human genes

The sgRNAs were designed using the Broad Institute GPP sgRNA Designer Tool, and oligonucleotides were purchased from Sigma-Aldrich. The sgRNA sequences are listed in Table S5. The oligonucleotides were annealed and cloned into the pKLV-U6-sgRNA-PGK-Puro-2A-BFP backbone vector (Addgene #50946, kindly deposited by Dr. Kosuke Yusa), which was digested with *BbsI*.

### Human cDNA and EBV lytic gene ORF overexpression

Myc-tagged wild-type USP17 and CS-mutant USP17 cDNAs were a kind gift from Dr. Peter Shaw, University of Nottingham. The myc-tagged USP7 cDNA was a kind gift from Dr. Roger Everett, University of Glasgow. The cDNAs were overexpressed in HEK 293T cells using lipofection with TransIT-293, as described above. For lentiviral expression of the USP17 cDNAs in Akata cells, the pHRSIN-pSFFV-mCherry-pPGK-PuroR vector was used, and USP17 cDNAs were co-expressed with the mCherry fluorophore using a P2A sequence. The EBV lytic gene ORFs were overexpressed in HEK 293T cells using lipofection with TransIT-293, as described above.

### Flow cytometry and intranuclear antibody staining

Cells were washed in PBS and then acquired on an LRSFortessa (BD). For intranuclear staining, approximately 1 × 10^6^ cells per condition were resuspended in PBS and fixed in 3.6% formaldehyde for 20 min, then permeabilized in 90% methanol at −20°C for 30 min. The cells were blocked in PBS with 1% BSA and 5% fetal calf serum for 15 min, and then primary antibody was added at the concentration recommended by the manufacturer for 30 min. After washing in PBS, a fluorophore-conjugated secondary antibody (typically goat-anti-mouse IgG conjugated to Alexa Fluor 647 or donkey-anti-rabbit IgG conjugated to Alexa Fluor 488 [Thermo Fisher Scientific]) was added at the concentration recommended by the manufacturer for 30 min. For all flow cytometry experiments, forward and side scatter gating was performed to exclude dead cells (or cells which had died prior to fixation).

### Purification of lytic Akata cells using antibody-free magnetic cell sorting (AFMACS)

Akata cells containing the pBMRF1-GFP-P2A-LNGFR-SBP plasmid were stimulated to enter the lytic cycle using anti-IgG at 100 µg/mL. For ChIP-PCR, 200 µM of aciclovir was also added, and the cells were incubated for 12 h. For mass spectrometry, cells were incubated for 24 h with either 200 µM of aciclovir or 100 µg/mL PAA, as indicated. Purification of lytic cells expressing cell surface LNGFR-SBP was then performed as described (41), with minor modifications. Cells were washed twice in PBS with 0.5% BSA and resuspended in ice-cold incubation buffer (Hank’s Balanced Salt Solution [HBSS; Gibco], 2% dialyzed FCS, 1× RPMI Amino Acids [Merck], 1× glutamine [Merck], 2 mM EDTA, and 10 mM HEPES). Dynabeads Biotin Binder magnetic beads (Thermo Fisher Scientific) were equilibrated in incubation buffer and added to the cells at a concentration of 100 µL beads per 1 × 10^7^ cells, then incubated at 4°C for 20 min with rotation. Beads were immobilized on a magnetic rack and washed twice with incubation buffer and then resuspended in release buffer (RPMI, 10% fetal calf serum, and 2 mM biotin [Sigma-Aldrich]) for 15 min. The eluted cells were then used for either ChIP PCR or mass spectrometry, as described below.

### Proteomics of isolated chromatin (PICh) probe design and plasmid capture assay

Eighteen 50-nucleotide 2′ fluoro-RNA probes were designed to tile a 900 bp sequence from the pCEP4-pBZLF1-GFP plasmid which centered on pBZLF1, with 300 bp of flanking DNA on either side. A nineteenth probe was also designed to cover the join between pBZLF1 sequences due to multimerization of the promoter. Each probe also contained a desthiobiotin molecule appended to the 5′ end via a long spacer arm and a DNA nucleotide “cap” at the 3′ end. The probe sequences are listed in Table S6. For the plasmid capture assay, the pBZLF1x5 GFP segment was excised from the backbone vector using *BssHII* and *NotI* (NEB). Two hundred nanograms of plasmid DNA was combined with 0.5 µM of PICh probes (an equimolar mix of all 19 probes) in LB3JD buffer (10 mM HEPES, 100 mM NaCl, 2 mM EDTA, 1 mM EGTA, 0.2% SDS, 0.1% sarkosyl, and an EDTA-free protease inhibitor cocktail tablet [Roche]). The hybridization reaction was performed in a PCR system by heating to 90°C for 2 min, cooling to 37°C at 1°C per minute, and incubating at 37°C for 30 min. Seventy-five microliters of Dynabeads MyONE C1 Streptavidin magnetic beads (Thermo Fisher Scientific) were added and incubated for 30 min at room temperature with rotation. Beads were then immobilized on a magnetic stand, and supernatant was collected as flow-through. Beads were washed five times with LB3JD buffer and then resuspended in 300 µL LB3JD and heated for 5 min at 37°C. The probe-DNA hybrids were eluted in 100 µL of LB3JD containing 12.5 mM of D-biotin (Invitrogen) by heating to 65°C for 15 min. One-tenth of the input, flow through, and eluate were analyzed by agarose gel electrophoresis with ethidium bromide staining.

### Proteomics of isolated chromatin (PICh) method

Chromatin for the PICh experiment was prepared from 4 × 10^9^ HEK 293T cells expressing the pBZLF1-GFP reporter plasmid and also from the same amount of untransfected control cells. For the transfection, ten 10 cm cell culture plates were seeded with HEK 293T cells at approximately 70% confluence, and each plate was transfected with 40 µg of pBZLF1x5 GFP reporter plasmid using TransIT-293. At 48 h post-transfection, hygromycin was added at 50 µg/mL, and selection was continued until day 7, when the cells were harvested. Cells were mobilized using trypsin and transferred to 250 mL Corning centrifuge tubes, then washed twice with PBS containing 0.1 mM PMSF. Fixation was performed using 3.5% formaldehyde in PBS for 30 min, after which the cells were washed three times in PBS with 1 mM PMSF. The cell pellet was resuspended in sucrose buffer (0.3 M sucrose; 10 mM HEPES-NaOH, pH 7.9; 1% Triton X-100; 2 mM MgOAc; 1 mM PMSF) and lysed with 15 strokes of a 40 mL Dounce homogenizer. After washing once in PBS with 0.5% Triton X-100, RNase digestion was performed overnight at 4°C with 1 mg/mL RNase A (Qiagen). The following day, the chromatin pellet was washed three times in PBS with PMSF and resuspended in MNase digest buffer (100 mM HEPES, pH 7.9; 25 mM MgCl_2_; 25 mM CaCl_2_; 35 0mM KCl) with 1000 U of MNase. The chromatin was digested with MNase at 37°C for 20 min to yield predominantly mono- and di-nucleosomal fragments, and the reaction was stopped by adding 5 mM EGTA and cooling on ice. The chromatin pellet was washed twice in ice-cold PBS with 5 mM EGTA, then resuspended in LBJD4 sonication buffer (50 mM Tris, pH 8; 200 mM NaCl; 20 mM EDTA, pH 8; 1% SDS). To solubilize the chromatin, four cycles of sonication (15 s on/45 s off) were performed using a Misonix S-4000 probe sonicator in an ice-water slurry. The chromatin was heated to 58°C for 5 min in a thermocycler and then centrifuged at 15,000 × *g* for 15 min at room temperature to pellet the insoluble fragments, which were discarded. The chromatin concentration of each sample was calculated using a NanoDrop spectrophotometer: 20 mg of chromatin was used for the pBZLF1 PICh and 10 mg for the telomere PICh. The chromatin was pre-cleared by overnight incubation at 4°C with Ultralink streptavidin-coated agarose beads (Thermo Fisher Scientific) and desalted by centrifugation through Sepharose S-400-HR (GE Healthcare) in a Pierce spin column at 750 × g for 5 min. The RNA probes were added to the pre-cleared chromatin at a concentration of 100 µM (equimolar mix of all 19 probes). Hybridization was performed in a PCR machine at 25°C for 3 min, 82°C for 5 min, and 37°C for 2 h. The probe-chromatin mix was then centrifuged at 18,000 × *g* for 15 min to pellet any further insoluble chromatin. Pulldown of the probe-DNA hybrids was performed using MyONE C1 streptavidin beads equilibrated in LBJD4 buffer. The chromatin was diluted with 1 volume of Milli-Q water in a Falcon 15 tube, and the beads were added, then incubated for 2 h at room temperature with rotation. The beads were immobilized on a magnetic rack and washed five times with 10 mL of LB3JD buffer. The beads were then transferred to a 1.5 mL microcentrifuge tube and incubated at 42°C for 5 min in 1 mL of low-salt LB3JD buffer containing 50 mM NaCl. The probe-DNA hybrids were eluted from the beads by incubation with 900 µL of LB3JD buffer containing 12.5 mM of biotin for 1 h at room temperature, followed by heating to 65°C for 15 min. The eluted protein was precipitated by adding 20% TCA and incubating on ice for 20 min, then centrifuged at 16,000 × *g* for 15 min at 4°C. The protein pellet was washed twice with acetone at −20°C, then resuspended in crosslinking reversal buffer (250 mM Tris, pH 8.8; 2% SDS; 0.1 M 2-mercaptoethanol) and heated to 99°C for 25 min. After decrosslinking, the protein was subjected to analysis by mass spectrometry as described below.

### Mass spectrometry

For the EBV reactivation proteomics, cells were lysed in 5% SDS/TEAB, pH 8.5, and digested on S-traps according to the manufacturer’s recommendations (Protify). Subsequently, samples were dried and derivatized with TMT reagents according to the manufacturer’s recommendations (Thermo Fisher Scientific). Labeled samples were pooled, cleaned up by C18 SPE, and subjected to high-pH reversed-phase fractionation, resulting in 24 fractions. Each fraction was run on a 3-hour reverse-phase gradient on a nano-LC-MS system in line with an Orbitrap Fusion mass spectrometer. For the PICh experiment, proteins were eluted from streptavidin beads and decrosslinked by heating in an SDS-containing buffer. Samples were then subjected to S-trap digestion as above. Digested samples were dried and run on a similar LC-MS setup, except with 1-hour runs per sample. All samples were searched using MASCOT (Matrix Science) within Proteome Discoverer v2.1 (Thermo Fisher Scientific) against the SwissProt Human database and a database of common contaminant proteins. Peptide FDR was controlled against a decoy database search at 1%. For TMT-labeled samples, reporter ion S/N was extracted and used for quantification. For the PICh experiment, label-free quantification based on the top three most abundant peptides for each protein was used.

### Quantitative reverse-transcription PCR (qRT-PCR)

Total RNA was extracted from cells using the RNEasy Plus Kit (Qiagen) and RNA concentration calculated using a NanoDrop spectrophotometer. One microgram of RNA was then reverse transcribed into cDNA using an oligo(dT)_20_ primer (Thermo Fisher Scientific) and SuperScript IV RT reverse transcriptase (Thermo Fisher Scientific), following the manufacturer’s instructions. The qPCR was performed on a Thermo ABI QuantStudio DX Real-Time PCR machine (Applied Biosystems) using SYBR Green Master Mix (Thermo Fisher Scientific). Primers are listed in Table S7. Standard cycling parameters were 50°C for 2 min, 95°C for 10 min, followed by 40 cycles of 95°C for 15 s and 58°C for 1 min. Results were interpreted using the delta-delta *C*_*T*_ method and presented as the mean with standard deviation of three replicates. To calculate pBZLF1x5 GFP reporter plasmid copy number, genomic DNA was extracted from HEK 293T cells expressing the reporter plasmid using a DNeasy Blood & Tissue Kit (Qiagen). DNA was quantified using a NanoDrop spectrophotometer and then diluted to 8 ng/µL (approximately 1,000 cells worth of DNA per µL). Quantitation standards with a known plasmid copy number were prepared from miniprep DNA using serial dilution. The cellular DNA and quantitation standards were then run in a qPCR reaction using the cycling parameters described above. The pCEP4 plasmid copy number per cell was calculated based on a curve generated from the quantitation standards using the equation *y = mx +* c.

### Chromatin immunoprecipitation (ChIP)

ChIP was performed as described previously (93), with minor modifications. 3 × 10^6^ cells were crosslinked in 1% formaldehyde for 10 min at room temperature with rotation, then quenched with the addition of 0.125 M glycine for 5 min. The cells were washed once with ice-cold PBS, and then cell lysis buffer was added (10 mM HEPES, 85 mM KCl, 0.5% IGEPAL, EDTA-free protease inhibitor cocktail tablet [Roche]). Nuclei were lysed in nuclei lysis buffer (50 mM Tris, pH 8.1; 10 mM EDTA; 1% SDS; EDTA-free protease inhibitor cocktail tablet), and chromatin was sheared using a Bioruptor Pico (Diagenode) to obtain chromatin fragments between 400 and 600 bp in length. The chromatin was diluted 1:10 with IP dilution buffer (20 mM Tris, pH 8.1; 2 mM EDTA; 150 mM NaCl; 1% Triton X-100; 0.01% SDS; EDTA-free protease inhibitor cocktail tablet) and pre-cleared with Pierce Protein G magnetic beads (Thermo Fisher Scientific) for 2 h at 4°C with rotation. At this point, a small aliquot was removed as the input for subsequent qPCR analysis. For immunoprecipitation, antibodies were added at the concentration recommended by the manufacturer, along with fresh Protein G magnetic beads, and incubated overnight at 4°C. The beads were immobilized on a magnetic rack and washed five times: twice with low-salt buffer (20 mM Tris, pH 8.1; 2 mM EDTA; 50 mM NaCl; 1% Triton X-100; 0.1% SDS), once with LiCl buffer (910 mM Tris, pH 8.1; 1 mM EDTA; 0.25 M LiCl; 1% IGEPAL; 1% sodium deoxycholate monohydrate), and twice with TE buffer (10 mM Tris-Cl, 1 mM EDTA, pH 8.0). The protein-DNA complexes were eluted from the beads in 1% SDS and 150 mM NaHCO_3_, and crosslinks reversed by the addition of 0.3M NaCl and RNAse A, with incubation at 67°C overnight. Proteinase K was then added and incubated for 2 h at 45°C. DNA was purified using a PCR purification kit (Qiagen). Quantification of DNA was performed by qPCR using SYBR Green Master Mix (Thermo Fisher Scientific) on a Thermo ABI QuantStudio DX Real-Time PCR machine (Applied Biosystems) using the primers listed above.

### Immunoblotting

Cells were lysed in 1% SDS in TBS with 1:100 Benzonase and heated to 70°C for 10 min. Separation was performed by SDS-PAGE, and proteins were transferred to a PVDF membrane (Millipore). Membranes were blocked in 10% Marvel milk in PBS with 0.2% Tween-20, then probed with the relevant antibodies. Bands were visualized using either ECL, West Pico, or West Dura (Thermo Fisher Scientific).

### Statistical analysis

#### PICh data analysis

Analysis of the relative protein abundances in cells with and without the silenced pBZLF1-GFP episome was conducted using Perseus (94). To identify outlier protein abundance ratios, taking into account total protein abundance, Significance B was calculated for each protein (33). Correction for multiple testing was applied using the Benjamini-Hochberg method with FDR < 5%. GOCC subcellular localization information was downloaded from Uniprot.

#### TMT-based proteomic data analysis

Statistical analysis was conducted within R using the Bioconductor package LIMMA. *P* values generated by LIMMA were corrected for multiple hypothesis testing using the Benjamini-Hochberg method (95) to generate an FDR (*q* value) for each comparison.

#### Analysis of protein-protein association networks using STRING

The significantly enriched proteins from the BZLF1 PICh experiments were analyzed using version 12 of STRING (96) with the maximum confidence interaction score and all interaction sources selected except “textmining.” The proteins were displayed with ‘kmeans’ clustering.

#### Analysis of qPCR data

Data from qPCR experiments were analyzed using GraphPad Prism 7 with two-tailed paired Student *t*-tests performed where indicated.

## Data Availability

The mass spectrometry proteomics data have been deposited to the ProteomeXchange Consortium (http://proteomecentral.proteomexchange.org) via the PRIDE partner repository (97) with the dataset identifier PXD068122. Further information and requests for resources and reagents should be directed to and will be fulfilled by the lead contact, Paul Lehner (pjl30@cam.ac.uk). All unique reagents generated in this study are available upon request.

## SUPPLEMENTAL MATERIAL

Figure S1 jvi.01408-25-s0001.tiff Repressed pBZLF1(x5)-GFP reporter is activated by BZLF1 cDNA or histone deacetylase compounds.

Figure S2 jvi.01408-25-s0002.tiff Expression of polycomb PRC1 E3 ubiquitin ligases in human cell lines (related to Fig. 2).

Figure S3 jvi.01408-25-s0003.tiff Chromatin modifications associated with episome-expressed promoters or endogenous genomic loci (related to Fig. 2).

Figure S4 jvi.01408-25-s0004.tiff PRC1 occupies viral gene promoters along with PRC2 and is required for maintenance of EBV latency in Akata cells (related to Fig. 4).

Figure S5 jvi.01408-25-s0005.tiff Episomal AFMACS system for enrichment of the cells with lytic EBV (related to Fig. 5).

Figure S6 jvi.01408-25-s0006.tiff Quantitative proteomics analysis of lytic EBV infection and USP17 expression in the lytic stage of gamma-herpesvirus infection (related to Fig. 6).

Supplemental material jvi.01408-25-s0007.docx Legends for Fig. S1 to S6; Tables S1, S3, S4, and S7.

Table S2 jvi.01408-25-s0008.xlsx Nuclear proteins identified by the PICh experiment.

Table S5 jvi.01408-25-s0009.xlsx CRISPR gRNA sequences used in this project.

Table S6 jvi.01408-25-s0010.xlsx PICh probe sequences used in this project.

ASM does not own the copyrights to Supplemental Material that may be linked to, or accessed through, an article. The authors have granted ASM a non-exclusive, world-wide license to publish the Supplemental Material files. Please contact the corresponding author directly for reuse.
