# Locus-specific proteomics identifies novel regulators of Epstein-Barr virus lytic reactivation

**DOI:** 10.1128/jvi.01408-25

**Published:** 2026-03-17

**Authors:** Daniel E. Greaves, Ildar Gabaev, James C. Williamson, Stuart Bloor, Sergio Martinez Cuesta, Nicholas J. Matheson, Lori Frappier, Jérôme Déjardin, Paul J. Lehner

**Affiliations:** a Department of Medicine, University of Cambridge, Hills Road, Cambridge, UK; b Cambridge Institute for Therapeutic Immunology and Infectious Disease (CITIID), University of Cambridge, Puddicombe Way, Cambridge, UK; c Data Sciences and Quantitative Biology, Discovery Sciences, R&D BioPharmaceuticals, AstraZeneca, Cambridge, UK; d NHS Blood and Transplant, Cambridge, UK; e Department of Molecular Genetics, University of Toronto, Toronto, Canada; e Institute of Human Genetics, CNRS-UMR9002- Université de Montpellier, France

**Keywords:** Epstein-Barr virus, locus specific proteomics, epigenetics, virus latency, virus activation, Polycomb complex, NuRD complex, de-ubiquitinase

## Abstract

The Epstein Barr virus (EBV) is a human gammaherpesvirus which infects over 90% of the global population and is associated with lymphoid and epithelioid cancers. After infection, EBV enters a latent state in B-cells, whereby the viral genome persists as a nuclear episome maintained by expression of a small number of latency-associated viral proteins. The lytic viral proteins, required for DNA replication and virion production, are silenced by cellular epigenetic mechanisms. The immediate-early lytic gene BZLF1 is the most important target for transcriptional repression, as its expression triggers the lytic cascade. To gain insight into the factors restricting BZLF1 expression, we used the PICh method of locus-specific proteomics (***P***roteomics of ***I***solated ***Ch***romatin segments) to identify proteins which occupy BZLF1 promoter DNA. We identified more than 30 proteins associated with the BZLF1 promoter, including the nucleosome remodeler CHD4 and components of the Polycomb PRC1 complex. We show that CHD4 and PRC1 components are novel repressors of BZLF1 gene expression and that both are required to prevent spontaneous lytic reactivation in Burkitt lymphoma cells. We also reveal a marked, cell-wide loss of the PRC1 histone mark (H2AK119Ub) during the lytic cycle which is dependent on immediate-early and early lytic gene expression. A proteomic analysis of Burkitt lymphoma cells containing lytic EBV identified upregulation of USP17, a de-ubiquitinase capable of H2AK119Ub removal. Taken together, our study demonstrates the power of proteomic approaches to identify repressors of EBV reactivation, and provides new insight into how EBV manipulates epigenetic mechanisms during the lytic cycle.

## Introduction

The Epstein-Barr virus (EBV) is a human herpesvirus which infects over 90% of the human population and is strongly associated with lymphoid and epithelioid cancers (1) including Hodgkin lymphoma, post-transplant lymphoproliferative disorder, nasopharyngeal carcinomas and a subset of gastric cancers. EBV-associated malignancies are thought to account for approximately 1-1.5% of cancers worldwide (2). EBV, like other herpesviruses, enters a latent state after infection which persists for the lifetime of the host. Although low-level reactivation of the virus periodically occurs, this does not generally result in clinical symptoms. The oncogenic potential of EBV comes from viral proteins expressed during latent infection which provoke uncontrolled proliferation of the host cells and can result in malignant transformation.

EBV initially infects oropharyngeal epithelial cells and is then transmitted to B-cells circulating through the oral cavity. In infected B-cells, the EBV genome enters the nucleus where it is circularised and chromatinised (3). A coordinated sequence of viral protein expression then occurs as the infected cells progress through different stages of viral latency. In early latency (stages IIb and III), expression of multiple viral genes, including EBNA2, EBNA 3A, B & C, EBNALP and LMP1/2, transforms the B-cells into proliferating lymphoblasts. These cells are highly immunogenic and targeted by cytotoxic T-lymphocytes (CTL), favouring selection of a more restrictive latency pattern. The combination of immune selection and passage of infected cells through lymph node germinal centres eventually leads to the establishment of latent EBV in a small pool of memory B-cells. These cells have a latency type I pattern, where expression from the viral genome is minimal and consists only of non-coding RNAs and the EBNA1 protein, which tethers the EBV genome to host chromatin.

A key feature of the EBV life cycle is the ability to switch between latent and lytic infection. Expression of lytic genes occurs in a temporal sequence and begins with expression of the immediate-early gene BZLF1. Maintaining the virus in a latent state is therefore critically dependent on repression of this gene, so the chromatin state of the BZLF1 promoter (pBZLF1) is of central importance for maintaining latent infection. Multiple silencing mechanisms coexist, with some redundancy as disruption of a single component results in only a fraction of cells entering the lytic cycle. The histone marks occupying pBZLF1 are characteristic of repressive chromatin, and include the H3K27me3 mark deposited by the PRC2 Polycomb complex (4–6), as well as H3K9me3, maintained by the TRIM28-SETDB1 axis (5, 7–9). H3K9me3 appears to be less important for repression than H3K27me3 as it is not removed upon lytic reactivation (5, 10). Histone deacetylation of lytic-stage promoters, including pBZLF1, is seen during latent infection and is reversed when the virus enters the lytic cycle (5, 11). pBZLF1 is also targeted by sequence-specific DNA binding proteins including ZEB1/2, MEF2D and JDP2, which recruit histone deacetylase (HDAC) enzymes (12–15). The linker histone H1, which is associated with nucleosome compaction, occupies pBZLF1 and also acts as a repressor (16). The higher order chromatin structure of the latent EBV genome also maintains silencing of pBZLF1: the transcription factor MYC prevents looping between pBZLF1 and the origin of lytic replication, OriLyt, to restrict OriLyt-mediated enhancer activity (17).

How the latent-lytic switch occurs in vivo is not fully understood, but the most likely physiological triggers are B-cell receptor (BCR) signalling and plasma cell differentiation (18). BCR activation triggers a signalling pathway which culminates in binding of a series of transcription factors to pBZLF1, including CREB, C/EBP-beta, ATF-1, XBP1 and EGR1 (19–23). Plasma cell differentiation increases levels of host factors BLIMP1 and XBP1s, both of which also induce BZLF1 expression (17, 24, 25), triggering a cascade of approximately 30 early lytic genes, whose function includes formation of replication compartments within the nucleus and viral DNA replication. Following DNA replication, around 30 late lytic genes are then expressed which coordinate virion formation and secretion.

The sequence of pBZLF1 has been defined and extensively investigated using candidate gene approaches, but an unbiased assessment of the chromatin proteins associated with pBZLF1 has not been undertaken and could identify new repressors. Mass spectrometry-based methods of ‘locus specific proteomics’ allow purification of proteins bound to specific DNA sequences of interest and includes PICh (*P*roteomics of *I*solated *Ch*romatin segments), in which custom-made nucleic acid probes are used to perform a pulldown of both the target DNA sequence and associated proteins (26). PICh has been successfully used to profile multiple genomic loci and identified both known and novel binding proteins (26–29). To gain an unbiased view of chromatin proteins associated with pBZLF1 we performed PICh on pBZLF1 and identified binding of the nucleosome re-modeler CHD4 and the Polycomb complex PRC1. Knockdown experiments confirmed that both repressors are required to maintain EBV latency in Akata B-cells. Furthermore, we found that a complete cellular loss of the PRC1 histone mark, H2AK119Ub, occurs during viral lytic reactivation and this loss is dependent on expression of EBV immediate-early and early lytic genes. Proteomic analysis of cells undergoing lytic EBV infection identified upregulation of the human de-ubiquitinase USP17 as the likely mechanism by which H2AK119Ub is removed.

## Results

### Generation of a pBZLF1-GFP reporter system compatible with Proteomics of Isolated Chromatin segments (PICh)

The minimal promoter of BZLF1 is well defined and has binding sites for both transcriptional repressors and activators mapped using in-vitro methods e.g. EMSA (12, 18, 30, 31). Advances in proteomics now allow chromatin composition at individual genomic loci to be characterised *in vivo*, with the ability to systematically identify key regulators at specific loci, without making *a priori* assumptions. However, despite an enrichment factor of ~10,000 fold (32), PICh still requires a highly abundant target locus and for a single 1kb locus requires ~200 copies per cell. EBV is present at ~10-60 copies per cell in most B-cell lines and it was not therefore possible to perform PICh over pBZLF1 in cells containing wild-type latent EBV, requiring an alternative approach.

**Figure 1:**
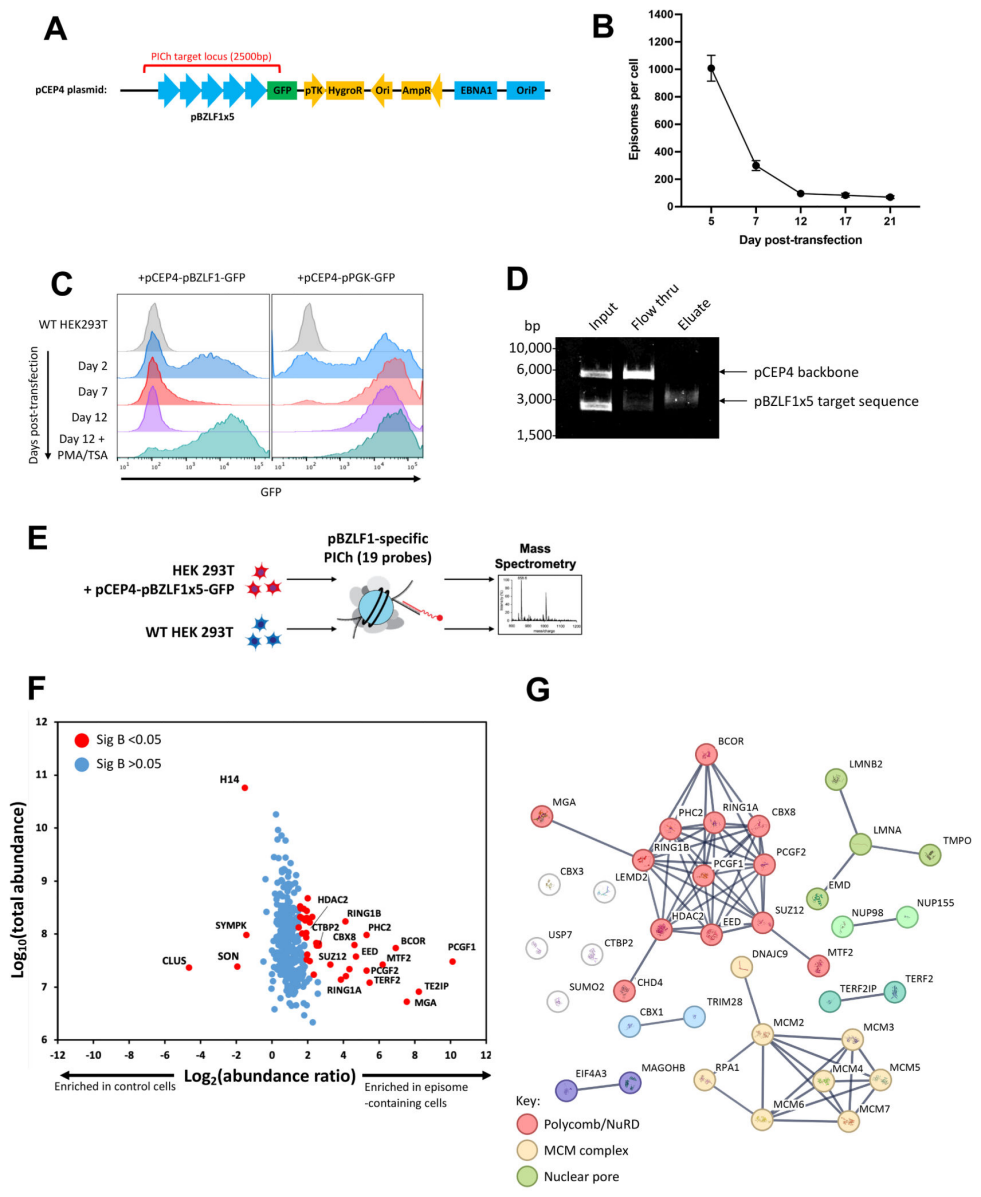
Proteomics of Isolated Chromatin segments (PICh) identifies binding of CHD4 and PRC1 to EBV BZLF1 promoter. **(A)** Schematic diagram of the pCEP4-pBZLF1(x5)-GFP reporter system compatible with PICh analysis. Five copies of pBZLF1 were cloned upstream of the GFP coding sequence into the pCEP4 episomal vector which contains EBNA1 and OriP elements as well as antibiotic resistance cassettes. The PICh target locus is indicated by a red bracket. (**B**) RT-qPCR analysis of the episome copy number. HEK293T were transfected with pCEP4-pBZLF1(x5)-GFP plasmid, harvested at indicated timepoints and subjected to DNA extraction and RT-qPCR analysis with HygroR. specific primers. Episome copy number per cell was calculated using absolute quantitation, with dilutions of prepared plasmid DNA as standards. Cellular DNA was normalised to GAPDH. Error bars denote variance between three technical replicates. (**C**) pBZLF1(x5)-GFP reporter is progressively silenced following transfection into HEK293T cells. Cells were transfected with pCEP4-pBZLF1(x5)-GFP reporter or pCEP4-pPGK-GFP control plasmid, selected with hygromycin starting at day 2, harvested at indicated timepoints and analysed by flow cytometry. At day 12 following transfection, cells were stimulated with a combination of PMA, A23187 and trichostatin A (TSA) and analysed by flow cytometry 24 hours later. (**D**) PICh probes retrieve the target locus DNA in plasmid capture assay. The pCEP4-pBZLF1(x5)-GFP plasmid was treated with *BssHII* and *NotI* endonucleases to separate the target locus from the backbone (input). The digested DNA was then incubated with an equimolar mix of the 19 PICh probes and probe-DNA hybrids were retrieved using streptavidin beads (eluate). The supernatant in the probe formed upon magnetic bead separation was collected as flow-through. 1/10 of each sample was resolved on agarose gel. (**E**) Schematic diagram of pBZLF1 PICh experiment. HEK293T cells harbouring the repressed pCEP4-pBZLF1(x5)-GFP episomes and control untransfected HEK293T cells were subjected to chromatin isolation followed by tiling with an equimolar mix of 19 PICh probes. Protein eluates from both samples were analysed by mass spectrometry. **(F)** Distribution of protein abundance ratios (episome-containing/control cells) vs total protein abundance for 357 nuclear proteins identified in the pBZLF1 PICh experiment. Outlier protein abundance ratios (Significance B <0.05) are highlighted (red dots). (**G**) STRINGDB analysis of the 43 nuclear proteins significantly enriched in the cells harbouring the pCEP4-pBZLF1(x5)-GFP episome. K-means clustering was used to separate proteins into categories, with three clear groups identified (see key). Network edges between nodes represent high confidence interactions (>0.9).

We therefore developed a plasmid-based model system of pBZLF1 silencing in HEK 293T cells. These cells were chosen because they are highly permissive to transfection and capable of maintaining plasmids at high copy number. To maximise the amount of target material for PICh, pBZLF1 was multimerized five-fold and cloned into a pCEP4 vector encoding EBV EBNA1 and OriP elements which ensure maintenance as a nuclear episome, similar to EBV (Fig 1A). The pBZLF1x5 –GFP reporter was lipofected into HEK293T cells and 12 days post-transfection the copy number was ~100 copies per cell, as measured by RT-qPCR (Fig 1B). A GFP coding sequence inserted downstream of pBZLF1x5 enabled measurement of promoter activity by flow cytometry. As expected, the GFP signal was progressively silenced following transfection into HEK 293T cells (Fig 1C) but was restored following treatment with a combination of the protein kinase C activator PMA and histone deacetylase inhibitor TSA (Fig 1C) or by overexpression of BZLF1 protein (Fig S1A). By contrast, the same vector containing GFP under the control of the phosphoglycerate kinase (pPGK) promoter showed no loss of GFP signal after transfection, implying that the silencing process was specific to pBZLF1 (Fig 1C). To ensure biological relevance, the plasmid was also electroporated into EBV-negative Akata cells where a similar process of reversible pBZLF1-GFP silencing was observed (Fig S1B). The episome copy number per cell for EBV-negative Akata was approximately 40 episomes per cell, which was lower than HEK 293T and considered to be insufficient for PICh (Fig S1C)

The combination of high episome copy number in HEK293T cells and multimerized pBZLF1 provided sufficient PICh target material so we designed a series of 19 desthiobiotinylated 2’ F-RNA probes to tile over the pBZLF1x5 sequence with a 300bp overlap into the vector on each side. To confirm specific capture of the target sequence, the target was excised from the backbone vector using restriction enzymes, and both target and empty backbone incubated with the desthiobiotinylated probes *in vitro*. Streptavidin bead pulldown of the probe-DNA hybrids selectively retrieved only the pBZLF1x5 target sequence (Fig 1D).

### Proteomics of Isolated Chromatin segments (PICh) identifies binding of CHD4 and Polycomb PRC1 to pBZLF1

Having confirmed the probe specificity, the PICh experiment was performed in HEK 293T cells harbouring the silenced pBZLF1-GFP episome, using wild-type HEK 293T cells as the control (Fig 1E). Chromatin was then extracted from 4 × 10^9^ cells and probed with an equimolar mix of all 19 probes. Following streptavidin bead pulldown and extensive washing, samples were subjected to mass spectrometry. A total of 1363 proteins were identified by ≥2 peptides, of which 357 localized to the nucleus based on GOCC annotation. To determine which of these proteins were specifically bound to pBZLF1, we compared their relative abundances from cells with and without the silenced pBZLF1-GFP episome. This revealed 39 nuclear proteins to be significantly enriched in the episome-containing cells (Significance B <0.05)) (Fig 1F, supplementary tables 1 & 2) (33). Cluster analysis of the enriched proteins using STRING-DB identified three main groups: (i) transcriptional repressors associated with the Polycomb and Nucleosome Remodeling and Deacetylation (NuRD) complexes (ii) proteins associated with the Mini Chromosome Maintenance (MCM) complex and (iii) structural proteins related to the nuclear pore (Fig 1G). The identification of components of the Polycomb complex increased our confidence that the PICh experiment had successfully retrieved the pBZLF1 DNA, as PRC2 is a major repressor of latent EBV (4–6). Further evidence suggesting that the PICh experiment was pBZLF1-specific included: (i) enriched proteins contained the two reported pBZLF1 repressors HDAC2 (34) and TRIM28 (9, 35) (ii) the MCM complex interacts with the EBV OriP sequence present on the episome upstream of pBZLF1 (36, 37) (iii) TRF2, a telomere interacting protein is also reported to bind OriP (38, 39). Of particular interest among the transcriptional repressors was the nucleosome remodeler CHD4 and the multiple highly enriched components of the Polycomb PRC1 complex: RING1A, RING1B, PCGF1, PCGF2, PHC2, BCOR and MGA. Both CHD4 and PRC1 have well-described roles in silencing of mammalian genes, through nucleosome compaction and histone 2A ubiquitination respectively, but neither are known repressors of pBZLF1, so these were investigated further.

### CHD4 and PRC1 repress the pBZLF1-GFP reporter in EBV-negative Akata Burkitt lymphoma cells

**Figure 2:**
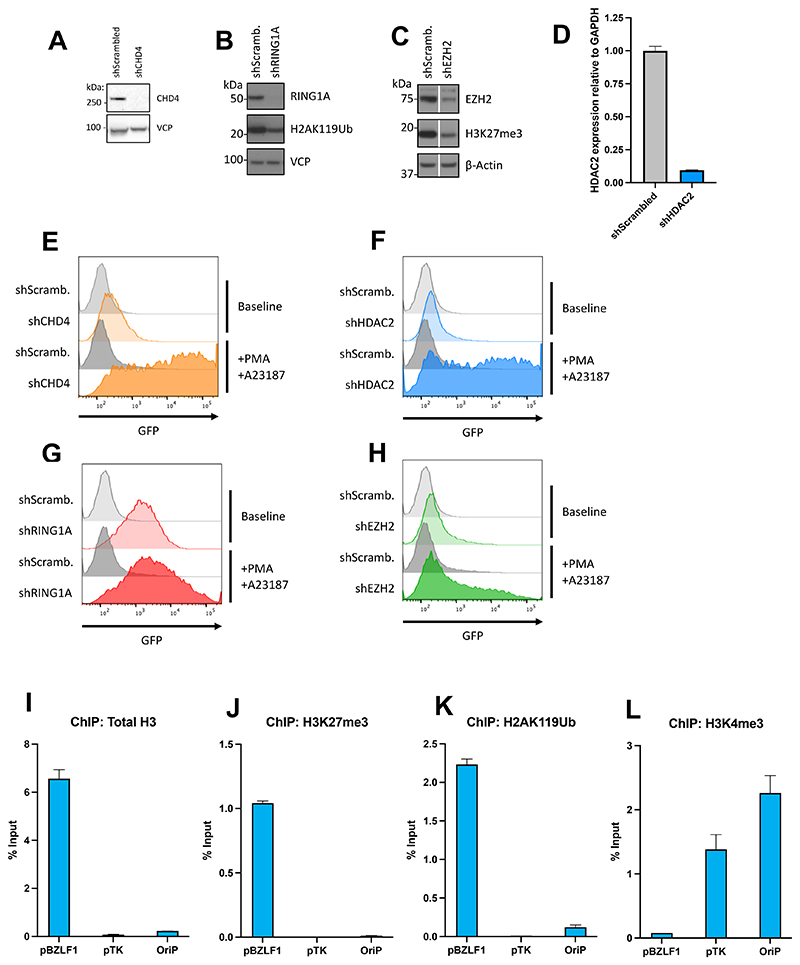
CHD4 and PRC1 repress the pBZLF1(x5)-GFP reporter in EBV-negative Akata Burkitt lymphoma cells. **(A-C)** shRNA-mediated knockdowns of CHD4 **(A)**, RING1A **(B)** and EZH2 **(C)**. Akata cells were transduced with lentiviruses expressing the indicated shRNAs and control scrambled shRNA, lysed at day 5 and analysed by immunoblot with the specific antibodies. **(D)** shRNA-mediated knockdown HDAC2. Akata cells were processed as described in (A-C) and subjected to RT-qPCR analysis with primers specific for *HDAC2* and *GAPDH*. Data are presented as mean of *n* = 3 technical replicates ± s.d. (**E-H**) Flow cytometry analysis of GFP expression in EBV-negative Akata cells harbouring the pBZLF1(x5)-GFP reporter and expressing the indicated shRNAs from the panel (A-D). PMA and A23187 were added 24 hours prior to flow cytometry analysis. (**I – L**) ChIP-qPCR analysis of EBV-negative Akata cells harbouring pCEP4-pBZLF1(x5)-GFP reporter construct using the indicated antibodies.

We selected the following hits for validation: CHD4, HDAC2 (components of the NuRD complex) and RING1A (the dominant enzymatic component of PRC1 in Akata and Raji Burkitt lymphoma cell lines, Fig S2). As a comparator, the PRC2 enzymatic component EZH2, which is a known repressor of pBZLF1 (4, 5), was also validated. shRNAs were designed against the target proteins and the efficacy of knockdowns confirmed by immunoblot analysis for CHD4, RING1A and EZH2 (Fig 2A-C) and RT-qPCR for HDAC2 (Fig 2D). Notably, knockdowns of RING1A and EZH2 also resulted in reduction of the PRC1 histone mark (H2AK119Ub) and PRC2 histone mark (H3K27me3), respectively (Fig 2B-C). To ensure biological relevance for EBV, shRNA knockdown experiments were then performed in EBV-negative Akata Burkitt lymphoma cells electroporated with the pBZLF1-GFP reporter plasmid. We then set out to analyse GFP expression in the cells with knockdowns and additionally treated the portions of transduced cells with PMA. Flow cytometry analysis showed that CHD4 depletion increased GFP expression compared with the scrambled shRNA control and a further increase was seen upon stimulating of the cells with PMA (Fig 2E). Depletion of HDAC2 had minimal effect on GFP expression at baseline but also showed a marked increase in GFP expression following PMA stimulation (Fig 2F) These results suggested that CHD4 and HDAC2 had overlapping roles in antagonizing PMA-mediated stimulation of pBZLF1 expression, while CHD4, but not HDAC2, was required for silencing spontaneous pBZLF1 expression. Depletion of RING1A was sufficient to significantly increase pBZLF1-GFP expression (Fig 2G), whereas by comparison depletion of the PRC2 enzyme EZH2 led to only a very small increase in GFP expression (Fig 2H). The addition of PMA substantially increased GFP expression in both the RING1A-depleted and EZH2-depleted cells (Fig 2G-H). Like CHD4, RING1A may therefore play a role in repressing spontaneous pBZLF1-GFP expression, whereas EZH2 is primarily involved in silencing PMA-induced pBZLF1-GFP expression.

To determine pBZLF1 occupancy of the Polycomb repressors we performed ChIP-PCR. This confirmed occupation of pBZLF1 by both H2AK119Ub (PRC1) and H3K27me3 (PRC2), compared with transcriptionally active regions of the episome where the Polycomb marks were not identified (Fig 2I-L). Likewise, PRC1 and PRC2 did not occupy the active pGK promoter driving GFP (Figs S3A-D). In summary, these experiments suggest the pBZLF1 promoter is silenced by the NuRD complex proteins CHD4 and HDAC2 as well as PRC1 core protein RING1A and provide further support that the PICh experiment was pBZLF1-specific.

### CHD4 is a repressor of spontaneous lytic reactivation in Akata cells

**Figure 3:**
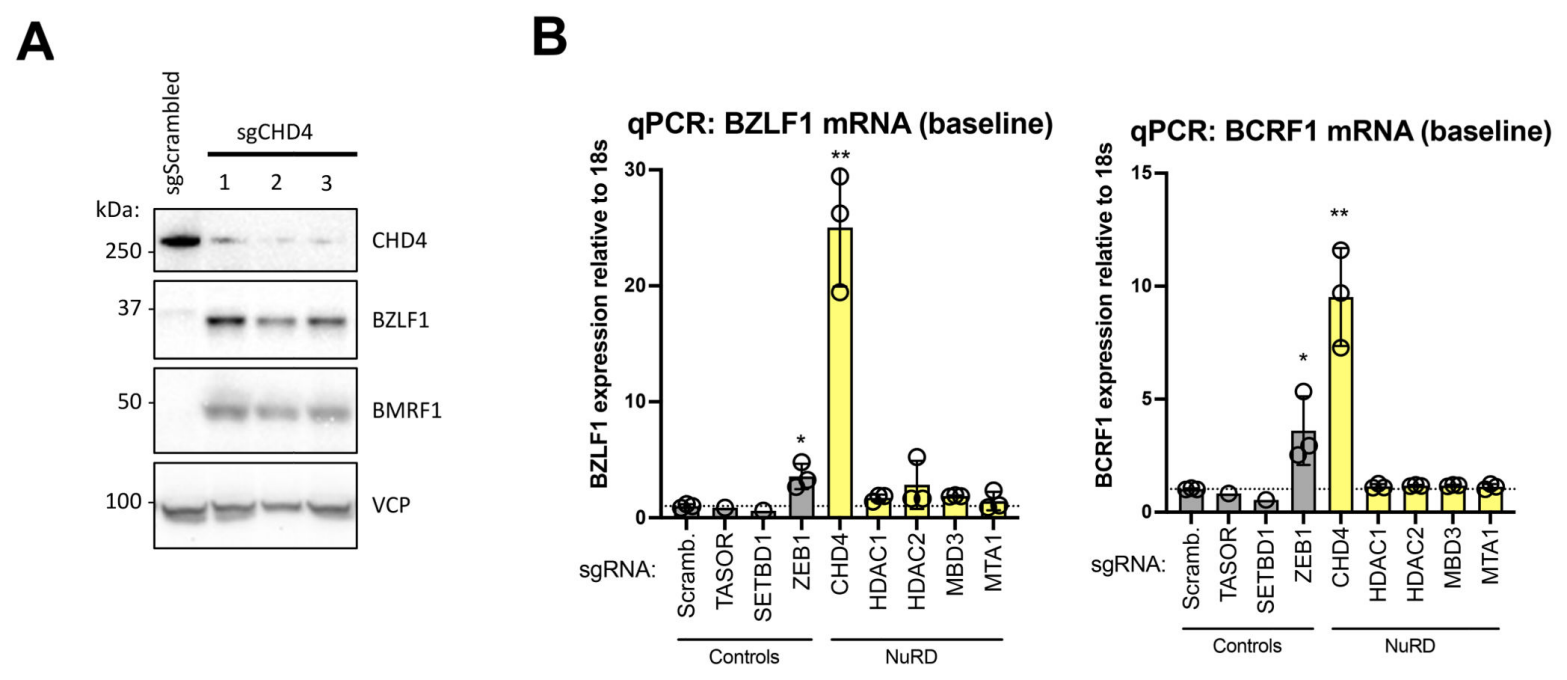
CHD4 is a repressor of spontaneous EBV lytic reactivation in Akata cells. **(A)** CRISPR-Cas9-mediated depletion of CHD4 results in expression of lytic EBV gene products. EBV-positive Akata Cas9 cells were transduced with lentiviruses expressing CHD4-specific sgRNAs, lysed and subjected to immunoblot analysis with antibodies specific for CHD4, BZLF1, BMRF1 and VCP. **(B)** Lytic EBV gene expression is specifically triggered by depletion of CHD4 but not of other components of NuRD complex. RT-qPCR analysis of BZLF1 (left) and BCRF1 (right) mRNA expression in EBV-positive Akata Cas9 cells harboring sgRNAs specific for the indicated genes. The cells were transduced with lentiviruses expressing indicated sgRNAs and subjected to RNA extraction 7 days upon transduction. Three independent sgRNAs were used per gene, except for TASOR and SETDB1 (negative controls) where a single sgRNA was used. ZEB1 was used as a positive control, being a well-described repressor of EBV lytic reactivation. For each targeted protein, a paired two-tailed Student’s *t*-test was used to assess statistical significance versus the scrambled control (*p value <0.05, **p value <0.01). Data are presented as mean of *n* = 3 biological replicates ± s.d.

Having confirmed that CHD4 is required for silencing the pBZLF1-GFP reporter in EBV-negative Akata cells, we wanted to determine its role in repressing EBV lytic reactivation. We therefore used CRISPR-Cas9 to delete CHD4 from EBV-positive Akata cells and examined expression of EBV lytic genes. Three independent CRISPR sgRNAs targeting CHD4 all provoked expression of both the immediate early lytic gene BZLF1 and early gene BMRF1 in Cas9+ Akata cells (Fig 3A). CHD4 depletion also increased late lytic gene BCRF1 expression, suggesting that full lytic reactivation was triggered (Fig 3B – **right panel**). To investigate whether CHD4 acted alone to repress EBV, or as part of the NuRD complex, four additional NuRD components were also targeted by CRISPR: HDAC1, HDAC2, MBD3 and MTA1. All four proteins were identified in the PICh experiment, although only HDAC2 was significantly enriched. Three sgRNAs targeting ZEB1 were used as a positive control, as this protein is a well described repressor of BZLF1 (30, 40). As negative controls, three scrambled sgRNAs were used, along with a single sgRNA each for TASOR and SETDB1, chromatin modifiers not involved in EBV repression. Compared with the negative controls, only CHD4 deletion increased spontaneous BZLF1 mRNA expression (Fig 3B – **left panel**), suggesting that under the conditions used CHD4 was the only NuRD complex component involved in EBV repression. How CHD4 is recruited to the EBV genome and whether it suppresses spontaneous lytic reactivation alone or as part of a multi-protein complex requires further examination.

### Polycomb PRC1 represses BZLF1 expression and occupies the EBV lytic gene promoters in a PRC2-independent manner

Polycomb PRC1 is structurally diverse, consisting of a core assembly of RING1A or B plus one of the six PCGF proteins. The PCGF proteins determine subsequent interactions with other PRC1-associated proteins, which in turn determine how the complex is recruited to the genome. Classically, the so-called ‘canonical’ PRC1 complexes containing PCGF2/4 are recruited to areas of the genome occupied by PRC2, whereas the ‘variant’ PRC1 complexes containing PCGF1/3/5/6 are PRC2 independent and recruited by transcription factors or to genomic features such as CpG islands (Fig 4A). As our PICh experiment identified multiple PRC1 components from both canonical and variant subcomplexes, we used CRISPR-Cas9 in EBV-positive Akata cells to determine how depletion of different PRC1-associated proteins affected spontaneous BZLF1 expression. Three independent sgRNAs were designed for each of the following: the PRC1 E3 ubiquitin ligase RING1A, canonical PRC1 components PCGF2, PCGF4, PHC1, PHC2, CBX2, CBX8 and variant PRC1 components RYBP, BCOR, KDM2B, MGA, L3MBTL2, CBX3. As a comparator, sgRNAs were also designed to target PRC2 components EZH2, SUZ12, EED and MTF2. SgRNAs targeting ZEB1 were used as a positive control, whereas scrambled sgRNAs, TASOR and SETDB1 were used as negative controls. The sgRNAs targeting RING1A resulted in ~4-fold increase in BZLF1 expression, while a smaller, but still significant, increase was seen in cells depleted of canonical PRC1 components PCGF2 and PHC2 and the variant PRC1 components CBX8, RYBP, BCOR and KDM2B (Fig 4B **left panel**). An increase in spontaneous expression of the late EBV-viral gene BCRF1 was also observed, indicating that the sgRNA-expressing cells had entered the full lytic cycle (Fig 4B **right panel**). Following treatment of Akata cells with anti-IgG to stimulate lytic reactivation, intracellular EBV genomic DNA significantly increased in cells depleted of RING1A, the canonical PRC1 components PCGF2 and PHC1, and the variant components RYBP, PCGF, BCOR and MGA (Fig S4A). RING1A-depleted Akata cells were also analysed by flow cytometry with co-staining for BZLF1 and H2AK119Ub (Fig 4C). This also showed an increase in lytic reactivation following anti-IgG treatment in cells lacking H2AK119Ub, indicating a synergistic effect between RING1A depletion and anti-IgG. The RING1A knockdown used for flow cytometry was confirmed by immunoblot (Fig 4E). Taken together, these results suggest that PRC1 is required for EBV latency in Akata cells and that both canonical and variant PRC1sub-complexes are likely to be involved. By comparison, depletion of PRC2-associated proteins (EZH2, SUZ12, EED and MTF2) had a small effect on spontaneous BZLF1 expression (Fig 4B **left**) but did show a synergistic effect when combined with anti-IgG (Fig S4A, 4D &F). Occupancy of EBV lytic gene promoters by H2AK119Ub and H3K27me3 was confirmed by ChIP-PCR (Fig S4 B-E). To investigate how PRC1 was recruited to the EBV genome, we performed ChIP-PCR with H2AK119Ub- and H3K27me3-specific antibodies in EBV-positive Akata Cas9 cells following depletion of the core PRC1 subunit RING1A, members of both canonical and variant PRC1 pathways (PCGF2, PCGF6, BCOR) as well as depletion of the PRC2 methyltransferase EZH2. Disruption of RING1A or EZH2 markedly reduced H2AK119Ub or H3K27me3 occupancy, respectively (Figs 4G and 4H). In contrast, depletion of canonical or variant PRC1 components or PRC2 (EZH2), only partially reduced H2AK119Ub occupancy of pBZLF1 promoter, consistent with a multifactorial recruitment mechanism at least partially independent of PRC2 (Fig 4G). At the same time, no reduction in H3K27me3 occupancy of pBZLF1 promoter was observed upon depletion of RING1A, PCGF2, PCGF6 or BCOR in these cells (Fig 4H), suggesting that PRC2 recruitment to pBZLF1 was, in turn, independent of PRC1. Altogether, our data show that BZLF1 expression in EBV episomes is repressed not only by PRC2, but also by PRC1 and that this repression is mutually independent. Furthermore, PRC1 occupancy of pBZLF1 is independent of PRC2.

### The PRC1 histone mark, H2AK119Ub, is depleted from both EBV and human loci during EBV lytic reactivation

**Figure 5:**
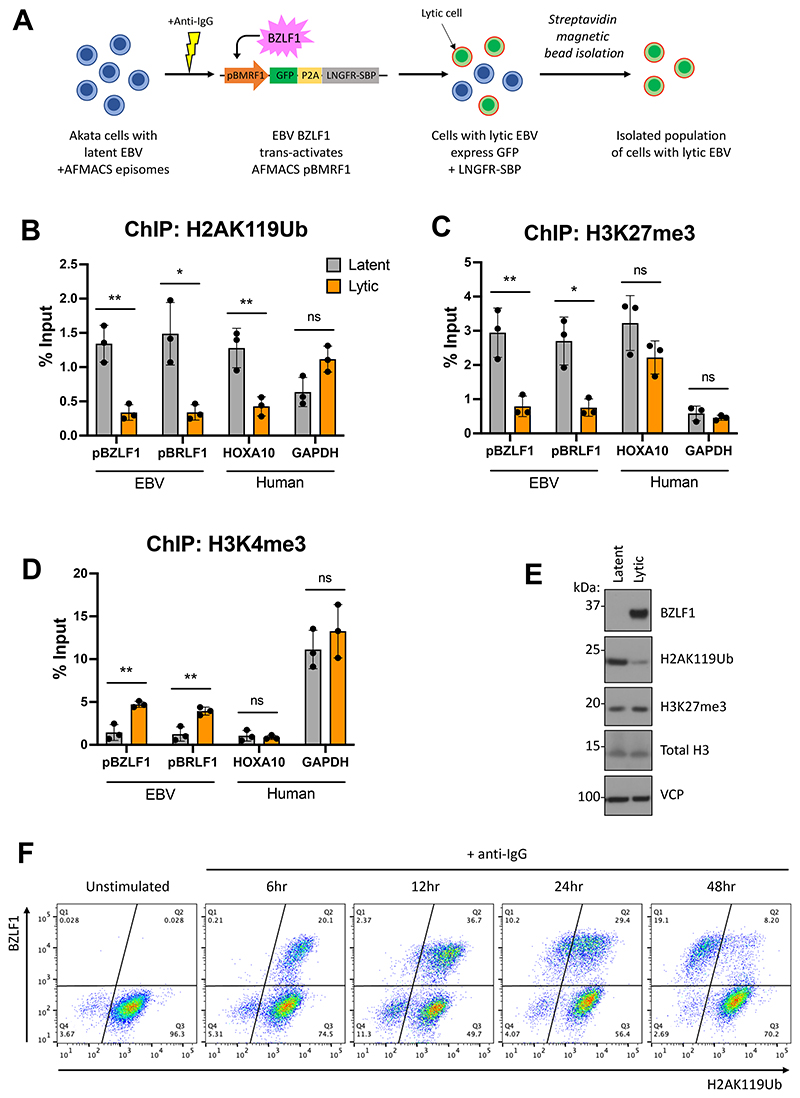
H2AK119Ub is depleted from both EBV and human loci during lytic reactivation. **(A)** Workflow schematic for isolation of Akata cells with lytic EBV using AFMACS enrichment system. Akata cells harbouring latent EBV and pCEP4 episomal vector with pBMRF1-GFP-P2A-LNGFR-SBP cassette were stimulated with anti-IgG antibody for 12 hours. The cells with lytic EBV (positive for both LNGFR-SBP and GFP) were then isolated using streptavidin magnetic beads. **(B, C, D)** ChIP qPCR analysis with antibodies specific for H2AK119Ub, H3K27me3 and H3K4me3 histone marks in EBV-positive Akata cells. Lytic cycle of EBV was induced as described in Figure 5A. 200uM acyclovir was added to cells prior to stimulation to prevent viral genome replication. The comparison is made between AFMACS-enriched Akata cell population harbouring lytic EBV (orange bars) and unstimulated cells harbouring latent EBV (grey bars) for both viral (BZLF1 and BRLF1 promoter regions) and human (HOXA10, GAPDH) loci. **(E)** Immunoblot analysis of Akata cells with latent and lytic EBV. The cells were treated and enriched by AFMACS as outlined in Figure 5A or left untreated, lysed and subjected to immunoblot analysis with antibodies specific for BZLF1, H2AK119Ub, H3K4me3, H3 and VCP. **(F)** Flow cytometry analysis of EBV-positive Akata cells upon anti-IgG stimulation. The cells were treated with anti-IgG antibody or left untreated, fixed at indicated timepoints, permeabilised and stained with antibodies specific for BZLF1 and H2AK119Ub.

The loss of repressive histone marks, such as H3K27me3, from the EBV genome during lytic reactivation is well described (5) making it important to determine whether this was also true for H2AK119Ub. This analysis was challenging as it requires isolation of a pure population of lytic EBV-positive Akata cells for ChIP-PCR. We therefore took advantage of Antibody-Free Magnetic Cell Sorting (AFMACS), in which a low-affinity Nerve Growth Factor Receptor (LNGFR)-streptavidin binding protein (SBP) fusion protein expressed at the cell surface enables selection with streptavidin-coated magnetic beads (41, 42). Using the pCEP4 episomal vector as a backbone, we generated an AFMACS system for enrichment of cells containing lytic EBV in which the EBV BMRF1 promoter drives expression of GFP and LNGFR-SBP as a single polypeptide, separated by a P2A sequence. The BMRF1 promoter remains silent until transactivated by BZLF1 during the early EBV lytic cycle (Fig 5A and S5A). At baseline, Akata cells harbouring the pCEP4 episomal vector with the pBMRF1-GFP-P2A-LNGFR-SBP cassette expressed neither GFP nor LNGFR-SBP, but following anti-IgG stimulation and magnetic bead selection we obtained an 87% enriched population of GFP/LNGFR+ve cells (Fig S5B/C). ChIP PCR for H2AK119Ub, H3K27me3, H3K4me3 (a mark of active transcription) and total histones H2A and H3 was performed in the presence of aciclovir to block lytic viral DNA replication. We found reduced H2AK119Ub occupancy of pBZLF1 and pBRLF1 in the EBV lytic cell population with similar changes seen with H3K27me3 (Fig 5B/C). Total H2A and H3 occupancy of lytic genes was also reduced (Fig S5 E/F), as reported for Raji cells (5). Consistent with this de-repression we observed increased H3K4me3 occupancy of pBZLF1 and pBRLF1 (Fig 5D), indicating active transcription of lytic EBV genes.

To our surprise, H2AK119Ub was not only lost from EBV viral genes but was also depleted from the endogenous human HOXA10 locus in EBV lytic cells (Fig 5B). This H2AK119Ub loss from a host gene was unanticipated and suggested that loss of H2AK119Ub upon viral reactivation was not necessarily restricted to viral genes but might be more widespread. Indeed, we confirmed the loss of total cellular H2AK119Ub by immunoblot (Fig 5E), which showed a global reduction of H2AK119Ub in the lytic cell population, compared with minimal changes to H3K27me3 and total H3 (Fig 5E). These findings were further confirmed by using flow cytometry to detect H2AK119Ub in permeabilised cells (Fig 5F). This showed that loss of H2AK119Ub in the BZLF1 +ve cells was visible at 12 hours post BCR crosslinking and by 48 hours the EBV reactivating cells (BZLF1+ve population) had completely lost all cellular H2AK119 ubiquitin expression (Fig 5F). Interestingly, this meant that loss of H2AK119Ub appeared to occur more rapidly in the lytic cells isolated using AFMACS where loss of H2AK119Ub was significant by 12 hours (Fig 5E). We suggest that AFMACS-selection of cells which had entered the lytic cycle earliest was the most likely explanation for this discrepancy. No change in H2AK119Ub expression in the non-activated (BZLF1-ve) cell population was observed by either immunoblot or flow cytometry (Fig 5E/F), nor was there a widespread loss of H3K27me3 in the lytic cell population (Fig S5H). Selective loss of H2AK119Ub from lytic cells could not be attributed to B-cell activation alone, as the entire cell population showed evidence of B-cell activation (phosphorylation of Akt) after addition of anti-IgG (Fig S5D). Together, these findings suggested an active cellular H2AK119-deubiquitination driven by EBV lytic cycle reactivation. Whether this process was caused by viral or cellular effects was unclear.

### The USP17 deubiquitinating enzyme is upregulated upon EBV reactivation in a virus-dependent manner

**Figure 6:**
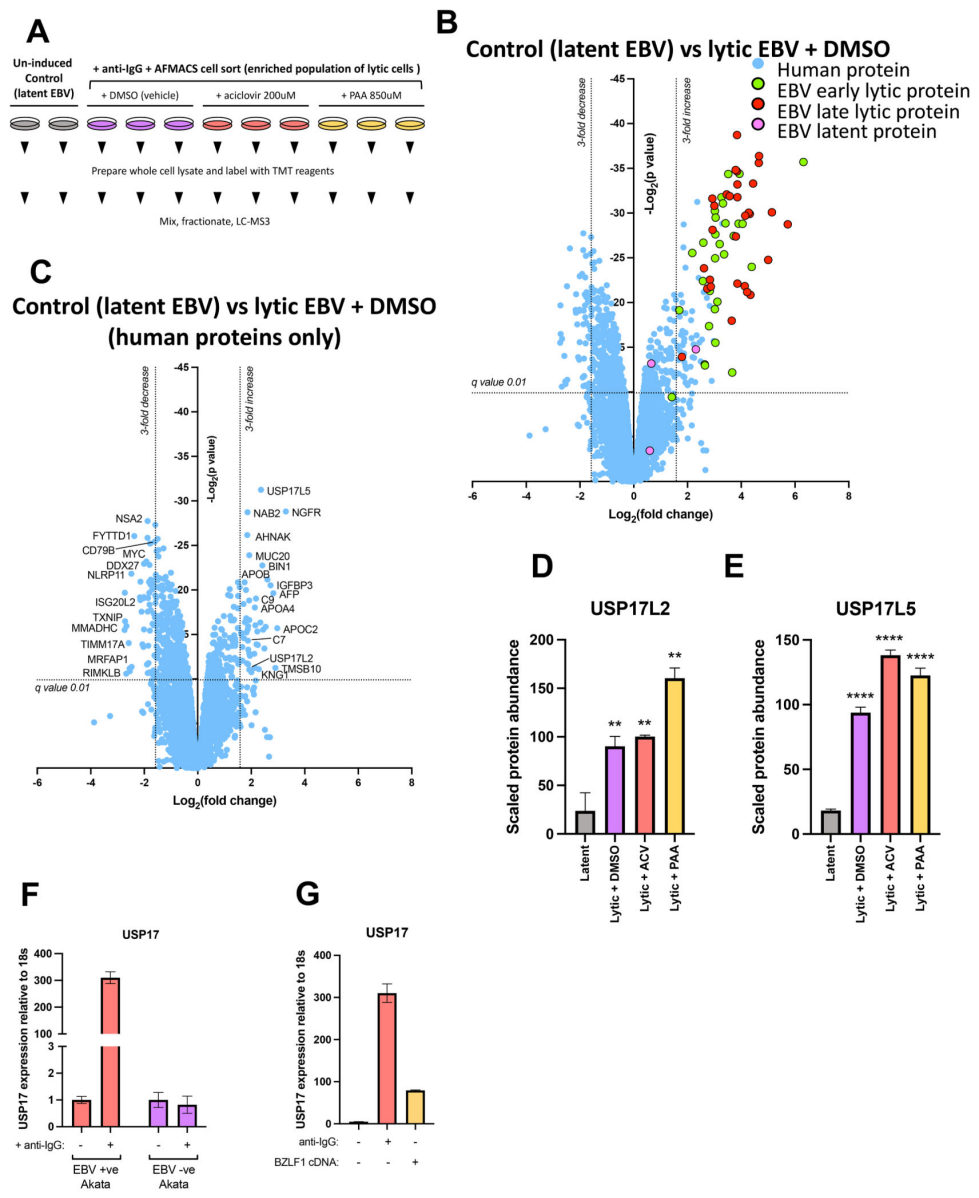
USP17 deubiquitinating enzyme is upregulated upon EBV reactivation in a virus-dependent manner. **(A)** Schematic overview of the quantitative proteomics analysis of the cells with latent versus lytic EBV infection. Akata cells harbouring latent EBV and pCEP4 episomal vector with pBMRF1-GFP-P2A-LNGFR-SBP cassette were stimulated with anti-IgG antibody for 24 hours in the presence of either DMSO (vehicle), aciclovir or PAA. Unstimulated cells were used as a control. The cells with lytic EBV (positive for both LNGFR-SBP and GFP) were then isolated using streptavidin magnetic beads, lysed and treated with trypsin. The resulting peptides were labelled with TMT reagents, mixed, fractionated and analysed by mass spectrometry. **(B**,**C)** Scatterplots display pairwise comparisons between unstimulated (control) Akata cells with latent and lytic (anti-IgG-stimulated, DMSO-treated, AFMACS-enriched cells) EBV infection. Each point represents a single protein, plotted by its log2 (fold change in abundance) versus the statistical significance (q value) of that change. The q-value was corrected for multiple hypothesis testing using the method of Benjamini-Hochberg. Dotted line: q = 0.01. Human and viral proteins are colour-coded as indicated. Scatterplot (C) shows only human proteins. **(D, E)** Scaled protein abundance of USP17L2 and USP17L5 in unstimulated (control) cells (grey) and cells with lytic EBV treated with either DMSO only (purple), aciclovir (red) or PAA (yellow) (* q value <0.05, ** <0.01, ***<0.001, ****<0.0001). **(F)** *USP17* expression in EBV-positive and EBV-negative Akata cells following stimulation with anti-IgG antibody. The cells were harvested 24 hours after stimulation, lysed and subjected to RT-qPCR analysis with primers specific for *USP17* and *18S*. **(G)** *USP17* expression in EBV-positive Akata cells following stimulation with anti-IgG antibody or transduction of cells with BZLF1-expressing lentivirus. The analysis was performed as described in the Fig 6F.

The cellular loss of H2AK119Ub during lytic EBV infection raised the question of whether this process is driven by viral or cellular factors. We reasoned that (i) the protein(s) responsible for depletion of total H2AK119Ub is likely to be affected by EBV reactivation and (ii) its change in abundance should be detectable by quantitative proteomics. We therefore applied multiplex Tandem Mass Tag (TMT)-based proteomics to resolve changes in the proteome of Akata cells with lytic EBV infection 24 hours after BCR-crosslinking (Fig 6A). The same Akata cell line harbouring the pCEP4 vector with the pBMRF1-GFP-P2A-LNGFR-SBP cassette used for the ChIP-PCR experiments (Fig 5) was again used to isolate a population of cells with lytic EBV. In addition, cells with lytic EBV reactivated in the presence of either aciclovir or PAA were included, as these inhibitors expedited H2AK119Ub removal, which could now be seen at 24 hours post stimulation (Fig S5I). Unselected and unstimulated EBV-positive Akata cells were included as a negative control (Fig 6A). We quantified a total of 7776 cellular and viral proteins. The comparison of unstimulated (latent) and stimulated (lytic) EBV-infected cells showed a significant (q <0.01) greater than 3-fold decrease in abundance of 55 cellular proteins (Fig 6B), including the B-cell receptor CD79 and transcriptional regulator MYC, both previously shown to be downregulated by lytic EBV infection (43). We also observed significant (q <0.01) upregulation of 50 cellular proteins, including previously reported complement factor C7, alpha foetoprotein (AFP) and kininogen (KNG1) (43) as well as 58 EBV lytic proteins. The addition of aciclovir or PAA resulted in selective reduction of late viral gene products (Fig S6 A-E), as would be predicted. A complete list of the human and viral proteins significantly (q <0.01) upregulated in the cells with lytic EBV infection is provided in Supplementary table 3 and downregulated proteins in Supplementary table 4. Importantly, none of the 18 Polycomb complex associated protein components detected changed abundance in the EBV lytic cell population (Fig S6F). Loss of Polycomb activity through depletion of the complex is therefore unlikely to be responsible for the profound decrease in cellular H2AK119Ub. Of particular interest among the significantly upregulated cellular proteins in the population of cells with lytic EBV were two members of the large USP17-like family of de-ubiquitinase (DUB) enzymes, USP17L2 and USP17L5 (Fig 6C). Expression of both these proteins was further increased in the lytic cells treated with either aciclovir or PAA (Fig 6 D&E) and no other DUBs showed increased expression upon EBV lytic reactivation (Fig S6G). H2AK119Ub is not known to be de-ubiquitinated by the USP17-like protein family, but as USP17-like proteins are chromatin associated and can de-ubiquitinate histone H2AX as part of the DNA damage response (44), we considered these proteins as strong candidates for further investigation.

The USP17-like family consists of at least 32 genes with >92% DNA sequence homology (45) and are distributed across two RS447 megasatellite regions on two chromosomes (4 and 8) (46). All USP17-like proteins show 98% similarity at the amino acid level, making reliable differentiation by mass spectrometry impossible. The observed upregulation of USP17L2 and USP17L5 was likely to represent a general upregulation of the entire family. Hereafter, the USP17-like family will be referred to collectively as USP17. To investigate whether USP17 upregulation during EBV lytic cycle reactivation was transcriptional, we performed RT-qPCR with primers targeting a common region of the entire USP17-like family. This showed a marked increase in USP17 RNA expression (300-fold) in lytic EBV-positive Akata cells following anti-IgG stimulation (Fig 6F). Importantly, there was no change in USP17 expression in the EBV-negative Akata control cells stimulated under the same conditions of anti-IgG stimulation (Fig 6F, S5D and S5E). USP17 mRNA expression was also increased (>70-fold) in EBV-positive Akata cells when the lytic cycle was triggered by overexpressed BZLF1 protein alone, rather than anti-IgG (Fig 6G). To determine whether lytic cycle-induced USP17 upregulation is a common feature of human gammaherpesviruses, we investigated whether USP17 is upregulated upon KSHV lytic reactivation. The iSLK.219 cell line harbours latent KSHV genomes (47) and addition of doxycycline induces transcription of the RTA transgene, which stimulates KSHV lytic reactivation. USP17 mRNA was also triggered under these conditions, suggesting that, like EBV, KSHV lytic reactivation upregulates USP17 expression (Fig S6K). Together these data show that USP17 expression is induced in lytic phase of both EBV and KSHV. In the case of EBV, this induction is dependent on virus reactivation and not simply a consequence of B-cell activation. Consistent with the proteomic data, aciclovir did not block the USP17 upregulation observed in Akata cells with lytic EBV (Fig S6H-J) implying that an early lytic viral gene product was likely responsible for this phenomenon.

**Figure 7:**
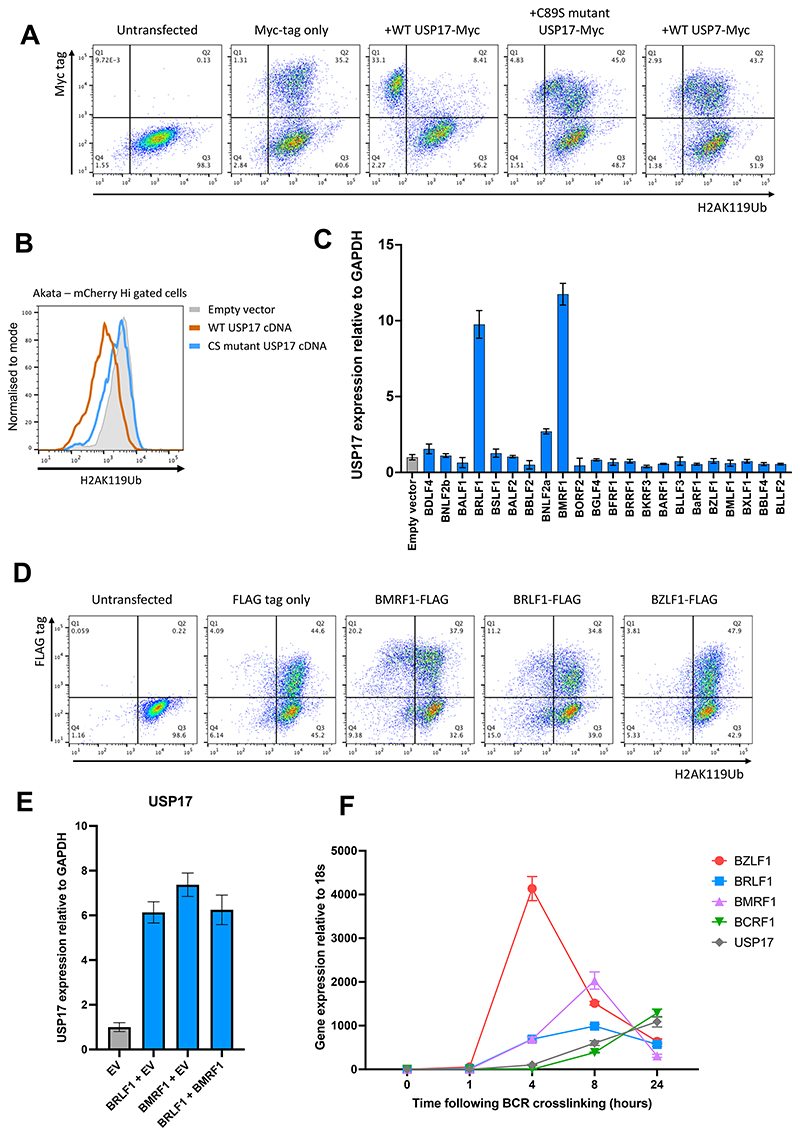
USP17 overexpression is associated with removal of H2AK119Ub. **(A)** Flow cytometry analysis of H2AK119Ub expression in cells transfected with USP17 wt and mutant constructs. HEK293T cells were transfected with vectors expressing Myc-tagged wild-type USP17, USP17 C89S mutant or control USP7 proteins, harvested 48 hours later, permeabilised and stained with the antibodies specific for H2AK119Ub and Myc-tag. Cells transfected with vector expressing Myc-tag only and untransfected cells were used as controls. **(B)** Flow cytometry analysis of EBV-positive Akata cells transduced with lentiviral vector containing either wild-type USP17-P2A-mCherry or an inactive mutant USP17-P2A-mCherry. Shown are cells stained for H2AK119Ub with mCherry-Hi gating **(C)** *USP17* expression following transfection of immediate-early and early lytic EBV ORFs. HEK293T cells were transfected with the vectors expressing indicated viral ORFs using lipofection, harvested 48 hours later, lysed and subjected to RT-qPCR analysis with primers specific for *USP17* and *GAPDH*, **(D)** flow cytometry analysis of H2AK119Ub expression in cells transfected with EBV ORFs. HEK293T cells were transfected with vectors expressing FLAG-tagged EBV BMRF1, BRLF1 and BZLF1 ORFs, harvested 48 hours later, permeabilised and stained with the antibodies specific for H2AK119Ub and FLAG-tag. Cells transfected with vector expressing FLAG-tag only and untransfected cells were used as controls. **(E)** *USP17* expression in cells co-transfected with the indicated viral ORFs. HEK293T cells were transfected using lipofection, harvested 48 hours later, lysed and subjected to RT-qPCR analysis with primers specific for *USP17* and *GAPDH*. Cells transfected with empty vector (EV) were used as a control. **(F)** Temporal kinetics of *USP17* and viral genes expression upon EBV reactivation. EBV-positive Akata cells were stimulated with anti-IgG antibody, harvested at indicated timepoints, lysed and subjected to RT-qPCR analysis with primers specific for *EBV BZLF1, BRLF1, BMRF1, BCRF1, cellular USP17* and *18S*.

To identify the potential viral protein(s) responsible, we used a set of 22 FLAG-tagged EBV ORFs from a previously published EBV gene library (48–50). Of the 22 selected ORFs, 19 had immediate-early or early lytic expression kinetics, and the expression kinetics were unclear for the remaining 3 ORFs. We overexpressed the selected viral ORFs in HEK 293T cells and analysed USP17 expression. RT-qPCR analysis showed that two EBV gene products, the transactivator protein BRLF1 and DNA-processivity factor BMRF1 induced USP17 mRNA expression (Fig 7C). A complementary flow cytometry analysis of HEK 293T cells with overexpression of both proteins confirmed a modest reduction in H2AK119Ub (Fig 7D). Co-expression of BRLF1 and BMRF1 did not have a synergistic effect on USP17 expression or de-ubiquitination of H2AK119Ub (Fig 7E). Analysis of USP17 and viral gene expression in Akata cells following EBV reactivation confirmed that mRNAs of both BMRF1 and BRLF1 were expressed prior to the upregulation of USP17 (Fig 7F). Taken together, these results suggest that USP17 is likely to be required for removal of H2AK119Ub during EBV lytic reactivation, and that transcriptional upregulation of USP17 is controlled by the viral proteins BRLF1 and BMRF1 in a non-redundant manner.

### USP17 overexpression is associated with removal of H2AK119Ub

We next set out to determine whether the increased USP17 expression was responsible for the de-ubiquitination of H2AK119Ub. Unfortunately, the USP17 antibody used in previous USP17 publications (51–53) was no longer available and, despite extensive testing of commercially available USP17-specific antibodies, we were unable to identify any suitable alternative. We therefore overexpressed Myc-tagged wild-type USP17, its catalytically inactive C89S mutant (54) and the USP7 DUB as a control in HEK 293T cells and examined intracellular H2AK119Ub expression by flow cytometry. Overexpression of wild-type USP17, but not the USP17 C89S mutant or USP7 resulted in a large reduction in H2AK119Ub expression (Fig 7A). Furthermore, reduced H2AK119Ub expression was also seen in EBV-positive Akata cells overexpressing wild-type USP17, but not the USP17 C89S mutant (Fig 7B). Taken together these experiments suggest that USP17 is able to de-ubiquitinate H2AK119Ub, and the increased USP17 expression seen following lytic EBV reactivation may therefore be responsible for the profound loss of cellular H2AK119Ub.

Finally, we wanted to formally prove that the marked increase in USP17 expression was responsible for the EBV-induced loss of H2AK119Ub, but this experiment required an effective deletion/depletion of USP17. This was technically challenging because: (i) although resting cellular levels of USP17 were effectively depleted by USP17-specific shRNAs, the very large (>100x) EBV-induced induction of USP17 overcame all attempts to effectively deplete USP17 despite the use of multiple shRNAs (ii) the complexity of the USP17 locus with multiple gene copies made it impossible to use CRISPR-Cas9 technology to either delete or deplete the USP17-expressing genes. Therefore, despite many attempts at decreasing USP17 expression with USP17-specific shRNAs and sgRNAs both individually and in combination we were unable to prevent a significant induction of USP17 following EBV reactivation (data not shown). It therefore remains unproven whether USP17 is indeed responsible for the loss of viral and cellular H2AK119Ub, or whether USP17 depletion can limit or prevent EBV lytic reactivation.

## Discussion

In this study, we developed a plasmid-based model of EBV BZLF1 promoter silencing and used it for proteomics of isolated chromatin segments (PICh) to identify cellular regulators of EBV latency. We found that the BZLF1 promoter is repressed by nucleosome remodeling factor CHD4 and components of the PRC1 complex. We show that CHD4 represses spontaneous lytic EBV reactivation and that PRC1 occupies viral lytic gene promoters in a PRC2-independent manner. Furthermore, we found that the PRC1 histone mark H2AK119Ub is depleted from both viral and cellular loci during EBV lytic reactivation. Finally, we show that lytic EBV induces expression of USP17 deubiquitinating enzyme and that overexpression of USP17 is associated with H2AK119Ub mark removal.

The role of cellular chromatin proteins for maintaining EBV latency has been intensely studied for several decades and many important repressors have been identified by mutation of promoter sequences, candidate gene approaches and, most recently, in forward genetic screens (12, 15–17, 55–58). To our knowledge, this is the first study to take a locus-specific proteomics approach to interrogate the chromatin occupying a silenced EBV promoter and indeed the first use of locus specific proteomics to target episomal DNA

The success of a PICh experiment can be determined by whether factors known to associate with the target locus are identified as well as novel proteins (26). The results of our pBZLF1 PICh experiment fulfilled these criteria as, in addition to identification of novel pBZLF1-associated proteins, PRC1 components and CHD4, we also identified several proteins that have been previously reported to bind pBZLF1. These include PRC2 components, TRIM28 and HDAC2 (4–6, 9, 34, 59). It is also important to note that several proteins known to bind pBZLF1, e.g. ZEB1/2, MEF2D, and JDP2 (12, 15, 30) were not identified in our PICh experiment. A potential explanation for this is differences in protein abundance, as upstream chromatin modifiers such as PRC2 may be more abundant at a cellular level than transcription factors. In addition, histone modifiers may occupy multiple histones associated with pBZLF1 and therefore be present at a higher density than sequence-specific binding proteins which bind pBZLF1 at a single motif. As the PICh experiment was performed in HEK 293T cells, it is also possible that known EBV repressors were not detected due to lower abundance in a non-B-cell line.

We found that CHD4 represses both pBZLF1 expression and spontaneous EBV lytic reactivation. CHD4 is a chromatin remodeling enzyme and a component of two distinct gene repression complexes, NuRD (60–62) and ChAHP (63). To our knowledge, the information on the role of CHD4 in regulation of herpesvirus gene expression has been so far limited to studies on KSHV in which CHD4 was identified as a (i) repressor of viral gene expression in *de novo* infection (64) and (ii) a key regulator of viral latency-lytic switch (65). Our results therefore extend these observations to regulation of lytic reactivation of another gamma-herpesvirus, EBV.

How CHD4 represses spontaneous EBV lytic reactivation and whether it acts alone or as part of the NuRD or ChAHP complexes is unclear. NuRD consists of the active enzymes CHD3/4/5 and HDAC1/2, which compact nucleosomes and deacetylate histones, alongside scaffold proteins GATAD2A/B, histone chaperones RBBP4/7, histone binding proteins MTA1-3 and CpG island specific binders MDB2/3 (66). NuRD recruitment to genomic loci is often guided by the binding of MTA proteins to transcription factors (67), or MBD proteins to CpG methylated DNA (68). We found that knockdown of MTA and MBD proteins in Akata cells did not lead to spontaneous BZLF1 expression suggesting that CHD4 recruitment may not be dependent on these proteins. We also found that knockdowns of HDAC1 and 2 did not provoke spontaneous EBV reactivation, which was a surprise as HDAC2 is a recognised repressor of pBZLF1 (34). Three independent sgRNAs were designed to target each gene in our validation experiments, so failure to achieve at least one effective knockdown is unlikely. A possible explanation for the lack of phenotype is redundancy between HDAC1/2 (69) meaning that both enzymes would need to be depleted for the effect to be observed. Previous studies which established a role for HDAC enzymes in EBV latency used HDAC inhibitors, such as Trichostatin A, which inhibit both HDAC1 and 2 simultaneously (4). In KSHV *de novo* infection, MBD3 and GATAD2A were identified as repressors of viral gene expression along with CHD4, and it was suggested that MBD3 may target NuRD to CpG islands in the KSHV genome (64). However, as pBZLF1 does not contain a CpG island (5) this is unlikely to account for the recruitment to EBV. CHD4-mediated repression of lytic KSHV lytic reactivation has also been shown to occur through interaction of CHD4 with latent viral protein LANA and a component of cellular ChAHP complex, ADNP (65, 70). Finally, CHD4 can also be recruited to its loci independently by other means, including direct binding to H3K9me3 (71).

The role of Polycomb PRC2 in the maintenance of EBV latency is well described (4–6), but our study suggests that PRC1 is an independent repressor of lytic reactivation. PRC1 occupies the LMP1 and 2 promoters in Burkitt lymphoma cell lines with a latency I phenotype (72), so could also target lytic gene promoters. Whether PRC1 is recruited to multiple regions of the EBV genome by the same mechanism is uncertain, as we found that occupation of pBZLF1 by H2AK119Ub was dependent on both canonical (PRC2 dependent) and non-canonical (PRC2 independent) pathways. The PRC1 co-factor KDM2B has been shown to bind pBZLF1 (73), which could explain PRC1 recruitment via the non-canonical pathway.

Loss of H2AK119Ub from pBZLF1 and pBRLF1 during lytic reactivation provided further evidence that PRC1 is a repressor of lytic reactivation. Most surprising was the complete loss of cellular H2AK119Ub following lytic reactivation, i.e. unlike PRC2, the loss was not restricted to viral genes. This global cellular loss of H2AK119Ub may represent ‘spill-over’ of the mechanism used to deubiquitinate the viral genome. The loss of H2AK119Ub in Akata cells was visible by flow cytometry from around 12 hours after onset of lytic reactivation. It would therefore seem unlikely that loss of H2AK119Ub from the viral genome is necessary for the lytic switch to occur but could be required for a later event such as viral genome replication.

To investigate changes in histone modifications during EBV reactivation, we required a pure population of cells containing lytic virus. We used the AFMACS system for this purpose as it enabled cell sorting at an early time point during the lytic cycle. An alternative method would be to isolate cells using an antibody to the EBV lytic protein GP350, which is expressed at the cell surface during reactivation (17, 43). While the latter method does not require generation of a cell line expressing the AFMACS plasmid, GP350 expression is blocked by aciclovir and so is not compatible with ChIP PCR, where aciclovir is used to prevent viral genome amplification.

Deubiquitination of multiple cellular proteins, including H2A, has been described in Akata cells containing lytic EBV and was attributed to B-cell activation, rather than to the virus itself, as similar changes were reported in EBV-negative Akata cells following BCR crosslinking (74). In contrast, our experiments clearly demonstrated that loss of H2AK119Ub only occurred in Akata cells expressing BZLF1 after BCR crosslinking even though the entire cell population showed evidence of B-cell activation. A focus for future work will be to determine whether H2AK119Ub loss occurs in other cell types containing lytic EBV if reactivation is triggered by a BCR-independent mechanism, such as BZLF1 overexpression.

The loss of cellular H2AK119Ub upon lytic reactivation led to a search for the deubiquitinase(s) (DUBs) responsible for this phenotype. We found that expression of the USP17 DUB was markedly increased on both mRNA (up to 300-fold) and protein (more than 3-fold) level during the lytic cycle and we provide three lines of evidence that this protein is likely to be responsible for H2AK119Ub de-ubiquitination: (i) our unbiased proteomic analysis showed that USP17 was the only human DUB to be significantly upregulated in lytic Akata cells, (ii) upregulation of USP17 could be induced by overexpression of two lytic viral proteins and (iii) overexpression of USP17 resulted in de-ubiquitination of H2AK119Ub. Upregulation of cellular genes during EBV lytic cycle reactivation is restricted due to virus-induced shutoff of host gene expression, mediated by canonical (non-specific destruction of mRNA by the viral exonuclease BGLF5) or non-canonical (BGLF5-independent) mechanisms (75–77). The marked increase in USP17 expression might therefore represent an attractive phenomenon for investigating how host cell genes evade these lytic EBV-induced shutoff mechanisms.

A previous proteomic analysis of expression changes in lytic Akata cells did not detect any USP17 peptides (43). As the peptides identified in our dataset are common to multiple USP17-like family members, they may have been inadvertently excluded as being unattributable to a single protein due to the unusual nature of the USP17 gene family as discussed below.

An outstanding question in this study is whether the increased expression of USP17 is indeed required for EBV lytic reactivation and replication. To address this issue definitively, effective deletion or depletion of USP17 family members is necessary. This proved to be very challenging due to the marked extent of USP17 upregulation during the lytic cycle. Multiple attempts including CRISPR-Cas9 mediated deletion with sgRNAs, CRISPR-dCas9 mediated depletions and use of multiple shRNAs covering majority of USP17 gene family members have so far been unable to reduce the upregulation of USP17 in lytic EBV sufficiently to see a phenotype. This is due to the complex and unusual genetic profile of the USP17 gene family. This gene family resides within the RS447 DNA megasatellites on chromosomes 4 and 8 (46, 78–80) and consists of tandem repeat sequences present at a high and somewhat variable copy number of between 23-103 gene family members, with substantial inter-individual variation (46). At least 32 USP17-like gene ORFs are spread over blocks of RS447 repeats and these ORFs are present on two chromosomes with at least 23 ORFs on chromosome 4 and at least 9 ORFs on chromosome 8 (81). Furthermore, each of the 32 USP17-like genes may themselves have multiple copies with significant variation between individuals (82). While our manuscript was in preparation, a cellular protein DUX4 was shown to play a critical role for lytic replication of several herpesviruses, including EBV-related gamma-herpesvirus KSHV (83). Curiously, DUX4 shares common features with USP17: (i) both DUX4 and USP17 are present in multiple copies in RS447 repeats (84, 85); (ii) like USP17, DUX4 is markedly upregulated in lytic KSHV infection (83); (iii) the expression of both genes is regulated by cellular ZEB1 repressor protein (86). Further studies are necessary to elucidate whether increased USP17 expression is required for lytic gamma-herpesvirus replication and, if this is the case, also to understand why viral latency-to-lytic switch relies on the cellular genes present in high copy numbers within DNA megasatellites.

We identified inducible expression of USP17 triggered by EBV lytic proteins BRLF1 and BMRF1. Cytokine-induced expression of USP17 in response to IL-4 and IL-6 is well described (80), which suggests that EBV may be hijacking an existing cellular pathway. Overexpression of BRLF1 and BMRF1 both induced expression of USP17 mRNA independently in HEK 293T cells, though the degree of USP17 upregulation was approximately 30-fold lower than following EBV reactivation in Akata cells possibly reflecting differences in cell type or the state of cellular activation. USP17 has a variety of known deubiquitination targets including CDC25A (54) and ELK-1 (87). While H2AK119Ub is not a recognised target, USP17 does deubiquitinate the closely related H2AX as a negative regulator of the DNA damage response (44). Of note, overexpression of USP17 has been observed to result in non-specific deubiquitination of cellular proteins due to the highly active nature of the enzyme (88). Further experiments are therefore required to demonstrate specific interaction of USP17 with H2AK119Ub as well as to clarify why deubiquitination of H2A is beneficial for the lytic virus.

In summary, we identified the chromatin remodeling enzyme CHD4 and components of the PRC1 complex as critical regulators of the latent-lytic EBV switch and showed that during lytic reactivation EBV depletes the PRC1 histone mark (H2AK119Ub) and induces profound upregulation of deubiquitinating enzyme USP17. Overexpressed USP17 de-ubiquitinates H2AK119Ub in co-transfection experiments suggesting that it is likely responsible for H2AK119Ub depletion during lytic EBV infection. Our findings provide important insights into the mechanisms of latency maintenance and lytic reactivation of oncogenic herpesviruses and reveal novel potential targets for therapeutic interventions.

### Limitations of the study

As the PICh method requires a highly abundant target locus, we needed to use an episomal model system of BZLF1 promoter silencing in which the episomes could be maintained at higher copy number than wild-type EBV episomes. The use of HEK 293T cells was therefore a compromise, but as these cells are able to support latent EBV infection and reactivation (89) the silencing mechanisms involved are likely to be similar. The use of EBNA1-OriP to maintain the model system episomes was important to ensure that nuclear location and association with the human genome was representative of latent EBV infection. A previous study using Hi-C sequencing found that plasmids maintained as episomes by the EBNA1-OriP interaction have the same characteristic preference for association with gene-poor, A-T rich areas of the human genome as wild-type EBV (90). Nonetheless, we cannot be certain that the PICh experiment identified cellular proteins which do not normally bind pBZLF1 in the native EBV genome. For example, we identified the MCM complex and TERF2, OriP interacting factors which may have been present due to the close proximity of pBZLF1 and OriP in the model episomes. Likewise, the contribution of EBNA1 to pBZLF1 silencing in the model episomes is uncertain, as we did not determine whether PRC1 and CHD4 occupied pBZLF1 in the absence of EBNA1. Depletion of EBNA1 has previously been shown to provoke spontaneous reactivation of EBV (91), implying a role in maintenance of latency, although presumably through an indirect mechanism as EBNA1 does not bind directly to pBZLF1 (92).

In this study, we identify CHD4 as an essential factor for the maintenance of EBV latency, based on the observation of spontaneous lytic reactivation following CHD4 depletion in Akata cells. Although direct binding of CHD4 to pBZLF1 was strongly suggested by PICh experiments in HEK293T cells, due to technical limitations CHD4 genome occupancy could not be independently confirmed by CHIP-seq or CUT&RUN in B-cells. We therefore cannot exclude the possibility that CHD4 maintains EBV latency in B cells through an indirect mechanism

## Materials and methods

### Cell lines and tissue culture

HEK 293T cells (Lehner Laboratory stock) were maintained in Dulbecco’s Modified Eagle Medium (DMEM, Sigma-Aldrich) plus 10% foetal calf serum (FCS, Gibco) and 100U/ml penicillin/streptomycin (Gibco). Akata and EBV-negative Akata Burkitt lymphoma cell lines were a kind gift from Dr Andrew Bell, University of Birmingham. Both were maintained in RPMI 1640 medium (Sigma-Aldrich) plus 10% FCS and penicillin/streptomycin. Akata cells were stimulated to enter the lytic cycle by crosslinking the B-cell receptor using a goat-anti-human IgG antibody (Cappel, MPBio) at a concentration of between 10-100µg/ml. The antibody was added to the media for 6 hours, then removed by washing twice in phosphate-buffered saline (PBS) and resuspending in fresh media. Subsequent analysis was performed at the timepoints indicated in individual experiments, most commonly at 24 hours. The iSLK.219 endothelial cell line, which harbours latent KHSV and a doxycycline-inducible RTA transgene, was a kind gift from Dr Frank Neipel, University Hospital Erlangen. The cells were maintained in DMEM plus 10% FCS, 1% glutamate (Sigma-Aldrich) and penicillin/streptomycin. To induce KSHV lytic reactivation, doxycycline was added at 1 µg/ml for 24 hours. All cell lines were incubated at 37°C with 5% CO_2_.

### Antibodies

The following primary antibodies were used: goat α−PCGF2 (Abcam ab5267, used for immunoblot), rabbit α−PCGF6 (ProteinTech 24103-1-AP, used for immunoblot), rabbit α-RING1A (Abcam ab32644, used for immunoblot), rabbit α-RING1B (Cell Signalling #5694, used for immunoblot), rabbit α-BCOR (ProteinTech 12107-1-AP, used for immunoblot), rabbit α-EZH2 (Thermo Fisher Scientific 36-6300, used for immunoblot), rabbit α-CHD4 (Abcam ab72418, used for immunoblot), mouse α-BZLF1 (Santa Cruz Biotechnology sc-53904, used for immunoblot and FACS), mouse α-BMRF1 (Santa Cruz Biotechnology sc-58121, used for immunoblot), mouse α-calnexin (AF8, kind gift from M. Brenner, Harvard Medical School, used for immunoblot), mouse α-β-actin (Sigma Aldrich A2228, used for immunoblot), rabbit α-VCP (ProteinTech 10736-1-AP, used for immunoblot), rabbit α-H2AK119Ub (Cell Signalling #8240, used for immunoblot, ChIP-PCR and FACS), rabbit α-H3K27me3 (Cell Signalling #9733, used for immunoblot, ChIP-PCR and FACS), rabbit α-H3K4me3 (Cell Signalling #9751, used for immunoblot, ChIP and FACS), rabbit α-H3K9me3 (Abcam ab8898, used for immunoblot and FACS), rabbit α-Histone H2A (Cell Signalling #12349, used for ChIP), rabbit α-Histone H3 (Abcam ab1791, used for immunoblot and ChIP), rabbit α-phospho-Akt (pAkt) (Cell Signalling #4060, used for FACS), Normal rabbit IgG (Cell Signalling #2729, used for ChIP), mouse α-Myc-tag (Cell Signalling #2276, used for FACS), mouse α-FLAG tag (Invitrogen, MA1-91878, used for FACS). HRP-conjugated secondary antibodies for immunoblot were obtained from Jackson ImmunoResearch. Alexa fluor-conjugated secondary antibodies for FACS were obtained from Thermo Fisher Scientific.

### Plasmid construction

The pBZLF1-GFPx5 reporter was made by cloning the pBZLF1 promoter and GFP coding sequence into the pCEP4 vector (Thermo Fisher Scientific). The pBZLF1 promoter sequence was amplified from an Akata cell DNA extract using PCR with primers TTTGGACGAACTGACCACAA-3’ (forward) and 5’-CTTCAGCAAAGATAGCAAAGGTG-3’ (reverse). pBZLF1 was multimerised to 5 copies using the PCR primers above with the addition of restriction sites for *BsrGI* (TGTACA) on the forward primer and *BsiWI* (CGTACG) on the reverse primer. The pCEP4-pBZLF1x1 plasmid was digested with *BsrGI* (New England Biosciences) and the PCR product ligated in, resulting in a new plasmid with two sequential BZLF1 promoters and a destroyed *BsrGI/BsiWI* hybrid restriction site between them. The process was repeated until five sequential copies of pBZLF1 were present, which was confirmed by digestion of pBZLF1x5-GFP en-bloc followed by gel electrophoresis. The pCEP4-Cas9 vector was generated by cloning the Cas9 sequence from lentiCas9-Blast (Addgene #52962, kindly deposited by Dr Feng Zhang) into the pCEP4 vector using Gibson assembly. The pCEP4-pBMRF1-GFP-P2A-LNGFR-SBP plasmid was made by cloning the BMRF1 promoter and GFP-P2A-LNGFR construct (41) into the pCEP4 vector using Gibson assembly. The 549bp BMRF1 promoter was amplified by PCR from Akata DNA using the primers 5’-CTGCTGATTGAAGGCATCTT-3’ (forward) and 5’-GATCACAAGCAGCAGCAGAAG-3’ (reverse).

### Transfection, electroporation and lentivirus production

HEK 293T cells were transfected using lipofection. For a standard experiment, cells were seeded at 60-80% confluency to a 6-well plate and transfected with 3µg of plasmid DNA using TransIT-293 (Mirus) according to the manufacturer’s instructions. Selection with 50µg/ml hygromycin was performed from 48 hours. Lentivirus was produced by triple-transfection of HEK 293T cells with a lentiviral vector alongside packaging vectors pCMVΔR8.91 and pMD.G. For a standard transfection, cells were seeded to a 6-well plate as above and transfected with a total of 3µg plasmid DNA (comprising 1.5µg of lentiviral vector, 1µg of pCMVΔR8.91 and 0.5µg pMD.G) using TransIT-293. Supernatant containing lentivirus was collected at 48 hours and filtered through an 0.45µm filter (Sartorius Stedim Biotech). The lentivirus was applied to target cells and centrifuged at 1800rpm for 45 minutes. Expression of transgenes was assessed from 48 hours post-transduction.

Akata cells were transfected using a Neon electroporator (Thermo Fisher Scientific) according to the manufacturer’s instructions. For a typical transfection, 2 x 10^6^ cells were washed in PBS and resuspended in 150µL of buffer R with 10µg of plasmid DNA. Electroporation was performed with 1 pulse of 1300V for 30ms, after which cells were resuspended in warmed RPMI with 10% foetal calf serum. Selection with 50µg/ml hygromycin was performed from 48 hours.

### shRNA mediated knockdown of human genes

The pHR-SIREN vector (a generous gift from Greg Towers) was used for lentiviral expression of shRNAs with co-expression of a puromycin resistance gene. Hairpin oligonucleotides were selected from the Broad Institute shRNA library and oligonucleotides purchased from Sigma-Aldrich, then annealed and ligated into the pHR-SIREN vector digested with BamHI and EcoRI (NEB). Successful cloning was confirmed by Sanger Sequencing. The forward shRNA oligonucleotides were: *shScrambled 5’-GAT CCG TTA TAG GCT CGC AAA AGG TTC AAG AGA CCT TTT GCG AGC CTA TAA CTT TTT TG-3’, shRING1A 5’-GAT CCG CCC TGA TCT CTA AGA TCT ATT TCA AGA GAA TAG ATC TTA GAG ATC AGG GCT TTT TTG-3’, shEZH2 5’-GAT CCG CGG CTC CTC TAA CCA TGT TTA TTC AAG AGA TAA ACA TGG TTA GAG GAG CCG TTT TTTG-3’, shCHD4 5’-GAT CCG CCT GCG GAA TGA TAA AGA TAA TTC AAG AGA TTA TCT TTA TCA TTC CGC AGG TTT TTT G-3’, shHDAC2 5’-GAT CCG ACG GTA TCA TTC CAT AAA TAT TCA AGA GAT ATT TAT GGA ATG ATA CCG TCT TTT TTG-3’*

### CRISPR-Cas9 mediated knockdown of human genes

The sgRNAs were designed using the Broad Institute GPP sgRNA Designer Tool and oligonucleotides purchased from Sigma Aldrich. The sgRNA sequences are listed in supplementary table 5. The oligonucleotides were annealed and cloned into the pKLV-U6-sgRNA-PGK-Puro-2A-BFP backbone vector (Addgene #50946, kindly deposited by Dr Kosuke Yusa), which was digested with *BbsI*.

### Human cDNA and EBV lytic gene ORF overexpression

Myc-tagged wild-type USP17 and CS-mutant USP17 cDNAs were a kind gift from Dr Peter Shaw, University of Nottingham. The myc-tagged USP7 cDNA was a kind gift from Dr Roger Everett, University of Glasgow. The cDNAs were overexpressed in HEK 293T cells using lipofection with TransIT-293, as described above. For lentiviral expression of the USP17 cDNAs in Akata cells, the pHRSIN-pSFFV-mCherry-pPGK-PuroR vector was used and USP17 cDNAs co-expressed with the mCherry fluorophore using a P2A sequence. The EBV lytic gene ORFs were overexpressed in HEK 293T cells using lipofection with TransIT-293, as described above.

### Flow cytometry and intranuclear antibody staining

Cells were washed in PBS and then acquired on an LRSFortessa (BD). For intranuclear staining, approximately 1 x 10^6^ cells per condition were resuspended in PBS and fixed in 3.6% formaldehyde for 20 minutes, then permeabilised in 90% methanol at -20°C for 30 minutes. The cells were blocked in PBS with 1% BSA and 5% foetal calf serum for 15 minutes and then primary antibody added at the concentration recommended by the manufacturer for 30 minutes. After washing in PBS, fluorophore-conjugated secondary antibody (typically goat-anti-mouse IgG conjugated to Alexa Fluor 647 or donkey-anti-rabbit IgG conjugated to Alexa Fluor 488 (Thermo Fisher Scientific)) was added at the concentration recommended by the manufacturer for 30 minutes. For all flow cytometry experiments, forward and side scatter gating was performed to exclude dead cells (or cells which had died prior to fixation).

### Purification of lytic Akata cells using Antibody-Free Magnetic Cell Sorting (AFMACS)

Akata cells containing the pBMRF1-GFP-P2A-LNGFR-SBP plasmid were stimulated to enter the lytic cycle using anti-IgG at 100µg/ml. For ChIP-PCR, 200 µM of aciclovir was also added and the cells were incubated for 12 hours. For mass spectrometry, cells were incubated for 24 hours with either 200 µM of aciclovir or 100µg/ml PAA as indicated. Purification of lytic cells expressing cell surface LNGFR-SBP was then performed as described (41) with minor modifications. Cells were washed twice in PBS with 0.5% BSA and resuspended in ice cold incubation buffer (Hank’s Balanced Salt Solution (HBSS – Gibco), 2% dialysed FCS, 1x RPMI Amino Acids (Merck), 1x glutamine (Merck), 2mM EDTA and 10mM HEPES). Dynabeads Biotin Binder magnetic beads (Thermo Fisher Scientific) were equilibrated in incubation buffer and added to the cells at a concentration of 100µL beads per 1 x 10^7^ cells, then incubated at 4°C for 20 minutes with rotation. Beads were immobilised on a magnetic rack and washed twice with incubation buffer, then resuspended in release buffer (RPMI, 10% foetal calf serum and 2mM biotin (Sigma-Aldrich)) for 15 minutes. The eluted cells were then used for either ChIP PCR or mass spectrometry, as described below.

### Proteomics of Isolated Chromatin (PICh) probe design and plasmid capture assay

Eighteen 50-nucleotide 2’ fluoro-RNA probes were designed to tile a 900bp sequence from the pCEP4-pBZLF1-GFP plasmid which centred on pBZLF1, with 300bp of flanking DNA on either side. A nineteenth probe was also designed to cover the join between pBZLF1 sequences due to multimerization of the promoter. Each probe also contained a desthiobiotin molecule appended to the 5’ end via a long spacer arm and a DNA nucleotide ‘cap’ at the 3’ end. The probe sequences are listed in supplementary table 6. For the plasmid capture assay, the pBZLF1x5-GFP segment was excised from the backbone vector using *BssHII* and *NotI* (NEB). 200ng of the plasmid DNA was combined with 0.5µM of PICh probes (an equimolar mix of all 19 probes) in LB3JD buffer (10mM HEPES, 100mM NaCl, 2mM EDTA, 1mM EGTA, 0.2% SDS, 0.1% sarkosyl and an EDTA-free protease inhibitor cocktail tablet (Roche)). The hybridisation reaction was performed in a PCR machine by heating to 90°C for 2 minutes, cooling to 37°C at 1°C per minute, then heating at 37°C for 30 minutes. Seventy five microlitres of Dynabeads MyONE C1 Streptavidin magnetic beads (Thermo Fisher Scientific) were added and incubated for 30 minutes at room temperature with rotation.

Beads were then immobilised on a magnetic stand and supernatant collected as flow-through. Beads were washed five times with LB3JD buffer and then resuspended in 300uL LB3JD and heated for 5 minutes at 37°C. The probe-DNA hybrids were eluted in 100µL of LB3JD containing 12.5mM of D-biotin (Invitrogen) with heating to 65°C for 15 minutes. One tenth of the input, flow through and eluate were analysed by agarose gel electrophoresis with ethidium bromide staining.

### Proteomics of Isolated Chromatin (PICh) method

Chromatin for the PICh experiment was prepared from 4 x 10^9^ HEK 293T cells expressing the pBZLF1-GFP reporter plasmid and also from the same amount of un-transfected control cells. For the transfection, ten 10cm cell culture plates were seeded with HEK 293T cells at approximately 70% confluence and each plate transfected with 40µg of pBZLF1x5-GFP reporter plasmid using TransIT-293. At 48 hours post-transfection, hygromycin was added at 50µg/ml and selection was continued until day 7 when the cells were harvested. Cells were mobilised using Trypsin and transferred to 250ml Corning centrifuge tubes then washed twice with PBS containing 0.1mM PMSF. Fixation was performed using 3.5% formaldehyde in PBS for 30 minutes, after which the cells were washed three times in PBS with 1mM PMSF. The cell pellet was resuspended in sucrose buffer (0.3M sucrose, 10mM HEPES-NaOH pH 7.9, 1% triton X-100, 2mM MgOAc, 1mM PMSF) and lysed with 15 strokes of a 40ml dounce homogeniser. After washing once in PBS with 0.5% Triton X-100, RNase digestion was performed overnight at 4°C with 1mg/ml RNAse A (Qiagen). The following day, the chromatin pellet was washed three times in PBS with PMSF and resuspended in MNase digest buffer (100mM HEPES pH 7.9, 25mM MgCl_2_, 25mM CaCl_2_, 350mM KCl) with 1000U of MNase. The chromatin was digested with MNase at 37°C for 20 minutes to yield predominantly mono- and di-nucleosomal fragments then the reaction was stopped by adding 5mM EGTA and cooling on ice. The chromatin pellet was washed twice in ice cold PBS with 5mM EGTA, then resuspended in LBJD4 sonication buffer (50mM Tris pH 8, 200mM NaCl, 20mM EDTA pH 8, 1% SDS). To solubilise the chromatin, four cycles of sonication (15 sec on/45 sec off) were performed using a Misonix S-4000 probe sonicator in an ice-water slurry. The chromatin was heated to 58°C for 5 minutes in a thermocycler and then centrifuged at 15000xg for 15 minutes at room temperature to pellet the insoluble fragments, which were discarded. The chromatin concentration of each sample was calculated using a NanoDrop spectrophotometer: 20mg of chromatin was used for the pBZLF1 PICh and 10mg for the telomere PICh. The chromatin was pre-cleared by overnight incubation at 4°C with Ultralink streptavidin coated agarose beads (Thermo Fisher Scientific) and de-salted by centrifugation through Sepharose S-400-HR (GE Healthcare) in a Pierce spin column at 750xg for 5 mins. The RNA probes were added to the pre-cleared chromatin at a concentration of 100µM (equimolar mix of all 19 probes). Hybridisation was performed in a PCR machine at 25°C for 3 min, 82°C for 5 min and 37°C for 2 hrs. The probe-chromatin mix was then centrifuged at 18,000xg for 15 mins to pellet any further insoluble chromatin. Pulldown of the probe-DNA hybrids was performed using MyONE C1 streptavidin beads equilibrated in LBJD4 buffer. The chromatin was diluted with 1 volume of milliQ water in a Falcon 15 tube and the beads added then incubated for 2 hours at room temperature with rotation. The beads were immobilised on a magnetic rack and washed five times with 10mls of LB3JD buffer. The beads were then transferred to a 1.5ml microcentrifuge tube and incubated at 42°C for 5 minutes in 1ml of low salt LB3JD buffer containing 50mM NaCl. The probe-DNA hybrids were eluted from the beads by incubation with 900µL of LB3JD buffer containing 12.5mM of biotin for 1 hour at room temperature, followed by heating to 65°C for 15 minutes. The eluted protein was precipitated by adding 20% TCA and incubating on ice for 20 mins, then centrifuged at 16,000xg for 15 minutes at 4°C. The protein pellet was washed twice with acetone at -20°C, then resuspended in crosslinking reversal buffer (250mM Tris pH 8.8, 2% SDS, 0.1M 2-Mercaptoethanol) and heated to 99°C for 25 minutes. After decrosslinking, the protein was subjected to analysis by mass spectrometry as described below.

### Mass spectrometry

For the EBV reactivation proteomics, cells were lysed in 5% SDS/TEAB pH8.5 and digested on S-traps according to the manufactures recommendations (Protify). Subsequently samples were dried and derivatised with TMT reagents according to manufactures recommendations (Thermo Fisher Scientific). Labelled samples were pooled, cleaned up by C18 SPE and subjected to high pH reversed phase fractionation, resulting in 24 fractions. Each fraction was run on a 3h reverse phase gradient on a nano-LCMS system in-line with an Orbitrap Fusion mass spectrometer. For the PICh experiment, proteins were eluted from streptavidin beads and de-crosslinked by heating in an SDS containing buffer. Samples were then subjected to S-trap digestion as above. Digested samples were dried and run on a similar LC-MS setup, except with 1hr runs per sample. All samples were searched using MASCOT (Matrix Science) from within Proteome Discoverer v2.1 (Thermo Fisher Scientific) against the SwissProt Human database and a database of common contaminant proteins. Peptide FDR was controlled against a decoy database search at 1%. For TMT labelled samples reporter ion s/n was extracted and used for quantification. For the PICh experiment, label free quantification based on the top three most abundant peptides for each protein was used.

### Quantitative reverse-transcription PCR (qRT-PCR)

Total RNA was extracted from cells using the RNEasy Plus kit (Qiagen) and RNA concentration calculated using a NanoDrop Spectrophotometer. One microgram of RNA was then reverse transcribed into cDNA using an oligo(dT)_20_ primer (Thermo Fisher Scientific) and SuperScript IV RT reverse transcriptase (Thermo Fisher Scientific) following the manufacturer’s instructions. The qPCR was performed on a Thermo ABI QuantStudio DX Real-Time PCR machine (Applied Biosystems) using SYBR Green Master Mix (Thermo Fisher Scientific). Primers are listed in supplementary table 7. Standard cycling parameters were 50°C for 2 minutes, 95°C for 10 minutes followed by 40 cycles of 95°C for 15 seconds and 58°C for 1 minute. Results were interpreted using the delta-delta C_T_ method and presented as the mean with standard deviation of three replicates. To calculate pBZLF1x5-GFP reporter plasmid copy number, genomic DNA was extracted from HEK 293T cells expressing the reporter plasmid using a DNeasy Blood & Tissue kit (Qiagen). DNA was quantified using a NanoDrop spectrophotometer and then diluted to 8ng/µl (approximately 1000 cells worth of DNA per µl). Quantitation standards with a known plasmid copy number were prepared from miniprep DNA using serial dilution. The cellular DNA and quantitation standards were then run in a qPCR reaction using the cycling parameters described above. The pCEP4 plasmid copy number per cell was calculated based on a curve generated from the quantitation standards using the equation *y=mx+c*.

### Chromatin Immunoprecipitation (ChIP)

ChIP was performed as described previously (93) with minor modifications. 3 x 10^6^ cells were crosslinked in 1% formaldehyde for 10 minutes at room temperature with rotation, then quenched with the addition of 0.125M glycine for 5 minutes. The cells were washed once with ice-cold PBS and then cell lysis buffer added (10mM HEPES, 85mM KCl, 0.5% IGEPAL, EDTA-free protease inhibitor cocktail tablet (Roche)). Nuclei were lysed in nuclei lysis buffer (50mM Tris pH 8.1, 10mM EDTA, 1% SDS, EDTA-free protease inhibitor cocktail tablet) and chromatin sheared using a Bioruptor Pico (Diagenode) to obtain chromatin fragments between 400-600bp in length. The chromatin was diluted 1:10 with IP dilution buffer (20mM Tris pH 8.1, 2mM EDTA, 150mM NaCl, 1% Triton X100, 0.01% SDS, EDTA-free protease inhibitor cocktail tablet) and pre-cleared with Pierce Protein G magnetic beads (Thermo Fisher Scientific) for 2 hours at 4°C with rotation. At this point a small aliquot was removed as the input for subsequent qPCR analysis. For immunoprecipitation, antibodies were added at the concentration recommended by the manufacturer along with fresh Protein G magnetic beads and incubated overnight at 4°C. The beads were immobilised on a magnetic rack and washed five times: twice with low salt buffer (20mM Tris pH 8.1, 2mM EDTA, 50mM NaCl, 1% Triton X100, 0.1% SDS), once with LiCl buffer 910mM Tris pH 8.1, 1mM EDTA, 0.25M LiCl, 1% IGEPAL, 1% sodium deoxycholate monohydrate) and twice with TE buffer (10mM Tris-Cl, 1mM EDTA pH 8.0). The protein-DNA complexes were eluted from the beads in 1% SDS and 150mM NaHCO_3_, and then cross links reversed by addition of 0.3M NaCl and RNAse A with incubation at 67°C overnight. Proteinase K was then added and incubated for 2 hours at 45°C. DNA was purified using a PCR purification kit (Qiagen). Quantification of DNA was performed by qPCR using SYBR Green Master Mix (Thermo Fisher Scientific) on a Thermo ABI QuantStudio DX Real-Time PCR machine (Applied Biosystems) using the primers listed above.

### Immunoblotting

Cells were lysed in 1% SDS in TBS with 1:100 Benzonase and heated to 70°C for 10 minutes. Separation was performed by SDS-PAGE and protein transferred to a PVDF membrane (Millipore). Membranes were blocked in 10% Marvel milk in PBS with 0.2% Tween-20 then probed with the relevant antibodies. Bands were visualised using either ECL, West Pico or West Dura (Thermo Fisher Scientific).

### Statistical analysis

#### PICh data analysis

Analysis of the relative protein abundances from cells with and without the silenced pBZLF1-GFP episome was conducted using Perseus (94). To identify outlier protein abundance ratios, taking into account total protein abundance, Significance B was calculated for each protein (33). Correction for multiple testing was applied using the Benjamini-Hochberg method with FDR <5%. GOCC sub-cellular localisation information was downloaded from Uniprot.

#### TMT-based proteomic data analysis

Statistical analysis was conducted within R using the Bioconductor package LIMMA. P values generated by LIMMA were corrected for multiple hypothesis testing using the Benjamini-Hochberg method (95) to generate an FDR (q-value) for each comparison.

### Analysis of protein-protein association networks using STRING

The significantly enriched proteins from the BZLF1 PICh experiments were analysed using version 12 of STRING (96) with the maximum confidence interaction score and all interaction sources selected except ‘textmining’. The proteins were displayed with ‘kmeans’ clustering.

### Analysis of qPCR data

Data from qPCR experiments was analysed using GraphPad Prism 7 with two-tailed paired Student t-tests performed where indicated.

### Lead contact

Further information and requests for resources and reagents should be directed to and will be fulfilled by the lead contact, Paul Lehner (pjl30@cam.ac.uk)

### Material availability

All unique reagents generated in this study are available upon request.

## Supplementary Material

supp figure legend and tables

supp table 2

supp table 5

supp table 6

## Figures and Tables

**a:**
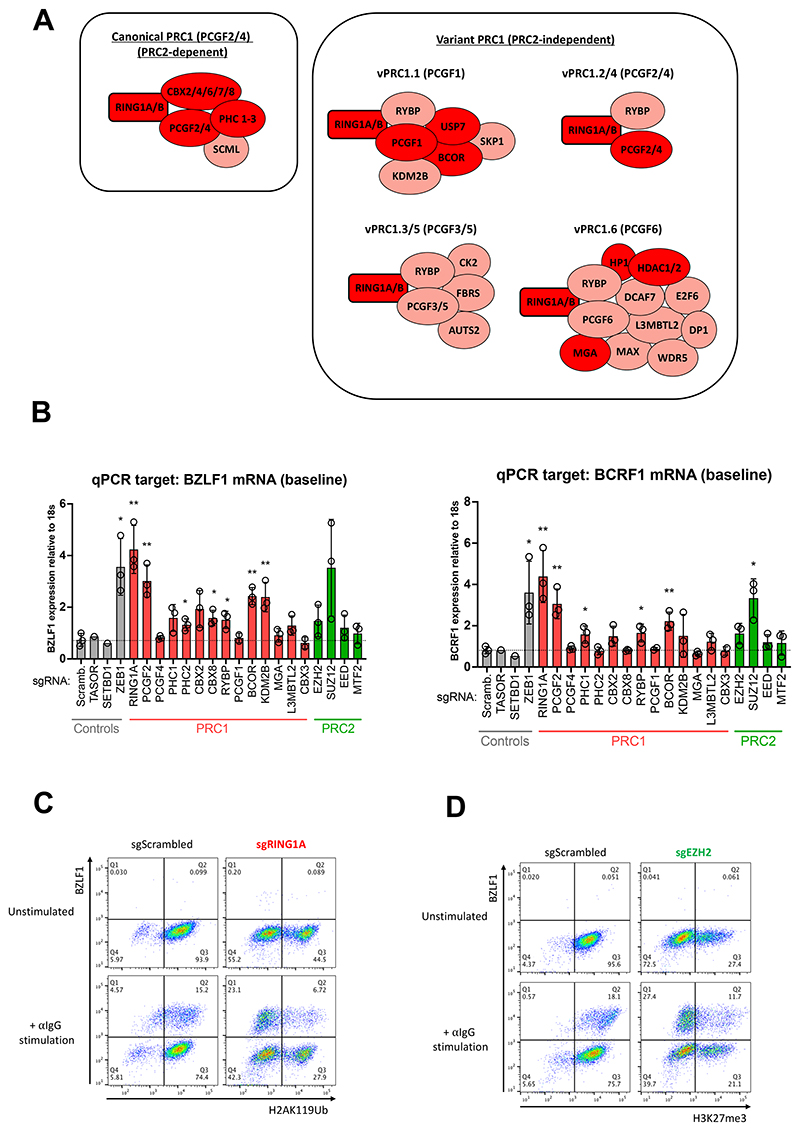


**Figure:**
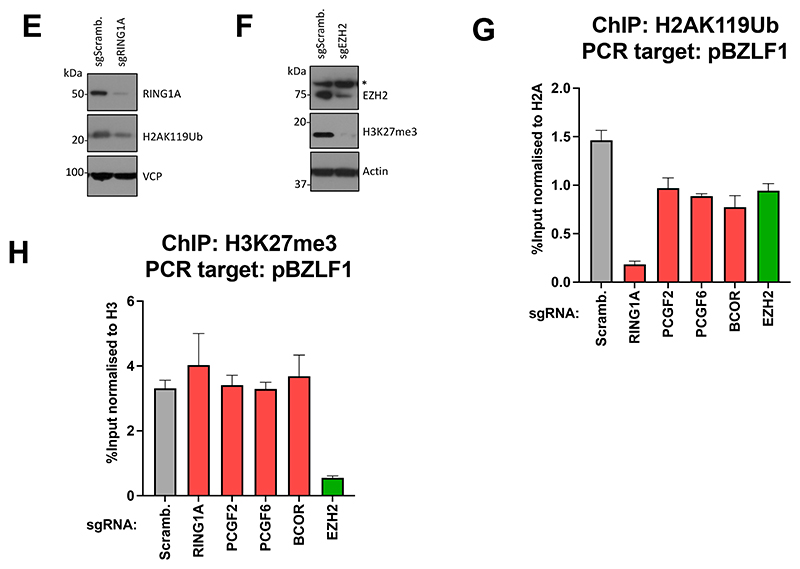


## Data Availability

The mass spectrometry proteomics data have been deposited to the ProteomeXchange Consortium (http://proteomecentral.proteomexchange.org) via the PRIDE partner repository (97) with the dataset identifier PXD068122. These data are licensed under a Creative Commons Zero (CC0) license.

## Importance

Following infection, Epstein-Barr virus persists in a latent state where the viral genome resides in the cell nucleus as an episome. Cellular epigenetic proteins occupy the episomes to restrict viral gene expression and prevent lytic reactivation. In this study, we use mass spectrometry to characterise cellular proteins occupying a key viral lytic gene promoter (pBZLF1). We identify the nucleosome remodeler CHD4 and histone modifier complex PRC1 as novel repressors of pBZLF1 and show that both are required to prevent spontaneous EBV lytic reactivation in B-cells. We also report that PRC1-mediated histone modification is erased during EBV lytic reactivation from both cellular and viral genomes. The human de-ubiquitinase enzyme USP17 is likely to be responsible for this effect, as upregulation of USP17 is induced by EBV lytic proteins. This study provides new insight into how EBV manipulates epigenetic mechanisms to regulate latency and lytic reactivation and reveals novel potential therapeutic targets.
