# Supplementary material for: Locus-specific proteomics identifies novel regulators of Epstein-Barr virus lytic reactivation
Source: J Virol. Author manuscript; Available in PMC 2026 Jun 25. (PMC13098237; doi:10.1128/jvi.01408-25)
Supplement: supp figure legend and tables [file EMS212728-supplement-supp_figure_legend_and_tables.pdf]

Supplementary figure legends and supplementary tables 1,3,4 and 7

Figure S1. Repressed pBZLF1(x5)-GFP reporter is activated by BZLF1 cDNA or histone deacetylase compounds. Related to figure 1. (**A**) Effect of BZLF1 overexpression on pCEP4-pBZLF1(x5)-GFP reporter activity. HEK293T cells containing pCEP4-pBZLF1-GFP episomes (>day 12 post-transfection) were additionally transfected with either BZLF1 cDNA (pHRSIN-pSFFV-BZLF1) or empty control vector and analysed by flow cytometry 24 hours later. (**B**) Silenced pCEP4-pBZLF1(x5)-GFP reporter is activated by histone deacetylase compounds in Burkitt lymphoma cells. EBV-negative Akata cells were electroporated with the pCEP4-pBZLF1(x5)-GFP reporter plasmid followed by hygromycin selection. The cells were allowed to grow out for 14 days followed by stimulation with a combination of PMA, A23187 and TSA for 24 hours and flow cytometry analysis. (**C**) Comparison of pCEP4-pBZLF1(x5)-GFP episomes maintained at steady state by EBV-negative Akata and HEK293T cells. RT-qPCR analysis was performed as described in Figure 1B. Data are presented as mean of $n = 3$ technical replicates ± s.d.

Figure S2. Expression of polycomb PRC1 E3 ubiquitin ligases in human cell lines. Related to Figure 2. HEK293T, Burkitt lymphoma cell lines Raji and Akata (EBV-positive, EBV-negative and EBV-negative transfected with pCEP4-pBZLF1(x5)-GFP plasmid were lysed and subjected to immunoblot analysis with antibodies specific for RING1A, RING1B, H2AK119Ub and VCP.

Figure S3. Chromatin modifications associated with episome-expressed promoters or endogenous genomic loci. Related to figure 2. (**A-D**) ChIP-qPCR using the indicated antibodies comparing episomal PCR targets with genomic loci HOXA10 (positive control for PRC1/2 enrichment/negative control for H3K4me3) and GAPDH (positive control for H3K4me3/negative control for PRC1/2). Data are presented as mean of $n = 3$ technical replicates ± s.d.

Figure S4. PRC1 occupies viral gene promoters along with PRC2 and is required for maintenance of EBV latency in Akata cells. Related to Figure 4. (A) Relative quantification of intracellular EBV genomic DNA in Akata cells with CRISPR-mediated depletion of the components of PRC1 and PRC2 complexes. EBV-positive Akata Cas9+ cells were transduced with lentiviral vectors expressing indicated target-specific sgRNAs, stimulated with anti-IgG antibody for 24 hours, followed by DNA extraction and qPCR analysis with primers specific for EBV *BCLF1* and *GAPDH.* Data are presented as mean of $n = 3$ biological replicates ± s.d. **(B-E)** ChIP qPCR analysis with antibodies specific for PRC1 mark H2AK119Ub (B), PRC2 mark H3K27me3 (C), transcriptionally active chromatin mark H3K4me3 (D), total H3 (E) and IgG control antibody in EBV-positive Akata cells. The qPCR was performed with primers specific for the promoters of immediate-early (pBZLF1 & pBRLF1), early (pBMRF1), late (pGP350) EBV lytic genes, EBV EBNA1 promoters Qp (active in the latency I) and Cp (silent) as well as human control genes HOXA10 (positive control for PRC1/2 silencing) and GAPDH (active). Data are presented as mean of $n = 3$ technical replicates ± s.d. **(F-H)** CRISPR-mediated depletion of PRC1 components in Akata Cas9+ cells. The cells were transduced with lentiviral vectors expressing indicated sgRNAs, lysed and analysed by immunoblot with antibodies specific for PCGF2, PCGF6, BCOR, H2AK119Ub, tubulin and VCP. The cells with the indicated CRISPR gene knockouts were used for the CHIP qPCR experiment presented in Figure 4 (G,H).

Figure S5. Episomal AFMACS system for enrichment of the cells with lytic EBV. Related to Figure 5. (A) Schematic diagram of the pCEP4 episomal vector with pBMRF1-GFP-P2A-LNGFR-SBP cassette (pCEP4-pBMRF1-AFMACS). GFP and LNGFR-SBP are expressed as a single polypeptide separated by a P2A sequence from EBV BMRF1 promoter. **(B)** EBV-positive Akata cells harbouring pCEP4-pBMRF1-AFMACS episomes express GFP upon anti-IgG stimulation. Both wild type (control) and pCEP4-pBMRF1-AFMACS containing EBV-positive Akata cells were incubated with anti-IgG antibody for 24h followed by intranuclear staining with the BZLF1-specific antibody and flow cytometry analysis. **(C)** The pCEP4-pBMRF1-AFMACS system allows isolation of Akata cells with lytic EBV infection. Akata cells harbouring latent EBV and pCEP4-pBMRF1-AFMACS episomes were stimulated with anti-IgG antibody for 12 hours. The cells with lytic EBV (positive for both LNGFR-SBP and GFP) were then isolated using streptavidin magnetic beads. Both unstimulated and anti-IgG stimulated, AFMACS-enriched cells were stained with anti-LNGFR antibody and analysed by flow cytometry. **(D,E)** Analysis of phosphorylated Akt expression (marker of B-cell activation) in EBV-positive Akata cells (D) and EBV-negative Akata cells (E). The cells were stimulated with anti-IgG antibody for 2 hours, fixed, permeabilized, stained with pAkt-specific antibody and analysed by flow cytometry. **(F,G)** ChIP qPCR analysis with antibodies specific for total H3 (F) and H2A (G) histones as detailed in Figure 5. **(H,I)** Analysis of BZLF1 and H2AK119Ub or H3K27me3 mark expression in EBV-positive Akata cells upon anti-IgG stimulation at 6-48 hours (H) or in the presence of aciclovir or PAA compounds (I). The cells were fixed at indicated time points upon stimulation with anti-IgG antibody, stained with antibodies specific for BZLF1 and H2AK119Ub or H3K27me3 and analysed by flow cytometry.

Figure S6. Quantitative proteomics analysis of lytic EBV infection and USP17 expression of in lytic stage of gamma-herpesvirus infection. Related to Figure 6. (A-E) Scatterplots display pairwise comparisons between unstimulated (control) Akata cells with latent EBV infection and cells with lytic (anti-IgG-stimulated, AFMACS-enriched cells) EBV infection in the presence of DMSO, acyclovir (A,C) or PAA (B,D) compounds (added simultaneously with anti-IgG antibody) or a comparison between the cells with lytic EBV infection to which either aciclovir or PAA had been added (E). Each point represents a single protein, plotted by its log2 (fold change in abundance) versus the statistical significance (q value) of that change. The q-value was corrected for multiple hypothesis testing using the method of Benjamini-Hochberg. Dotted line: $q = 0.01$. Human and viral proteins are colour-coded as indicated. **(F)** PRC1 (red dots) and PRC2 (green dots) – related proteins are selectively shown on the scatterplot that displays comparison between latent and lytic EBV infections. **(G)** Human DUBs (de-ubiquitinase enzymes) are highlighted (orange dots) on the scatterplot that displays comparison between latent and lytic EBV infections. **(H-J)** Expression of viral immediate-early *BZLF1* (H), late *BCRF1* (I) lytic and cellular *USP17* (J) gene products in EBV-positive Akata cells following stimulation with anti-IgG antibody in the presence of acyclovir or DMSO control (added simultaneously with anti-IgG antibody). The cells were harvested 24 hours after stimulation, lysed and subjected to RT-qPCR analysis with primers specific for the indicated target genes and *18S.* Data are presented as mean of $n = 3$ technical replicates ± s.d. **(K)** iSLK.219 cells harbouring latent KSHV were treated with doxycycline for 24 hours, lysed and subjected to RNA extraction and RT-qPCR analysis with primers specific for *USP17* and *GAPDH*.

Supplementary table 1: Nuclear proteins identified by the pBZLF1 PICh experiment.

Only proteins with a Significance B value of ≤0.05 represented by ≥2 peptides are shown.

Rank	Protein name	GENE ID	Log2(ratio of abundance in episome vs control cells)	Log10(total abundance)
1	Polycomb group RING finger protein 1	PCGF1	10.09	7.49
2	Telomeric repeat-binding factor 2-interacting protein 1	TE2IP	8.19	6.92
3	MAX gene-associated protein	MGAP	7.54	6.72
4	BCL-6 corepressor	BCOR	6.92	7.74
5	Metal-response element-binding transcription factor 2	MTF2	6.17	7.43
6	Telomeric repeat-binding factor 2	TERF2	5.44	7.09
7	Polyhomeotic-like protein 2	PHC2	5.29	7.99
8	Polycomb group RING finger protein 2	PCGF2	5.28	7.32
9	Polycomb protein EED	EED	4.67	7.58
10	Chromobox protein homolog 8	CBX8	4.59	7.79
11	LEM domain-containing protein 2	LEMD2	4.31	7.35
12	DnaJ homolog subfamily C member 9	DNJC9	4.13	7.21
13	E3 ubiquitin-protein ligase RING2	RING2	4.08	8.25
14	E3 ubiquitin-protein ligase RING1	RING1	3.85	7.14
15	Polycomb protein SUZ12	SUZ12	3.25	7.43
16	Small ubiquitin-related modifier 2	SUMO2	2.61	7.79
17	C-terminal-binding protein 2	CTBP2	2.61	7.83
18	Protein mago nashi homolog 2	MGN2	2.48	7.79
19	Emerin	EMD	2.47	7.84
20	Nuclear pore complex protein Nup98-Nup96	NUP98	2.31	7.24
21	DNA replication licensing factor MCM6	MCM6	2.24	8.33

22	DNA replication licensing factor MCM5	MCM5	2.09	8.22
23	Ubiquitin carboxyl-terminal hydrolase 7	UBP7	2.08	7.49
24	DNA replication licensing factor MCM7	MCM7	2.04	8.28
25	Lamina-associated polypeptide 2, isoform alpha	LAP2A	1.97	8.68
26	Chromobox protein homolog 1	CBX1	1.95	7.62
27	Nuclear pore complex protein Nup155	NU155	1.93	7.52
28	Prelamin-A/C	LMNA	1.92	8.44
29	DNA replication licensing factor MCM2	MCM2	1.91	8.31
30	Histone deacetylase 2	HDAC2	1.89	7.94
31	Chromodomain-helicase-DNA-binding protein 4	CHD4	1.89	8.02
32	Chromobox protein homolog 3	CBX3	1.83	8.27
33	DNA replication licensing factor MCM3	MCM3	1.77	8.48
34	Lamina-associated polypeptide 2, isoforms beta/gamma	LAP2B	1.69	8.30
35	Replication protein A 70 kDa DNA-binding subunit	RFA1	1.66	8.02
36	Lamin-B2	LMNB2	1.59	8.49
37	Transcription intermediary factor 1-beta	TIF1B	1.57	8.53
38	DNA replication licensing factor MCM4	MCM4	1.57	8.33
39	Eukaryotic initiation factor 4A-III	IF4A3	1.48	8.13

Supplementary table 3: Proteins upregulated by ≥3-fold in Akata cells containing lytic EBV vs latent EBV.

Only proteins represented by ≥2 peptides with a q-value of ≤0.01 are shown.

Taxonomy	Protein name	Gene ID	Log_2(Fold change)	p-value	q-value (FDR-corrected p-value)
EBV	Uncharacterized protein LF3	LF3	6.31	1.775E-11	3.744E-08
EBV	Envelope glycoprotein M	gM	5.73	2.222E-09	5.143E-07
EBV	Envelope glycoprotein GP350	GP350	5.14	8.753E-10	2.944E-07
EBV	Protein BMRF2	BMRF2	5.01	3.511E-08	4.807E-06
EBV	Envelope glycoprotein H	gH	4.66	1.124E-11	3.744E-08
EBV	BXRF1	BXRF1	4.66	1.919E-11	3.744E-08
EBV	Tegument protein BRRF2	BRRF2	4.44	9.434E-11	7.159E-08
EBV	Uncharacterized protein BHLF1	BHLF1	4.39	6.019E-08	7.701E-06
EBV	Protein BOLF1	BOLF1	4.34	5.278E-07	4.336E-05
EBV	Protein BDLF2	BDLF2	4.32	9.939E-10	3.103E-07
EBV	Cytoplasmic envelopment protein 2	BGLF2	4.28	9.052E-10	2.944E-07
EBV	Triplex capsid protein 2	TRX2	4.22	4.234E-07	3.763E-05
EBV	Major capsid protein	MCP	4.15	1.138E-09	3.417E-07
EBV	Alkaline exonuclease	BBLF1	4.13	2.675E-07	2.740E-05
EBV	Uracil-DNA glycosylase	UNG	4.05	2.131E-09	5.143E-07
EBV	Thymidine kinase	TK	3.93	4.458E-11	4.436E-08
EBV	BVLF1	BVLF1	3.90	2.125E-09	5.143E-07
EBV	Tegument protein UL51 homolog	BSRF1	3.86	2.192E-07	2.312E-05
EBV	Nuclear egress protein 1	NEC1	3.86	1.009E-10	7.159E-08
EBV	BFRF3	BFRF3	3.86	2.715E-10	1.435E-07
EBV	Cytoplasmic envelopment protein 1	BBRF2	3.85	2.205E-12	1.721E-08
EBV	BDLF3	BDLF3	3.84	3.636E-11	4.436E-08
EBV	Portal protein	BBRF1	3.80	5.716E-09	1.115E-06
EBV	Envelope glycoprotein B	gB	3.79	3.308E-11	4.436E-08
EBV	Protein LF2	LF2	3.72	5.505E-09	1.102E-06
EBV	BGLF3.5	BGLF3.5	3.66	2.146E-04	3.350E-03
EBV	Tegument protein BKRF4	BKRF4	3.64	3.883E-06	1.804E-04
EBV	Triplex capsid protein 1	TRX1	3.57	2.532E-10	1.435E-07

EBV	Deoxyuridine 5'-triphosphate nucleotidohydrolase	DUT	3.52	4.547E-11	4.436E-08
EBV	Large tegument protein deneddylase	BPLF1	3.46	2.198E-10	1.429E-07
EBV	DNA polymerase catalytic subunit	BALF5	3.42	2.068E-09	5.143E-07
EBV	Putative BBLF3 protein (Fragment)	BBLF2-BBLF3	3.37	2.287E-08	3.433E-06
EBV	Replication and transcription activator	BRLF1	3.32	4.422E-10	1.817E-07
Human	Tumor necrosis factor receptor superfamily member 16	NGFR	3.30	2.119E-09	5.143E-07
EBV	BFRF1A	BFRF1A	3.27	2.758E-10	1.435E-07
EBV	Uncharacterized LF1 protein	LF1	3.20	1.027E-08	1.782E-06
EBV	Uncharacterized protein BNLF2b	BNLF2b	3.11	8.971E-07	6.196E-05
EBV	Shutoff alkaline exonuclease	BGLF5	3.05	1.325E-09	3.829E-07
EBV	DNA polymerase processivity factor BMRF1	BMRF1	3.04	4.818E-09	9.896E-07
EBV	mRNA export factor ICP27 homolog	BMLF1	3.04	2.152E-05	6.337E-04
EBV	Uncharacterized protein BTRF1	BTRF1	3.03	3.082E-08	4.373E-06
EBV	Serine/threonine-protein kinase BGLF4	BGLF4	3.02	1.589E-06	9.671E-05
EBV	Uncharacterized protein BDLF4	BDLF4	3.02	7.969E-10	2.827E-07
EBV	Protein BGLF3	BGLF3	3.00	5.334E-10	2.082E-07
Human	Apolipoprotein C-II	APOC2	2.97	1.866E-05	5.850E-04
EBV	Capsid vertex component 2	CVC2	2.93	3.443E-09	7.465E-07
EBV	Protein BOLF1	BOLF1	2.92	3.046E-10	1.448E-07
Human	Thymosin beta-10	TMSB10	2.90	4.131E-04	5.277E-03
EBV	Tripartite terminase subunit 1	TRM1	2.87	2.770E-07	2.771E-05
EBV	Packaging protein UL32 homolog	BFLF1	2.84	1.641E-07	1.779E-05
EBV	Ribonucleoside-diphosphate reductase large subunit	RIR1	2.83	3.873E-07	3.557E-05
Human	Alpha-fetoprotein	AFP	2.83	1.236E-06	7.906E-05
EBV	BSLF1	BSLF1	2.80	5.960E-06	2.578E-04

EBV	BFRF2	BFRF2	2.74	3.247E-07	3.168E-05
Human	Insulin-like growth factor-binding protein 3	IGFBP3	2.72	6.643E-07	5.139E-05
EBV	Trans-activator protein BZLF1	BZLF1	2.65	1.242E-04	2.265E-03
EBV	Transcriptional activator BRRF1	BRRF1	2.64	1.150E-04	2.132E-03
EBV	BcRF1	BcRF1	2.61	6.687E-08	8.155E-06
Human	Histone H2B type 1-A	HIST1H2BA	2.61	4.248E-07	3.763E-05
EBV	Major DNA-binding protein	DBP	2.59	9.229E-09	1.637E-06
EBV	Ribonucleoside-diphosphate reductase small subunit	RIR2	2.58	1.802E-07	1.927E-05
Human	Inter-alpha-trypsin inhibitor heavy chain H2	ITIH2	2.54	1.674E-05	5.354E-04
Human	Alpha-2-HS-glycoprotein	AHSG	2.49	9.066E-05	1.805E-03
Human	Beta-2-glycoprotein 1	APOH	2.45	2.100E-05	6.304E-04
Human	Myc box-dependent-interacting protein 1	BIN1	2.42	1.419E-07	1.560E-05
Human	Ubiquitin carboxyl-terminal hydrolase 17-like protein 5	USP17L5	2.37	3.927E-10	1.703E-07
Human	Tetranectin	CLEC3B	2.34	1.199E-05	4.233E-04
EBV	Putative uncharacterized protein		2.31	3.569E-05	9.163E-04
Human	Kininogen-1	KNG1	2.29	4.553E-04	5.578E-03
Human	Cartilage oligomeric matrix protein	COMP	2.25	6.928E-05	1.501E-03
Human	Vitamin K-dependent protein S	PROS1	2.23	2.412E-05	6.905E-04
Human	Immunoglobulin superfamily containing leucine-rich repeat protein 2	ISLR2	2.19	6.590E-05	1.445E-03
EBV	BBLF4	BBLF4	2.18	2.042E-08	3.187E-06
Human	Complement component C9	C9	2.18	1.873E-06	1.067E-04
Human	Inter-alpha-trypsin inhibitor heavy chain H3	ITIH3	2.15	1.493E-05	4.949E-04
Human	Voltage-gated hydrogen channel 1	HVCN1	2.14	4.066E-04	5.241E-03
Human	Apolipoprotein A-IV	APOA4	2.13	3.742E-06	1.759E-04
Human	Phospholipid transfer protein	PLTP	2.12	1.368E-04	2.422E-03

Human	Histone acetyltransferase KAT2B	KAT2B	2.00	5.750E-04	6.533E-03
Human	Dolichyl-diphosphooligosaccharide--protein glycosyltransferase subunit DAD1	DAD1	1.95	4.878E-04	5.866E-03
Human	Mitochondrial import inner membrane translocase subunit Tim8 A	TIMM8A	1.95	1.607E-04	2.720E-03
Human	Lumican	LUM	1.93	1.345E-04	2.403E-03
Human	L-amino-acid oxidase	IL4I1	1.93	9.918E-06	3.789E-04
Human	Ubiquitin carboxyl-terminal hydrolase 17	USP17L2	1.92	4.708E-04	5.735E-03
Human	Mucin-20	MUC20	1.92	6.369E-08	8.007E-06
Human	Xin actin-binding repeat-containing protein 1	XIRP1	1.92	2.167E-06	1.208E-04
Human	Insulin-like growth factor-binding protein 2	IGFBP2	1.89	9.959E-05	1.924E-03
Human	Transforming growth factor-beta-induced protein ig-h3	TGFBI	1.88	1.595E-05	5.165E-04
Human	Tumor protein 63	TP63	1.88	3.085E-04	4.299E-03
Human	Complement component C7	C7	1.88	4.967E-05	1.186E-03
Human	Zinc finger protein 480	ZNF480	1.87	2.034E-05	6.178E-04
Human	NGFI-A-binding protein 2	NAB2	1.86	2.240E-09	5.143E-07
Human	Neuroblast differentiation-associated protein AHNAK	AHNAK	1.85	1.328E-08	2.254E-06
Human	Cytochrome c oxidase subunit 7C, mitochondrial	COX7C	1.84	2.877E-04	4.136E-03
Human	Thrombospondin-1	THBS1	1.82	2.577E-05	7.289E-04
Human	DENN domain-containing protein 1A	DENND1A	1.81	8.627E-06	3.453E-04
Human	Tyrosine-protein phosphatase non-receptor type 6	PTPN6	1.80	9.588E-07	6.388E-05
EBV	Tripartite terminase subunit 3	TRM3	1.79	6.424E-05	1.427E-03
Human	Collagen alpha-1(VI) chain	COL6A1	1.76	1.174E-05	4.185E-04
Human	Apolipoprotein B-100	APOB	1.75	5.273E-07	4.336E-05

Human	Up-regulated during skeletal muscle growth protein 5	USMG5	1.72	4.061E-05	1.009E-03
Human	Collagen alpha-1(I) chain	COL1A1	1.71	2.844E-06	1.490E-04
EBV	Apoptosis regulator BHRF1	BHRF1	1.69	1.718E-06	1.008E-04
Human	Thymosin beta-4	TMSB4X	1.67	2.349E-04	3.572E-03
Human	Alpha-2-macroglobulin	A2M	1.67	8.042E-05	1.660E-03
Human	Mitochondrial import inner membrane translocase subunit Tim9	TIMM9	1.64	2.319E-04	3.542E-03
Human	Periostin	POSTN	1.62	2.960E-05	7.965E-04
Human	EGF-containing fibulin-like extracellular matrix protein 1	EFEMP1	1.62	2.695E-05	7.407E-04
Human	Phosphatidate phosphatase LPIN1	LPIN1	1.57	7.748E-07	5.652E-05

Supplementary table 4: Proteins downregulated by ≥3-fold in Akata cells containing lytic EBV vs latent EBV.

Only proteins represented by ≥2 peptides with a q-value of ≤0.01 are shown.

Taxonomy	Protein name	Gene ID	Log_2(Fold change)	p-value	q-value (FDR-corrected p-value)
Human	Casein kinase I isoform gamma-2	CSNK1G2	-2.73	2.149E-05	6.337E-04
Human	Bone morphogenetic protein 7	BMP7	-2.72	1.189E-06	7.732E-05
Human	Thioredoxin-interacting protein	TXNIP	-2.72	1.095E-05	3.976E-04
Human	Beta-citrylglutamate synthase B	RIMKLB	-2.67	6.433E-04	7.082E-03
Human	Methylmalonic aciduria and homocystinuria type D protein, mitochondrial	MMADHC	-2.66	1.579E-05	5.158E-04
Human	Mitochondrial import inner membrane translocase subunit Tim17-A	TIMM17A	-2.58	5.953E-05	1.358E-03
Human	Regulator of cell cycle RGCC	RGCC	-2.55	4.330E-04	5.420E-03
Human	G1/S-specific cyclin-E2	CCNE2	-2.54	5.322E-04	6.200E-03
Human	NACHT, LRR and PYD domains-containing protein 11	NLRP11	-2.48	2.703E-07	2.740E-05
Human	MORF4 family-associated protein 1	MRFAP1	-2.47	3.800E-04	4.993E-03
Human	UAP56-interacting factor	FYTTD1	-2.37	1.443E-08	2.396E-06
Human	M-phase inducer phosphatase 2	CDC25B	-2.16	1.692E-06	1.001E-04
Human	Interferon-stimulated 20 kDa exonuclease-like 2	ISG20L2	-2.15	2.110E-06	1.185E-04
Human	Tyrosine-protein kinase Lyn	LYN	-2.14	4.996E-07	4.285E-05
Human	Histone chaperone ASF1B	ASF1B	-2.10	5.177E-06	2.296E-04
Human	Cytohesin-2	CYTH2	-2.07	6.549E-05	1.442E-03
Human	Methyl-CpG-binding domain protein 4	MBD4	-2.03	8.025E-05	1.660E-03
Human	Ribosome production factor 1	RPF1	-2.03	1.837E-06	1.054E-04
Human	N-acetyltransferase ESCO2	ESCO2	-2.01	1.666E-06	9.977E-05
Human	Serine/threonine-protein kinase pim-2	PIM2	-2.01	2.063E-05	6.217E-04
Human	Probable ATP-dependent RNA helicase DDX27	DDX27	-2.01	1.248E-07	1.411E-05

Human	Myc proto-oncogene protein	MYC	-1.92	1.044E-07	1.216E-05
Human	DNA repair and recombination protein RAD54B	RAD54B	-1.89	3.153E-04	4.349E-03
Human	ATP-dependent RNA helicase DDX54	DDX54	-1.88	1.648E-08	2.679E-06
Human	Ribosome biogenesis protein NSA2 homolog	NSA2	-1.87	4.475E-09	9.440E-07
Human	Kelch-like protein 36	KLHL36	-1.87	6.158E-04	6.868E-03
Human	Transcription factor Dp-2	TFDP2	-1.87	5.109E-06	2.279E-04
Human	Mid1-interacting protein 1	MID1IP1	-1.86	1.790E-06	1.035E-04
Human	Inactive ubiquitin carboxyl-terminal hydrolase 53	USP53	-1.85	7.294E-05	1.560E-03
Human	Tastin	TROAP	-1.84	2.824E-04	4.082E-03
Human	Vasculin	GPBP1	-1.82	5.107E-07	4.333E-05
Human	Protein C-ets-1	ETS1	-1.81	1.201E-06	7.748E-05
Human	Ig kappa chain C region	IGKC	-1.80	1.356E-07	1.512E-05
Human	Programmed cell death protein 4	PDCD4	-1.80	2.997E-06	1.539E-04
Human	Protein FAM173A	FAM173A	-1.80	5.811E-04	6.592E-03
Human	Zinc finger C4H2 domain-containing protein	ZC4H2	-1.79	2.252E-05	6.557E-04
Human	pre-rRNA processing protein FTSJ3	FTSJ3	-1.78	2.553E-08	3.759E-06
Human	Active regulator of SIRT1	RPS19BP1	-1.76	6.304E-07	4.978E-05
Human	GrpE protein homolog 2, mitochondrial	GRPEL2	-1.76	4.601E-06	2.088E-04
Human	Ribosome biogenesis protein BMS1 homolog	BMS1	-1.76	4.291E-07	3.763E-05
Human	Nucleolar complex protein 3 homolog	NOC3L	-1.70	9.162E-07	6.196E-05
Human	Multivesicular body subunit 12A	MVB12A	-1.69	6.780E-04	7.339E-03
Human	Ribonucleoside-diphosphate reductase subunit M2	RRM2	-1.69	1.323E-05	4.570E-04
Human	Probable ATP-dependent RNA helicase DDX31	DDX31	-1.68	5.736E-07	4.615E-05
Human	Transmembrane prolyl 4-hydroxylase	P4HTM	-1.65	5.032E-04	6.005E-03
Human	Cell division cycle protein 20 homolog	CDC20	-1.65	5.979E-06	2.578E-04
Human	Isoform E47 of Transcription factor E2-alpha	TCF3	-1.64	1.496E-05	4.949E-04
Human	Isoform 2 of Leukocyte receptor cluster member 8	LENG8	-1.62	1.556E-04	2.652E-03

Human	Ankyrin repeat and SOCS box protein 3	ASB3	-1.61	8.746E-04	8.911E-03
Human	B-cell antigen receptor complex-associated protein beta chain	CD79B	-1.61	2.210E-08	3.382E-06
Human	DNA helicase MCM8	MCM8	-1.60	8.104E-06	3.277E-04
Human	Ribosome biogenesis protein BRX1 homolog	BRIX1	-1.59	1.467E-06	9.232E-05
Human	Cell division control protein 6 homolog	CDC6	-1.59	6.002E-09	1.121E-06
Human	Coiled-coil domain-containing protein 69	CCDC69	-1.58	1.486E-04	2.571E-03
Human	Cell division cycle 7-related protein kinase	CDC7	-1.57	2.642E-06	1.403E-04

Supplementary table 7: primer sequences for RT-qPCR

Target	Sequence	Reference
BZLF1	*F 5'- CTGCTCCTGAGAATGCTT-3', R 5'-CGGCTTGGTTGGTCTGTT-3'*	(Ellis *et al.*, 2010)
BRLF1	*F 5'-CCATACAGGACACAACACCTCA-3', R 5'-ACTCCCGGCTGTAAATTCCT-3'*	This paper
BMRF1	*F 5'-CAACACCGCACTGGAGAG-3', R 5'-GCCTGCTTCACTTTCTTGG-3'*	(McKenzie *et al.*, 2016)
BCRF1	*F 5'-CAGGCCCTGTCAGAAATGAT-3', R 5'-TCCTTTTTCCTGCAGCTTGT-3'*	(McKenzie *et al.*, 2016)
BCLF1 (gDNA)	*F 5'-GCTATCAGGTAACGCAGGAG-3', R 5'-GTTGGTCTGAAGCAGTGTCA-3'*	This paper
USP17	*F 5'-GCCGAACACAGGACCTCTCG-3', R 5'-CGGTGACCTCGGCATCATCC-3'*	This paper
HDAC2	*F 5'-TGACAAACCAGAACACTCCAG-3', R 5'-CTTCTCCATCTTCATCTCCACTG-3'*	This paper
GAPDH	*F 5'-ATGGGGAAGGTGAAGGTCG-3', R 5'-CTCCACGACGTACTCAGCG-3'*	This paper
18s	*F 5'-GTAACCCGTTGAACCCCATT-3', R 5'-CCATCCAATCGGTAGTAGCG-3'*	(Hartenian *et al.*, 2020)
HygroR (gDNA)	*F 5'-CCAGAAGAAGATGTTGGCGA-3', R 5'-GCATAACAGCGGTCATTGAC-3'*	This paper
pBZLF1 (ChIP)	*F 5'-GAGCCACAGGCATTGCTAA-3', R 5'-ACCAGCCTCCTCTGTGATGT-3'*	(Ramasubramanyan *et al.*, 2012)
pBRLF1 (ChIP)	*F 5'-GGCTGACATGGATTACTGGTC-3', R 5'-TGATGCAGAGTCGCCTAATG-3'*	(Ramasubramanyan *et al.*, 2012)
pBMRF1 (ChIP)	*F 5'-CACTGCGGTGGAGGTAGAG-3', R 5'-GGTGGTGTGCCATACAAGG-3'*	(Ramasubramanyan *et al.*, 2012)
pGP350 (ChIP)	*F 5'-CCGGCCCTGGATGACAACAT-3', R 5'-GGCTCCAGGTGGGCATCTTC-3'*	This paper
Cp (ChIP)	*F 5'-AGTTGGTGTAAACACGCCGT-3', R 5'-TCCACCTCTAAGGTCCCACG-3'*	This paper

Qp (ChIP)	*F 5'-CCTGTCACCACCTCCCTGATA-3', R 5'-GGAACACTCCCTCAGTGGTCA-3'*	This paper
HOXA10 (ChIP)	*F 5'-GCCCGTGGAGGCTTCAACAA-3', R 5'-GGCTCCTGGGAGTCTGCTCT-3'*	This paper
pGAPDH (ChIP)	*F 5'-GTGTCCTGCTGCCCACAGTC-3', R 5'-GATTGGCCCGATGGGAGGTG-3'*	This paper
