# Supplementary material for: Locus-specific proteomics identifies novel regulators of Epstein-Barr virus lytic reactivation
Source: J Virol. 2026 Mar 17;100(4):e01408-25. doi: 10.1128/jvi.01408-25 (PMC13098237; doi:10.1128/jvi.01408-25)
Supplement: Supplementary file 1 [file jvi.01408-25-s0007.docx]

**Supplementary figure legends and supplementary tables 1,3,4 and 7**

**Figure S1. Repressed pBZLF1(x5)-GFP reporter is activated by BZLF1 cDNA or histone deacetylase compounds.** Related to figure 1. (**A**) Effect of BZLF1 overexpression on pCEP4-pBZLF1(x5)-GFP reporter activity. HEK293T cells containing pCEP4-pBZLF1-GFP episomes (>day 12 post-transfection) were additionally transfected with either BZLF1 cDNA (pHRSIN-pSFFV-BZLF1) or empty control vector and analysed by flow cytometry 24 hours later. (**B**) Silenced pCEP4-pBZLF1(x5)-GFP reporter is activated by histone deacetylase compounds in Burkitt lymphoma cells. EBV-negative Akata cells were electroporated with the pCEP4-pBZLF1(x5)-GFP reporter plasmid followed by hygromycin selection. The cells were allowed to grow out for 14 days followed by stimulation with a combination of PMA, A23187 and TSA for 24 hours and flow cytometry analysis. (**C**) Comparison of pCEP4-pBZLF1(x5)-GFP episomes maintained at steady state by EBV-negative Akata and HEK293T cells. RT-qPCR analysis was performed as described in Figure 1B. Data are presented as mean of *n* = 3 technical replicates ± s.d.

**Figure S2. Expression of polycomb PRC1 E3 ubiquitin ligases in human cell lines. Related to Figure 2.** HEK293T, Burkitt lymphoma cell lines Raji and Akata (EBV-positive, EBV-negative and EBV-negative transfected with pCEP4-pBZLF1(x5)-GFP plasmid were lysed and subjected to immunoblot analysis with antibodies specific for RING1A, RING1B, H2AK119Ub and VCP.

**Figure S3. Chromatin modifications associated with episome-expressed promoters or endogenous genomic loci. Related to figure 2.** (**A-D**) ChIP-qPCR using the indicated antibodies comparing episomal PCR targets with genomic loci HOXA10 (positive control for PRC1/2 enrichment/negative control for H3K4me3) and GAPDH (positive control for H3K4me3/negative control for PRC1/2). Data are presented as mean of *n* = 3 technical replicates ± s.d.

**Figure S4. PRC1 occupies viral gene promoters along with PRC2 and is required for maintenance of EBV latency in Akata cells. Related to Figure 4. (A)** Relative quantification of intracellular EBV genomic DNA in Akata cells with CRISPR-mediated depletion of the components of PRC1 and PRC2 complexes. EBV-positive Akata Cas9+ cells were transduced with lentiviral vectors expressing indicated target-specific sgRNAs, stimulated with anti-IgG antibody for 24 hours, followed by DNA extraction and qPCR analysis with primers specific for EBV *BCLF1* and *GAPDH*. Data are presented as mean of *n* = 3 biological replicates ± s.d. **(B-E)** ChIP qPCR analysis with antibodies specific for PRC1 mark H2AK119Ub (B), PRC2 mark H3K27me3 (C), transcriptionally active chromatin mark H3K4me3 (D), total H3 (E) and IgG control antibody in EBV-positive Akata cells. The qPCR was performed with primers specific for the promoters of immediate-early (pBZLF1 & pBRLF1), early (pBMRF1), late (pGP350) EBV lytic genes, EBV EBNA1 promoters Qp (active in the latency I) and Cp (silent) as well as human control genes HOXA10 (positive control for PRC1/2 silencing) and GAPDH (active). Data are presented as mean of *n* = 3 technical replicates ± s.d. **(F-H)** CRISPR-mediated depletion of PRC1 components in Akata Cas9+ cells. The cells were transduced with lentiviral vectors expressing indicated sgRNAs, lysed and analysed by immunoblot with antibodies specific for PCGF2, PCGF6, BCOR, H2AK119Ub, tubulin and VCP. The cells with the indicated CRISPR gene knockouts were used for the CHIP qPCR experiment presented in Figure 4 (G,H).

**Figure S5. Episomal AFMACS system for enrichment of the cells with lytic EBV. Related to Figure 5. (A)** Schematic diagram of the pCEP4 episomal vector with pBMRF1-GFP-P2A-LNGFR-SBP cassette (pCEP4-pBMRF1-AFMACS). GFP and LNGFR-SBP are expressed as a single polypeptide separated by a P2A sequence from EBV BMRF1 promoter. **(B)** EBV-positive Akata cells harbouring pCEP4-pBMRF1-AFMACS episomes express GFP upon anti-IgG stimulation. Both wild type (control) and pCEP4-pBMRF1-AFMACS containing EBV-positive Akata cells were incubated with anti-IgG antibody for 24h followed by intranuclear staining with the BZLF1-specific antibody and flow cytometry analysis.  **(C)** The pCEP4-pBMRF1-AFMACS system allows isolation of Akata cells with lytic EBV infection. Akata cells harbouring latent EBV and pCEP4-pBMRF1-AFMACS episomes were stimulated with anti-IgG antibody for 12 hours. The cells with lytic EBV (positive for both LNGFR-SBP and GFP) were then isolated using streptavidin magnetic beads. Both unstimulated and anti-IgG stimulated, AFMACS-enriched cells were stained with anti-LNGFR antibody and analysed by flow cytometry. **(D,E)** Analysis of phosphorylated Akt expression (marker of B-cell activation) in EBV-positive Akata cells (D) and EBV-negative Akata cells (E). The cells were stimulated with anti-IgG antibody for 2 hours, fixed, permeabilized, stained with pAkt-specific antibody and analysed by flow cytometry. **(F,G)** ChIP qPCR analysis with antibodies specific for total H3 (F) and H2A (G) histones as detailed in Figure 5. **(H,I)** Analysis of BZLF1 and H2AK119Ub or H3K27me3 mark expression in EBV-positive Akata cells upon anti-IgG stimulation at 6-48 hours (H) or in the presence of aciclovir or PAA compounds (I).  The cells were fixed at indicated time points upon stimulation with anti-IgG antibody, stained with antibodies specific for BZLF1 and H2AK119Ub or H3K27me3 and analysed by flow cytometry.

**Figure S6. Quantitative proteomics analysis of lytic EBV infection and USP17 expression of in lytic stage of gamma-herpesvirus infection. Related to Figure 6. (A-E)** Scatterplots display pairwise comparisons between unstimulated (control) Akata cells with latent EBV infection and cells with lytic (anti-IgG-stimulated, AFMACS-enriched cells) EBV infection in the presence of DMSO, acyclovir  (A,C) or PAA (B,D) compounds (added simultaneously with anti-IgG antibody) or a comparison between the cells with lytic EBV infection to which either aciclovir or PAA had been added (E). Each point represents a single protein, plotted by its log2 (fold change in abundance) versus the statistical significance (q value) of that change. The q-value was corrected for multiple hypothesis testing using the method of Benjamini-Hochberg. Dotted line: q = 0.01. Human and viral proteins are colour-coded as indicated. **(F)** PRC1 (red dots) and PRC2 (green dots) – related proteins are selectively shown on the scatterplot that displays comparison between latent and lytic EBV infections. **(G)** Human DUBs (de-ubiquitinase enzymes) are highlighted (orange dots) on the scatterplot that displays comparison between latent and lytic EBV infections. **(H-J)** Expression of viral immediate-early *BZLF1* (H), late *BCRF1* (I) lytic and cellular *USP17* (J) gene products in EBV-positive Akata cells following stimulation with anti-IgG antibody in the presence of acyclovir or DMSO control (added simultaneously with anti-IgG antibody). The cells were harvested 24 hours after stimulation, lysed and subjected to RT-qPCR analysis with primers specific for the indicated target genes and *18S.* Data are presented as mean of *n* = 3 technical replicates ± s.d. **(K)** iSLK.219 cells harbouring latent KSHV were treated with doxycycline for 24 hours, lysed and subjected to RNA extraction and RT-qPCR analysis with primers specific for *USP17* and *GAPDH*. 

**Supplementary table 1: Nuclear proteins identified by the pBZLF1 PICh experiment.** 

Only proteins with a Significance B value of ≤0.05 represented by ≥2 peptides are shown. 

| Rank | Protein name | GENE ID | Log2(ratio of abundance in episome vs control cells) | Log10(total abundance) |
| --- | --- | --- | --- | --- |
| 1 | Polycomb group RING finger protein 1 | PCGF1 | 10.09 | 7.49 |
| 2 | Telomeric repeat-binding factor 2-interacting protein 1 | TERF2IP | 8.19 | 6.92 |
| 3 | MAX gene-associated protein | MGA | 7.54 | 6.72 |
| 4 | BCL-6 corepressor | BCOR | 6.92 | 7.74 |
| 5 | Metal-response element-binding transcription factor 2 | MTF2 | 6.17 | 7.43 |
| 6 | Telomeric repeat-binding factor 2 | TERF2 | 5.44 | 7.09 |
| 7 | Polyhomeotic-like protein 2 | PHC2 | 5.29 | 7.99 |
| 8 | Polycomb group RING finger protein 2 | PCGF2 | 5.28 | 7.32 |
| 9 | Polycomb protein EED | EED | 4.67 | 7.58 |
| 10 | Chromobox protein homolog 8 | CBX8 | 4.59 | 7.79 |
| 11 | LEM domain-containing protein 2 | LEMD2 | 4.31 | 7.35 |
| 12 | DnaJ homolog subfamily C member 9 | DNAJC9 | 4.13 | 7.21 |
| 13 | E3 ubiquitin-protein ligase RING2 | RING1B | 4.08 | 8.25 |
| 14 | E3 ubiquitin-protein ligase RING1 | RING1A | 3.85 | 7.14 |
| 15 | Polycomb protein SUZ12 | SUZ12 | 3.25 | 7.43 |
| 16 | Small ubiquitin-related modifier 2 | SUMO2 | 2.61 | 7.79 |
| 17 | C-terminal-binding protein 2 | CTBP2 | 2.61 | 7.83 |
| 18 | Protein mago nashi homolog 2 | MAGOHB | 2.48 | 7.79 |
| 19 | Emerin | EMD | 2.47 | 7.84 |
| 20 | Nuclear pore complex protein Nup98-Nup96 | NUP98 | 2.31 | 7.24 |
| 21 | DNA replication licensing factor MCM6 | MCM6 | 2.24 | 8.33 |
| 22 | DNA replication licensing factor MCM5 | MCM5 | 2.09 | 8.22 |
| 23 | Ubiquitin carboxyl-terminal hydrolase 7 | USP7 | 2.08 | 7.49 |
| 24 | DNA replication licensing factor MCM7 | MCM7 | 2.04 | 8.28 |
| 25 | Lamina-associated polypeptide 2, isoform alpha | LAP2A (TMPO) | 1.97 | 8.68 |
| 26 | Chromobox protein homolog 1 | CBX1 | 1.95 | 7.62 |
| 27 | Nuclear pore complex protein Nup155 | NUP155 | 1.93 | 7.52 |
| 28 | Prelamin-A/C | LMNA | 1.92 | 8.44 |
| 29 | DNA replication licensing factor MCM2 | MCM2 | 1.91 | 8.31 |
| 30 | Histone deacetylase 2 | HDAC2 | 1.89 | 7.94 |
| 31 | Chromodomain-helicase-DNA-binding protein 4 | CHD4 | 1.89 | 8.02 |
| 32 | Chromobox protein homolog 3 | CBX3 | 1.83 | 8.27 |
| 33 | DNA replication licensing factor MCM3 | MCM3 | 1.77 | 8.48 |
| 34 | Lamina-associated polypeptide 2, isoforms beta/gamma | LAP2B (TMPO) | 1.69 | 8.30 |
| 35 | Replication protein A 70 kDa DNA-binding subunit | RPA1 | 1.66 | 8.02 |
| 36 | Lamin-B2 | LMNB2 | 1.59 | 8.49 |
| 37 | Transcription intermediary factor 1-beta | TRIM28 | 1.57 | 8.53 |
| 38 | DNA replication licensing factor MCM4 | MCM4 | 1.57 | 8.33 |
| 39 | Eukaryotic initiation factor 4A-III | EIF4A3 | 1.48 | 8.13 |

**Supplementary table 3:** **Proteins upregulated by ≥3-fold in Akata cells containing lytic EBV vs latent EBV.** 

Only proteins represented by ≥2 peptides with a q-value of ≤0.01 are shown.

| **Taxonomy** | **Protein name** | **Gene ID** | **Log**_**2**_**(Fold change)** | **p-value** | **q-value (FDR-corrected p-value)** |
| --- | --- | --- | --- | --- | --- |
| EBV | Uncharacterized protein LF3 | LF3 | 6.31 | 1.775E-11 | 3.744E-08 |
| EBV | Envelope glycoprotein M | gM | 5.73 | 2.222E-09 | 5.143E-07 |
| EBV | Envelope glycoprotein GP350 | GP350 | 5.14 | 8.753E-10 | 2.944E-07 |
| EBV | Protein BMRF2 | BMRF2 | 5.01 | 3.511E-08 | 4.807E-06 |
| EBV | Envelope glycoprotein H | gH | 4.66 | 1.124E-11 | 3.744E-08 |
| EBV | BXRF1 | BXRF1 | 4.66 | 1.919E-11 | 3.744E-08 |
| EBV | Tegument protein BRRF2 | BRRF2 | 4.44 | 9.434E-11 | 7.159E-08 |
| EBV | Uncharacterized protein BHLF1 | BHLF1 | 4.39 | 6.019E-08 | 7.701E-06 |
| EBV | Protein BOLF1 | BOLF1 | 4.34 | 5.278E-07 | 4.336E-05 |
| EBV | Protein BDLF2 | BDLF2 | 4.32 | 9.939E-10 | 3.103E-07 |
| EBV | Cytoplasmic envelopment protein 2 | BGLF2 | 4.28 | 9.052E-10 | 2.944E-07 |
| EBV | Triplex capsid protein 2 | TRX2 | 4.22 | 4.234E-07 | 3.763E-05 |
| EBV | Major capsid protein | MCP | 4.15 | 1.138E-09 | 3.417E-07 |
| EBV | Alkaline exonuclease | BBLF1 | 4.13 | 2.675E-07 | 2.740E-05 |
| EBV | Uracil-DNA glycosylase | UNG | 4.05 | 2.131E-09 | 5.143E-07 |
| EBV | Thymidine kinase | TK | 3.93 | 4.458E-11 | 4.436E-08 |
| EBV | BVLF1 | BVLF1 | 3.90 | 2.125E-09 | 5.143E-07 |
| EBV | Tegument protein UL51 homolog | BSRF1 | 3.86 | 2.192E-07 | 2.312E-05 |
| EBV | Nuclear egress protein 1 | NEC1 | 3.86 | 1.009E-10 | 7.159E-08 |
| EBV | BFRF3 | BFRF3 | 3.86 | 2.715E-10 | 1.435E-07 |
| EBV | Cytoplasmic envelopment protein 1 | BBRF2 | 3.85 | 2.205E-12 | 1.721E-08 |
| EBV | BDLF3 | BDLF3 | 3.84 | 3.636E-11 | 4.436E-08 |
| EBV | Portal protein | BBRF1 | 3.80 | 5.716E-09 | 1.115E-06 |
| EBV | Envelope glycoprotein B | gB | 3.79 | 3.308E-11 | 4.436E-08 |
| EBV | Protein LF2 | LF2 | 3.72 | 5.505E-09 | 1.102E-06 |
| EBV | BGLF3.5 | BGLF3.5 | 3.66 | 2.146E-04 | 3.350E-03 |
| EBV | Tegument protein BKRF4 | BKRF4 | 3.64 | 3.883E-06 | 1.804E-04 |
| EBV | Triplex capsid protein 1 | TRX1 | 3.57 | 2.532E-10 | 1.435E-07 |
| EBV | Deoxyuridine 5'-triphosphate nucleotidohydrolase | DUT | 3.52 | 4.547E-11 | 4.436E-08 |
| EBV | Large tegument protein deneddylase | BPLF1 | 3.46 | 2.198E-10 | 1.429E-07 |
| EBV | DNA polymerase catalytic subunit | BALF5 | 3.42 | 2.068E-09 | 5.143E-07 |
| EBV | Putative BBLF3 protein (Fragment) | BBLF2-BBLF3 | 3.37 | 2.287E-08 | 3.433E-06 |
| EBV | Replication and transcription activator | BRLF1 | 3.32 | 4.422E-10 | 1.817E-07 |
| Human | Tumor necrosis factor receptor superfamily member 16 | NGFR | 3.30 | 2.119E-09 | 5.143E-07 |
| EBV | BFRF1A | BFRF1A | 3.27 | 2.758E-10 | 1.435E-07 |
| EBV | Uncharacterized LF1 protein | LF1 | 3.20 | 1.027E-08 | 1.782E-06 |
| EBV | Uncharacterized protein BNLF2b | BNLF2b | 3.11 | 8.971E-07 | 6.196E-05 |
| EBV | Shutoff alkaline exonuclease | BGLF5 | 3.05 | 1.325E-09 | 3.829E-07 |
| EBV | DNA polymerase processivity factor BMRF1 | BMRF1 | 3.04 | 4.818E-09 | 9.896E-07 |
| EBV | mRNA export factor ICP27 homolog | BMLF1 | 3.04 | 2.152E-05 | 6.337E-04 |
| EBV | Uncharacterized protein BTRF1 | BTRF1 | 3.03 | 3.082E-08 | 4.373E-06 |
| EBV | Serine/threonine-protein kinase BGLF4 | BGLF4 | 3.02 | 1.589E-06 | 9.671E-05 |
| EBV | Uncharacterized protein BDLF4 | BDLF4 | 3.02 | 7.969E-10 | 2.827E-07 |
| EBV | Protein BGLF3 | BGLF3 | 3.00 | 5.334E-10 | 2.082E-07 |
| Human | Apolipoprotein C-II | APOC2 | 2.97 | 1.866E-05 | 5.850E-04 |
| EBV | Capsid vertex component 2 | CVC2 | 2.93 | 3.443E-09 | 7.465E-07 |
| EBV | Protein BOLF1 | BOLF1 | 2.92 | 3.046E-10 | 1.448E-07 |
| Human | Thymosin beta-10 | TMSB10 | 2.90 | 4.131E-04 | 5.277E-03 |
| EBV | Tripartite terminase subunit 1 | TRM1 | 2.87 | 2.770E-07 | 2.771E-05 |
| EBV | Packaging protein UL32 homolog | BFLF1 | 2.84 | 1.641E-07 | 1.779E-05 |
| EBV | Ribonucleoside-diphosphate reductase large subunit | RIR1 | 2.83 | 3.873E-07 | 3.557E-05 |
| Human | Alpha-fetoprotein | AFP | 2.83 | 1.236E-06 | 7.906E-05 |
| EBV | BSLF1 | BSLF1 | 2.80 | 5.960E-06 | 2.578E-04 |
| EBV | BFRF2 | BFRF2 | 2.74 | 3.247E-07 | 3.168E-05 |
| Human | Insulin-like growth factor-binding protein 3 | IGFBP3 | 2.72 | 6.643E-07 | 5.139E-05 |
| EBV | Trans-activator protein BZLF1 | BZLF1 | 2.65 | 1.242E-04 | 2.265E-03 |
| EBV | Transcriptional activator BRRF1 | BRRF1 | 2.64 | 1.150E-04 | 2.132E-03 |
| EBV | BcRF1 | BcRF1 | 2.61 | 6.687E-08 | 8.155E-06 |
| Human | Histone H2B type 1-A | HIST1H2BA | 2.61 | 4.248E-07 | 3.763E-05 |
| EBV | Major DNA-binding protein | DBP | 2.59 | 9.229E-09 | 1.637E-06 |
| EBV | Ribonucleoside-diphosphate reductase small subunit | RIR2 | 2.58 | 1.802E-07 | 1.927E-05 |
| Human | Inter-alpha-trypsin inhibitor heavy chain H2 | ITIH2 | 2.54 | 1.674E-05 | 5.354E-04 |
| Human | Alpha-2-HS-glycoprotein | AHSG | 2.49 | 9.066E-05 | 1.805E-03 |
| Human | Beta-2-glycoprotein 1 | APOH | 2.45 | 2.100E-05 | 6.304E-04 |
| Human | Myc box-dependent-interacting protein 1 | BIN1 | 2.42 | 1.419E-07 | 1.560E-05 |
| Human | Ubiquitin carboxyl-terminal hydrolase 17-like protein 5 | USP17L5 | 2.37 | 3.927E-10 | 1.703E-07 |
| Human | Tetranectin | CLEC3B | 2.34 | 1.199E-05 | 4.233E-04 |
| EBV | Putative uncharacterized protein |  | 2.31 | 3.569E-05 | 9.163E-04 |
| Human | Kininogen-1 | KNG1 | 2.29 | 4.553E-04 | 5.578E-03 |
| Human | Cartilage oligomeric matrix protein | COMP | 2.25 | 6.928E-05 | 1.501E-03 |
| Human | Vitamin K-dependent protein S | PROS1 | 2.23 | 2.412E-05 | 6.905E-04 |
| Human | Immunoglobulin superfamily containing leucine-rich repeat protein 2 | ISLR2 | 2.19 | 6.590E-05 | 1.445E-03 |
| EBV | BBLF4 | BBLF4 | 2.18 | 2.042E-08 | 3.187E-06 |
| Human | Complement component C9 | C9 | 2.18 | 1.873E-06 | 1.067E-04 |
| Human | Inter-alpha-trypsin inhibitor heavy chain H3 | ITIH3 | 2.15 | 1.493E-05 | 4.949E-04 |
| Human | Voltage-gated hydrogen channel 1 | HVCN1 | 2.14 | 4.066E-04 | 5.241E-03 |
| Human | Apolipoprotein A-IV | APOA4 | 2.13 | 3.742E-06 | 1.759E-04 |
| Human | Phospholipid transfer protein | PLTP | 2.12 | 1.368E-04 | 2.422E-03 |
| Human | Histone acetyltransferase KAT2B | KAT2B | 2.00 | 5.750E-04 | 6.533E-03 |
| Human | Dolichyl-diphosphooligosaccharide--protein glycosyltransferase subunit DAD1 | DAD1 | 1.95 | 4.878E-04 | 5.866E-03 |
| Human | Mitochondrial import inner membrane translocase subunit Tim8 A | TIMM8A | 1.95 | 1.607E-04 | 2.720E-03 |
| Human | Lumican | LUM | 1.93 | 1.345E-04 | 2.403E-03 |
| Human | L-amino-acid oxidase | IL4I1 | 1.93 | 9.918E-06 | 3.789E-04 |
| Human | Ubiquitin carboxyl-terminal hydrolase 17 | USP17L2 | 1.92 | 4.708E-04 | 5.735E-03 |
| Human | Mucin-20 | MUC20 | 1.92 | 6.369E-08 | 8.007E-06 |
| Human | Xin actin-binding repeat-containing protein 1 | XIRP1 | 1.92 | 2.167E-06 | 1.208E-04 |
| Human | Insulin-like growth factor-binding protein 2 | IGFBP2 | 1.89 | 9.959E-05 | 1.924E-03 |
| Human | Transforming growth factor-beta-induced protein ig-h3 | TGFBI | 1.88 | 1.595E-05 | 5.165E-04 |
| Human | Tumor protein 63 | TP63 | 1.88 | 3.085E-04 | 4.299E-03 |
| Human | Complement component C7 | C7 | 1.88 | 4.967E-05 | 1.186E-03 |
| Human | Zinc finger protein 480 | ZNF480 | 1.87 | 2.034E-05 | 6.178E-04 |
| Human | NGFI-A-binding protein 2 | NAB2 | 1.86 | 2.240E-09 | 5.143E-07 |
| Human | Neuroblast differentiation-associated protein AHNAK | AHNAK | 1.85 | 1.328E-08 | 2.254E-06 |
| Human | Cytochrome c oxidase subunit 7C, mitochondrial | COX7C | 1.84 | 2.877E-04 | 4.136E-03 |
| Human | Thrombospondin-1 | THBS1 | 1.82 | 2.577E-05 | 7.289E-04 |
| Human | DENN domain-containing protein 1A | DENND1A | 1.81 | 8.627E-06 | 3.453E-04 |
| Human | Tyrosine-protein phosphatase non-receptor type 6 | PTPN6 | 1.80 | 9.588E-07 | 6.388E-05 |
| EBV | Tripartite terminase subunit 3 | TRM3 | 1.79 | 6.424E-05 | 1.427E-03 |
| Human | Collagen alpha-1(VI) chain | COL6A1 | 1.76 | 1.174E-05 | 4.185E-04 |
| Human | Apolipoprotein B-100 | APOB | 1.75 | 5.273E-07 | 4.336E-05 |
| Human | Up-regulated during skeletal muscle growth protein 5 | USMG5 | 1.72 | 4.061E-05 | 1.009E-03 |
| Human | Collagen alpha-1(I) chain | COL1A1 | 1.71 | 2.844E-06 | 1.490E-04 |
| EBV | Apoptosis regulator BHRF1 | BHRF1 | 1.69 | 1.718E-06 | 1.008E-04 |
| Human | Thymosin beta-4 | TMSB4X | 1.67 | 2.349E-04 | 3.572E-03 |
| Human | Alpha-2-macroglobulin | A2M | 1.67 | 8.042E-05 | 1.660E-03 |
| Human | Mitochondrial import inner membrane translocase subunit Tim9 | TIMM9 | 1.64 | 2.319E-04 | 3.542E-03 |
| Human | Periostin | POSTN | 1.62 | 2.960E-05 | 7.965E-04 |
| Human | EGF-containing fibulin-like extracellular matrix protein 1 | EFEMP1 | 1.62 | 2.695E-05 | 7.407E-04 |
| Human | Phosphatidate phosphatase LPIN1 | LPIN1 | 1.57 | 7.748E-07 | 5.652E-05 |

**Supplementary table 4: Proteins downregulated by ≥3-fold in Akata cells containing lytic EBV vs latent EBV.** 

Only proteins represented by ≥2 peptides with a q-value of ≤0.01 are shown. 

| **Taxonomy** | **Protein name** | **Gene ID** | **Log**_**2**_**(Fold change)** | **p-value** | **q-value (FDR-corrected p-value)** |
| --- | --- | --- | --- | --- | --- |
| Human | Casein kinase I isoform gamma-2 | CSNK1G2 | -2.73 | 2.149E-05 | 6.337E-04 |
| Human | Bone morphogenetic protein 7 | BMP7 | -2.72 | 1.189E-06 | 7.732E-05 |
| Human | Thioredoxin-interacting protein | TXNIP | -2.72 | 1.095E-05 | 3.976E-04 |
| Human | Beta-citrylglutamate synthase B | RIMKLB | -2.67 | 6.433E-04 | 7.082E-03 |
| Human | Methylmalonic aciduria and homocystinuria type D protein, mitochondrial | MMADHC | -2.66 | 1.579E-05 | 5.158E-04 |
| Human | Mitochondrial import inner membrane translocase subunit Tim17-A | TIMM17A | -2.58 | 5.953E-05 | 1.358E-03 |
| Human | Regulator of cell cycle RGCC | RGCC | -2.55 | 4.330E-04 | 5.420E-03 |
| Human | G1/S-specific cyclin-E2 | CCNE2 | -2.54 | 5.322E-04 | 6.200E-03 |
| Human | NACHT, LRR and PYD domains-containing protein 11 | NLRP11 | -2.48 | 2.703E-07 | 2.740E-05 |
| Human | MORF4 family-associated protein 1 | MRFAP1 | -2.47 | 3.800E-04 | 4.993E-03 |
| Human | UAP56-interacting factor | FYTTD1 | -2.37 | 1.443E-08 | 2.396E-06 |
| Human | M-phase inducer phosphatase 2 | CDC25B | -2.16 | 1.692E-06 | 1.001E-04 |
| Human | Interferon-stimulated 20 kDa exonuclease-like 2 | ISG20L2 | -2.15 | 2.110E-06 | 1.185E-04 |
| Human | Tyrosine-protein kinase Lyn | LYN | -2.14 | 4.996E-07 | 4.285E-05 |
| Human | Histone chaperone ASF1B | ASF1B | -2.10 | 5.177E-06 | 2.296E-04 |
| Human | Cytohesin-2 | CYTH2 | -2.07 | 6.549E-05 | 1.442E-03 |
| Human | Methyl-CpG-binding domain protein 4 | MBD4 | -2.03 | 8.025E-05 | 1.660E-03 |
| Human | Ribosome production factor 1 | RPF1 | -2.03 | 1.837E-06 | 1.054E-04 |
| Human | N-acetyltransferase ESCO2 | ESCO2 | -2.01 | 1.666E-06 | 9.977E-05 |
| Human | Serine/threonine-protein kinase pim-2 | PIM2 | -2.01 | 2.063E-05 | 6.217E-04 |
| Human | Probable ATP-dependent RNA helicase DDX27 | DDX27 | -2.01 | 1.248E-07 | 1.411E-05 |
| Human | Myc proto-oncogene protein | MYC | -1.92 | 1.044E-07 | 1.216E-05 |
| Human | DNA repair and recombination protein RAD54B | RAD54B | -1.89 | 3.153E-04 | 4.349E-03 |
| Human | ATP-dependent RNA helicase DDX54 | DDX54 | -1.88 | 1.648E-08 | 2.679E-06 |
| Human | Ribosome biogenesis protein NSA2 homolog | NSA2 | -1.87 | 4.475E-09 | 9.440E-07 |
| Human | Kelch-like protein 36 | KLHL36 | -1.87 | 6.158E-04 | 6.868E-03 |
| Human | Transcription factor Dp-2 | TFDP2 | -1.87 | 5.109E-06 | 2.279E-04 |
| Human | Mid1-interacting protein 1 | MID1IP1 | -1.86 | 1.790E-06 | 1.035E-04 |
| Human | Inactive ubiquitin carboxyl-terminal hydrolase 53 | USP53 | -1.85 | 7.294E-05 | 1.560E-03 |
| Human | Tastin | TROAP | -1.84 | 2.824E-04 | 4.082E-03 |
| Human | Vasculin | GPBP1 | -1.82 | 5.107E-07 | 4.333E-05 |
| Human | Protein C-ets-1 | ETS1 | -1.81 | 1.201E-06 | 7.748E-05 |
| Human | Ig kappa chain C region | IGKC | -1.80 | 1.356E-07 | 1.512E-05 |
| Human | Programmed cell death protein 4 | PDCD4 | -1.80 | 2.997E-06 | 1.539E-04 |
| Human | Protein FAM173A | FAM173A | -1.80 | 5.811E-04 | 6.592E-03 |
| Human | Zinc finger C4H2 domain-containing protein | ZC4H2 | -1.79 | 2.252E-05 | 6.557E-04 |
| Human | pre-rRNA processing protein FTSJ3 | FTSJ3 | -1.78 | 2.553E-08 | 3.759E-06 |
| Human | Active regulator of SIRT1 | RPS19BP1 | -1.76 | 6.304E-07 | 4.978E-05 |
| Human | GrpE protein homolog 2, mitochondrial | GRPEL2 | -1.76 | 4.601E-06 | 2.088E-04 |
| Human | Ribosome biogenesis protein BMS1 homolog | BMS1 | -1.76 | 4.291E-07 | 3.763E-05 |
| Human | Nucleolar complex protein 3 homolog | NOC3L | -1.70 | 9.162E-07 | 6.196E-05 |
| Human | Multivesicular body subunit 12A | MVB12A | -1.69 | 6.780E-04 | 7.339E-03 |
| Human | Ribonucleoside-diphosphate reductase subunit M2 | RRM2 | -1.69 | 1.323E-05 | 4.570E-04 |
| Human | Probable ATP-dependent RNA helicase DDX31 | DDX31 | -1.68 | 5.736E-07 | 4.615E-05 |
| Human | Transmembrane prolyl 4-hydroxylase | P4HTM | -1.65 | 5.032E-04 | 6.005E-03 |
| Human | Cell division cycle protein 20 homolog | CDC20 | -1.65 | 5.979E-06 | 2.578E-04 |
| Human | Isoform E47 of Transcription factor E2-alpha | TCF3 | -1.64 | 1.496E-05 | 4.949E-04 |
| Human | Isoform 2 of Leukocyte receptor cluster member 8 | LENG8 | -1.62 | 1.556E-04 | 2.652E-03 |
| Human | Ankyrin repeat and SOCS box protein 3 | ASB3 | -1.61 | 8.746E-04 | 8.911E-03 |
| Human | B-cell antigen receptor complex-associated protein beta chain | CD79B | -1.61 | 2.210E-08 | 3.382E-06 |
| Human | DNA helicase MCM8 | MCM8 | -1.60 | 8.104E-06 | 3.277E-04 |
| Human | Ribosome biogenesis protein BRX1 homolog | BRIX1 | -1.59 | 1.467E-06 | 9.232E-05 |
| Human | Cell division control protein 6 homolog | CDC6 | -1.59 | 6.002E-09 | 1.121E-06 |
| Human | Coiled-coil domain-containing protein 69 | CCDC69 | -1.58 | 1.486E-04 | 2.571E-03 |
| Human | Cell division cycle 7-related protein kinase | CDC7 | -1.57 | 2.642E-06 | 1.403E-04 |

**Supplementary table 7: primer sequences for RT-qPCR**

| Target | Sequence | Reference |
| --- | --- | --- |
| *BZLF1* | *F 5’- CTGCTCCTGAGAATGCTT-3’, R 5’- CGGCTTGGTTGGTCTGTT-3’* | (Ellis *et al.*, 2010) |
| *BRLF1* | *F 5’-CCATACAGGACACAACACCTCA-3’, R 5’-ACTCCCGGCTGTAAATTCCT-3’* | This paper |
| *BMRF1* | *F 5’-CAACACCGCACTGGAGAG-3’, R 5’-GCCTGCTTCACTTTCTTGG-3’* | (McKenzie *et al.*, 2016) |
| *BCRF1* | *F 5’-CAGGCCCTGTCAGAAATGAT-3’, R 5’-TCCTTTTTCCTGCAGCTTGT-3’* | (McKenzie *et al.*, 2016) |
| *BCLF1 (gDNA)* | *F 5’-GCTATCAGGTAACGCAGGAG-3’, R 5’-GTTGGTCTGAAGCAGTGTCA-3’* | This paper |
| *USP17* | *F 5’-GCCGAACACAGGACCTCTCG-3’, R 5’-CGGTGACCTCGGCATCATCC-3’* | This paper |
| *HDAC2* | *F 5’-TGACAAACCAGAACACTCCAG-3’, R 5’-CTTCTCCATCTTCATCTCCACTG-3’* | This paper |
| *GAPDH* | *F 5’-ATGGGGAAGGTGAAGGTCG-3’, R 5’-CTCCACGACGTACTCAGCG-3’* | This paper |
| *18s* | *F 5’-GTAACCCGTTGAACCCCATT-3’, R 5’-CCATCCAATCGGTAGTAGCG-3’* | (Hartenian *et al.*, 2020) |
| *HygroR (gDNA)* | *F 5’-CCAGAAGAAGATGTTGGCGA-3’, R 5’-GCATAACAGCGGTCATTGAC-3’* | This paper |
| *pBZLF1 (ChIP)* | *F 5’-GAGCCACAGGCATTGCTAA-3’, R 5’-ACCAGCCTCCTCTGTGATGT-3’* | (Ramasubramanyan *et al.*, 2012) |
| *pBRLF1 (ChIP)* | *F 5’-GGCTGACATGGATTACTGGTC-3’, R 5’-TGATGCAGAGTCGCCTAATG-3’* | (Ramasubramanyan *et al.*, 2012) |
| *pBMRF1 (ChIP)* | *F 5’-CACTGCGGTGGAGGTAGAG-3’, R 5’-GGTGGTGTGCCATACAAGG-3’* | (Ramasubramanyan *et al.*, 2012) |
| *pGP350 (ChIP)* | *F 5’-CCGGCCCTGGATGACAACAT-3’, R 5’-GGCTCCAGGTGGGCATCTTC-3’* | This paper |
| *Cp (ChIP)* | *F 5’-AGTTGGTGTAAACACGCCGT-3’, R 5’-TCCACCTCTAAGGTCCCACG-3’* | This paper |
| *Qp (ChIP)* | *F 5’-CCTGTCACCACCTCCCTGATA-3’, R 5’-GGAACACTCCCTCAGTGGTCA-3’* | This paper |
| *HOXA10 (ChIP)* | *F 5’-GCCCGTGGAGGCTTCAACAA-3’, R 5’-GGCTCCTGGGAGTCTGCTCT-3’* | This paper |
| *pGAPDH (ChIP)* | *F 5’-GTGTCCTGCTGCCCACAGTC-3’, R 5’-GATTGGCCCGATGGGAGGTG-3’* | This paper |
